# Estimating DNA methylation potential energy landscapes from nanopore sequencing data

**DOI:** 10.1038/s41598-021-00781-x

**Published:** 2021-11-03

**Authors:** Jordi Abante, Sandeep Kambhampati, Andrew P. Feinberg, John Goutsias

**Affiliations:** 1 Whitaker Biomedical Engineering Institute, Johns Hopkins University, Baltimore, MD 21218 USA; 2 Department of Electrical & Computer Engineering, Johns Hopkins University, Baltimore, MD 21218 USA; 3 Department of Biomedical Engineering, Johns Hopkins University, Baltimore, MD 21205 USA; 4 Center for Epigenetics, Johns Hopkins University School of Medicine, Baltimore, MD 21205 USA; 5 Department of Medicine, Johns Hopkins University School of Medicine, Baltimore, MD 21205 USA; 6 Present Address: Department of Biomedical Data Science, Stanford University School of Medicine, Stanford, CA 94305 USA; 7 Present Address: Department of Biomedical Informatics, Harvard Medical School, Boston, MA 02115 USA

**Keywords:** Epigenomics, Computational models, Data processing, Statistical methods

## Abstract

High-throughput third-generation nanopore sequencing devices have enormous potential for simultaneously observing epigenetic modifications in human cells over large regions of the genome. However, signals generated by these devices are subject to considerable noise that can lead to unsatisfactory detection performance and hamper downstream analysis. Here we develop a statistical method, CpelNano, for the quantification and analysis of 5mC methylation landscapes using nanopore data. CpelNano takes into account nanopore noise by means of a hidden Markov model (HMM) in which the true but unknown (“hidden”) methylation state is modeled through an Ising probability distribution that is consistent with methylation means and pairwise correlations, whereas nanopore current signals constitute the observed state. It then estimates the associated methylation potential energy function by employing the expectation-maximization (EM) algorithm and performs differential methylation analysis via permutation-based hypothesis testing. Using simulations and analysis of published data obtained from three human cell lines (GM12878, MCF-10A, and MDA-MB-231), we show that CpelNano can faithfully estimate DNA methylation potential energy landscapes, substantially improving current methods and leading to a powerful tool for the modeling and analysis of epigenetic landscapes using nanopore sequencing data.

## Introduction

DNA methylation through 5-methylcytosine (5mC) is an important biochemical process that influences biological function in cells by establishing stable and inheritable epigenetic marks throughout the genome^1^. By using a sodium bisulfite treatment and second-generation sequencing, whole-genome bisulfite sequencing (WGBS) generates methylation profiles with comprehensive genomic coverage, high quantitative accuracy, and excellent reproducibility^2^. However, WGBS produces short methylation reads with low contextual information, which limits the scope and effectiveness of downstream analysis^3^.

Nanopore sequencing devices developed by Oxford Nanopore Technologies (ONT) can generate long reads that span thousands of bases. Moreover, 5mC methylation and other epigenetic modifications can be studied using nanopore sequencing without subjecting the DNA to a bisulfite treatment, which is known to be a harsh process that can degrade the DNA. In particular, 5mC marks can be detected from nanopore current signals obtained by using appropriate methylation calling software, such as Nanopolish^4^, DeepMod^5^, DeepSignal^6^, or Megalodon^7^, and provides a decisive edge over short-read bisulfite sequencing in a number of important biological applications^8–10^. For example, nanopore sequencing presents a unique opportunity for studying methylation of transposable elements^11^, a class of repetitive DNA elements that are known to affect proper chromosome function, which cannot be done using short bisulfite reads due to their ambiguous alignment along the genome^12^. It is well-known, however, that detection of 5mC methylation using nanopore sequencing leads to deficient performance due to noise introduced by the sequencer and its underlying chemistry^4^. This issue can seriously affect the output of a comprehensive statistical approach to downstream methylation analysis, since such an approach requires the use of high-order methylation statistics^13–16^ that cannot be reliably estimated from noisy data.

Here we present CpelNano, a method for addressing the statistical challenge described above. To reliably use noisy methylation data obtained from nanopore sequencing, CpelNano employs a data-generative hidden Markov model (HMM) approach which considers the fact that the true methylation state cannot be directly observed by nanopore sequencing (i.e., it is a “hidden” state) but only indirectly through observable data of nanopore current signals. CpelNano models the hidden state through a previously developed parametric model for noiseless data, which leverages an Ising-like correlated potential energy landscape (CPEL) model^13,14^ that is consistent with methylation means and pairwise correlations at each CG dinucleotide (CpG site). This model, which has been successfully used for studying the effect of DNA methyltransferase activity in human embryonic stem cells^17^ and dysregulation of epigenetic landscapes in cancer^18,19^, takes into account evidence suggesting that the likelihood of a given CpG site to be methylated strongly depends on the fraction of CpG sites in a local neighborhood, as well as on the methylation status at nearby CpG sites whose influence diminishes as their nucleotide distance from the given CpG site increases. It then represents the relationship between observed nanopore current signals and the hidden methylation state by means of a set of emission probabilities computed by Nanopolish^4^, the only method that currently quantifies the probability of observing a given set of nanopore current signals associated with a known genetic and epigenetic context. Subsequently, CpelNano estimates the parameters of the methylation potential energy function associated with the CPEL model from noisy nanopore data using the expectation-maximization (EM) algorithm^20^ and performs differential methylation analysis via permutation-based hypothesis testing. Simulations and real data analysis demonstrate the accuracy, effectiveness, and superiority of the proposed statistical method, and show that it can provide a comprehensive and robust framework for the statistical analysis of epigenetic information using nanopore sequencing.

## Results

### Simulations

**Figure 1:**
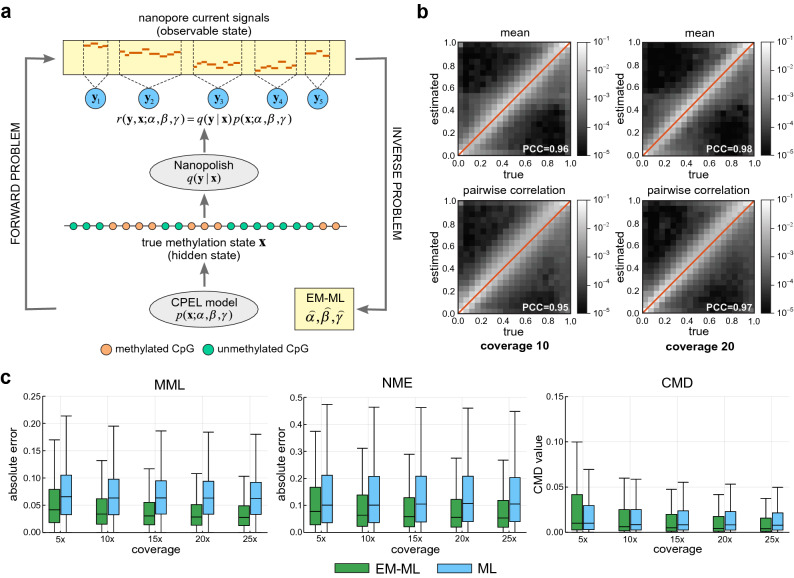
The CpelNano method and simulated performance evaluation results. (**a**) To consider nanopore noise, CpelNano employs a hidden Markov model (HMM) approach, which treats the true methylation state $\pmb {x}$ over an estimation region of the genome as a hidden state that is observed indirectly through a state $\pmb {y}$ of nanopore current signals. It then models the hidden state using a parametric correlated potential landscape model (CPEL) $p(\pmb {x};\alpha ,\beta ,\gamma )$ and addresses the forward problem of modeling the relationship between the observable and hidden methylation states using a data-generative model $r(\pmb {y}, \pmb {x}; \alpha , \beta , \gamma )$ $=$ $q(\pmb {y} \mid \pmb {x}) p(\pmb {x};\alpha ,\beta ,\gamma )$, which is expressed in terms of the CPEL model $p(\pmb {x}; \alpha , \beta , \gamma )$ and emission probabilities $q(\pmb {y} \mid \pmb {x})$ computed using Nanopolish^4^. Finally, it solves the inverse problem of estimating values $\widehat{\alpha }$, $\widehat{\beta }$, and $\widehat{\gamma }$ for the unknown parameters of the CPEL model of the hidden methylation state from available nanopore data using an expectation-maximization based maximum-likelihood (EM-ML) approach. (**b**) Binned joined probability distributions and associated Pearson correlation coefficient (PCC) values between estimated and true means and pairwise correlations at individual CpG sites, obtained by using a simulation-based approach (Fig. S4). Results are shown for nanopore noise with standard deviation $\text {sd}=3$ and data coverages of $10$× and $20$×. A lighter region indicates a higher probability of association between estimated and true values. (**c**) Boxplots depicting distributions of absolute errors over analysis regions between estimated and true mean methylation level (MML) and normalized methylation entropy (NME) values, as well as distributions of coefficient of methylation divergence (CMD) values between the estimated and the true probability distributions of methylation. These quantities were computed by the EM-based maximum-likelihood (EM-ML) approach of CpelNano (green), as well as by fitting the CPEL model directly to the methylation calls made by Nanopolish^4^ using maximum-likelihood (ML; blue). Results are shown for nanopore noise with standard deviation $\text {sd}=3$ and data coverages of $5$×, $10$×, $15$×, $20$×, and $25$×. Center line of box: median value; box bounds: 25th and 75th percentiles; lower whisker: larger of minimum value and 25th percentile minus $1.5$× interquartile range; upper whisker: smaller of maximum value and 75th percentile plus $1.5$× interquartile range.

We present an overview of CpelNano in the “Methods” section and an illustration in Fig. 1a, while providing a more detailed description in the Supplementary Methods. Unlike existing methods for DNA methylation analysis of bisulfite sequencing data, which only address the *inverse problem* of inferring statistical properties of DNA methylation from available data, CpelNano also considers the *forward problem* of predicting the probability distribution of nanopore current signals from a given methylation state. This additional step allows CpelNano to account for nanopore noise and is carried out via a data-generative model expressed in terms of an Ising model for the methylation landscape and emission probabilities computed by Nanopolish^4^.

Since CpelNano relies on Nanopolish^4^, we first evaluated its detection performance by employing a simulation-based benchmarking procedure which we designed using human WGBS and nanopore sequencing data (Supplementary Methods). Notably, the performance of Nanopolish^4^ was previously investigated by using a small number of CpG sites in the *Escherichia coli* reference genome and datasets comprising fully unmethylated or fully methylated CpG sites^4,21^. However, our benchmarking procedure allowed us to provide a comprehensive evaluation of Nanopolish^4^ with more realistic input, including simulated DNA fragments that were hemi-methylated, and assess Nanopolish^4^ over an entire human chromosome (Chr. 22) using four nanopore noise levels. We used different noise levels for two main reasons: first, to demonstrate how methylation calling performance depends on noise level and, second, to identify the actual level of nanopore noise in the data, which is not known.

**Figure 2:**
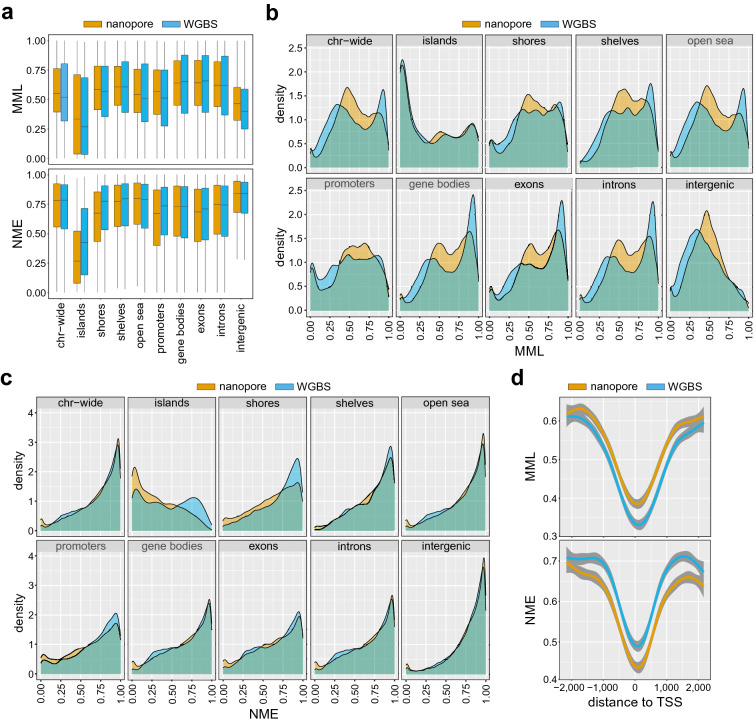
Distributions of methylation levels and entropies in the Utah/Ceph lymphoblastoid cell line. (**a**) Boxplots depicting distributions of mean methylation level (MML) and normalized methylation entropy (NME) values over selected genomic features of the human genome (Chr. 22), estimated from nanopore (brown) and WGBS (blue) data associated with the human Utah/Ceph lymphoblastoid cell line. Center line of box: median value; box bounds: 25th and 75th percentiles; lower whisker: larger of minimum value and 25th percentile minus $1.5$× interquartile range; upper whisker: smaller of maximum value and 75th percentile plus $1.5$× interquartile range. (**b**) Densities of MML values; (**c**) Densities of NME values. (**d**) Aggregate (average) MML and NME values as a function of distance from the transcription start sites (TSSs) of genes.

**Figure 3:**
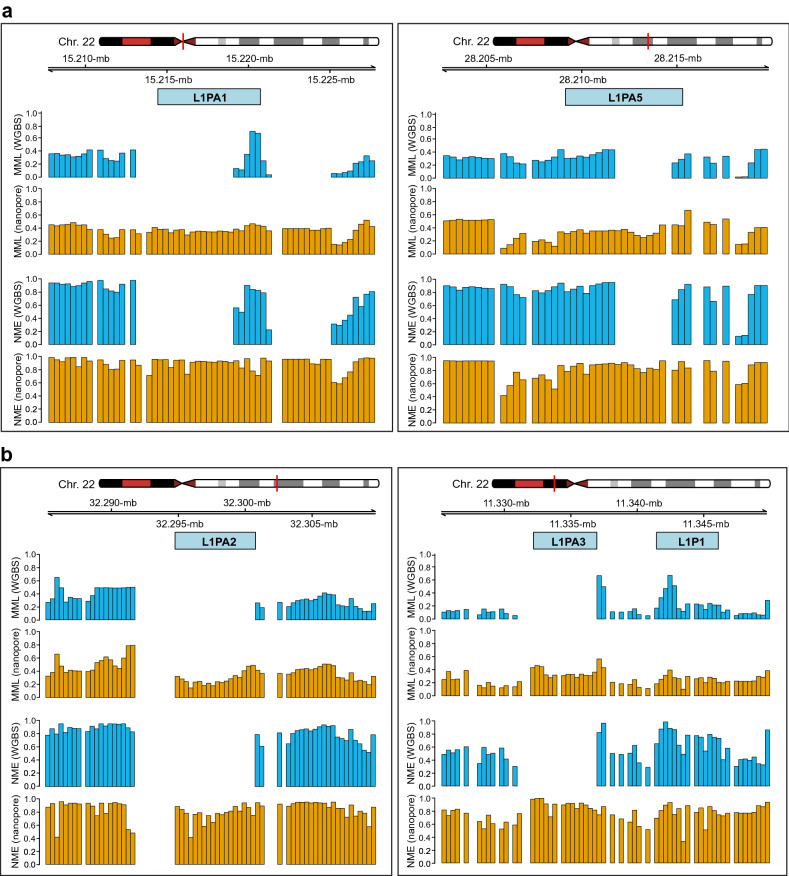
Modeling the DNA methylation landscape over repetitive elements. (**a**) DNA methylation over the L1PA1 and L1PA5 subfamilies of the LINE-1 family of TEs is only partially modeled using WGBS data (GSM2308632) associated with the human Utah/Ceph lymphoblastoid cell line. (**b**) Methylation over the L1PA2 and L1PA3 subfamilies is not modeled using the WGBS data. However, DNA methylation is successfully modeled by CpelNano using the corresponding nanopore data (NA12878).

Our results were similar to those previously achieved when using real data (Figs. S1 and S2), providing additional evidence of deficient detection performance at higher levels of nanopore noise and further showing a trade-off between true positive and false positive rates as well as between precision (probability that a CpG site is correctly predicted to be methylated) and true positive rate (also known as recall). This demonstrates the legitimacy of our benchmarking approach as a convenient and inexpensive computational tool for evaluating the performance of Nanopolish^4^, which can be easily adapted to other nanopore methylation callers if desired. Notably, the receiver operating characteristic (ROC) and precision-recall (PR) curves we obtained for nanopore noise with standard deviation $\text {sd}=3$ (Fig. S2) was similar to the one reported by Simpson et al.^4^ (Fig. 2 corresponding to nanopore chemistry R9 in that paper) and Yuen et al.^21^ (Fig. 3a,b in that paper), suggesting that this level of nanopore noise is close to reality. Importantly, however, our benchmarking results presented evidence (see below) that the statistical properties of DNA methylation cannot be reliably inferred directly from the methylation calls produced by Nanopolish^4^ and clearly demonstrated the effectiveness of CpelNano to deal with this problem.

We first investigated whether we could directly use the methylation calls produced by Nanopolish^4^ to perform downstream statistical analysis that takes into account methylation means at individual CpG sites, as well as pairwise correlations at consecutive CpG sites. As previously argued for the case of WGBS data, this necessitates the use of a stochastic model for the methylation state, such as the CPEL model employed by CpelNano, whose parameters must be estimated from nanopore data with acceptable accuracy. However, accurate parameter estimation requires reliable computation of the sufficient statistics associated with the parameters of the CPEL model (Supplementary Methods) from the methylation calls made by Nanopolish^4^. This depends on faithfully identifying the true methylation state at each CpG site, as well as the true methylation co-occurrence, which identifies pairs of consecutive CpG sites that are both methylated or unmethylated. When the detection threshold used by Nanopolish^4^ was set to zero, our simulations showed an error rate (probability that a CpG site is not correctly predicted to be methylated or unmethylated) in calling the true methylation state at individual CpG sites ranging between $11$ and $16\%$ when $3 \le \text {sd} \le 3.5$ (Fig. S3a). Notably, this rate monotonically decreased to zero with increasing threshold values, but this was achieved by substantially reducing the number of methylation calls made by Nanopolish^4^. For example, to obtain an error rate of $5\%$ (typical to WGBS) for $\text {sd}=3$, our simulations indicated that Nanopolish^4^ must produce methylation calls at only $73\%$ of the CpG sites considered, which is in agreement with Simpson et al.^4^ who reported a $6\%$ error rate using a log-likelihood ratio detection threshold of 2.5 that produced calls at $77\%$ of the targeted CpG sites. Importantly, however, our results (Fig. S3b) showed that, with a zero detection threshold, the error rate in calling the true methylation co-occurrence at pairs of consecutive CpG sites was between $19$ and $27\%$ when $3 \le \text {sd} \le 3.5$ and that this rate remained significant even at high threshold values. This provided evidence that accurate downstream analysis of methylation calls made by Nanopolish^4^ comparable to that of WGBS will require the use of a high detection threshold, which will result in a substantial loss of methylation calls (more than 27% must be discarded) and have significant implications for the quality of downstream methylation analysis, an issue we expect to occur when using other existing nanopore callers, since they have been shown to perform similarly to Nanopolish^21^.

We subsequently carried out simulations to evaluate the performance of the EM-based maximum-likelihood module of CpelNano for estimating the parameters of the CPEL model from nanopore data by modifying the previous benchmarking scheme (“Methods” and Fig. S4). By using cosine similarity distributions, we appraised the closeness of estimated model parameter values to their true values and demonstrated the reliability of this module, even at low coverage (Fig. S5). Remarkably, the median cosine similarity values were close to 1 in all cases considered, implying that parameter estimation performed exceptionally well at least $50\%$ of the time. Moreover, the estimated CPEL models predicted methylation means and pairwise correlations that were mostly associated with small absolute errors (median $< 5\%$ at all noise levels and coverages considered; Figs. S6 and S7, green boxes), considering also the fact that these errors cannot be larger than 1 (“Methods”). On the other hand, estimation of methylation means and pairwise correlations by fitting the CPEL model directly to the methylation calls made by Nanopolish^4^ consistently produced higher errors regardless of the underlying coverage, due to the effect of nanopore noise (Figs. S6 and S7, blue boxes). Notably, and in agreement with previous observations^13^, empirical estimation of methylation means and correlations using the methylation calls made by Nanopolish^4^ led to substantial errors at low coverage (Figs. S6 and S7, red boxes). This was expected since, in addition to not taking into account nanopore noise, empirical methods require substantial amounts of methylation data for reliable estimation, which are not available at low coverage.

Although our results demonstrated diminished estimation performance of the EM-based maximum-likelihood module of CpelNano at increasing levels of nanopore noise, the estimated CPEL models produced reliable estimates for methylation means at individual CpG sites and pairwise correlations, especially at higher coverages (Figs. S6 and S7). These results were also corroborated by plots of binned joint probability distributions between estimated and true values for nanopore noise with standard deviation $\text {sd}=3$ and coverages $10$× and $20$× (Fig. S8), which showed high probabilities for most pairs of estimated vs. true parameter values to be clustered around each plot’s diagonal. However, estimation of the interaction parameter of the CPEL model exhibited a skew towards higher values. We attributed this behavior to a needed assumption that the probability of finding a CG-group (a well-defined genomic region containing a cluster of CpG sites; see Supplementary Methods) with variable methylation in an estimation region is negligible. This is required in order to accommodate the fact that the current version of Nanopolish^4^ assigns the same methylation state at all CpG sites in a CG-group, thus introducing artificially higher pairwise correlation. As a consequence, estimation regions with high proportion of CpG sites in a few CG-groups would be problematic. Nevertheless, given that almost $85\%$ of the CG-groups in the human genome contain only one CpG site and that more than $95\%$ of CG-groups contain at most 2 CpG sites (Fig. S9), very few estimation regions fall into this category. Consequently, our estimation method introduces only a slight bias in the values of the estimated pairwise correlations (Fig. 1b), which can be reduced or even eliminated by better training Nanopolish^4^ to accommodate heterogeneous methylation over estimation regions.

CpelNano partitions each estimation region into the minimum number of equally-sized non-overlapping *analysis regions*, whose size is set by default to be no more than 350 bp (“Methods”), and performs methylation analysis at a resolution of one analysis region. It does so by quantifying the average amount of DNA methylation in each analysis region using the mean methylation level (MML), the amount of methylation stochasticity (variability) using the normalized methylation entropy (NME), and discordance in methylation stochasticity between two methylation landscapes by computing the coefficient of methylation divergence (CMD), an information-theoretic measure of dissimilarity between probability distributions of methylation (“Methods”). By using our simulated nanopore data with the standard deviation of the nanopore noise set to $\text {sd}=3$ and coverages $5$×, $10$×, $15$×, $20$×, $25$×, we sought to evaluate the performance of CpelNano for reliably estimating MMLs, NMEs, and probability distributions of methylation in Chr. 22, and compared the results to those obtained by fitting the CPEL model directly to the methylation calls made by Nanopolish^4^. As expected, CpelNano produced small MML and NME differences, as well as low CMD values, when comparing estimated to true values, especially at higher coverages (Fig. 1c), thus providing strong evidence about its capability of producing reliable estimates of methylation statistics. Notably, fitting the CPEL model directly to the methylation calls made by Nanopolish^4^ produced larger differences in methylation statistics, even at higher coverages. Moreover, Fig. 1c shows that, as coverage increases, CpelNano can reduce the absolute error in estimating statistical properties of the hidden methylation landscape more effectively than when performing methylation analysis directly at the output of Nanopolish^4^. In that sense, CpelNano is capable of efficiently leveraging additional information provided at higher coverages to better estimate the hidden methylation landscape at those coverages.

### Concordance between nanopore and WGBS based estimation of methylation statistics

To further scrutinize CpelNano, we investigated agreement of results obtained from 9112 estimation regions in Chr. 22 by using the publicly available NA12878 (nanopore) and GSM2308632 (WGBS) data identified with the Utah/Ceph lymphoblastoid cell line (“Methods”). MML and NME distributions (Fig. 2a) and densities (Fig. 2b,c) were estimated by CpelNano over selected genomic features and close to transcription start sites of genes (Fig. 2d). The results from the nanopore data were like those obtained from the WGBS data using informME^13,14^, a previously developed powerful approach to methylation analysis. Notably, informME is a special case of CpelNano in the absence of noise, which is approximately the case with WGBS data. Moreover, the results demonstrated known properties of DNA methylation, such as hypomethylation associated with high methylation entropy, an overall reduction in methylation level and entropy over CpG islands (CGIs) when comparing to other genomic features, a bimodal behavior of the methylation level over CGIs towards low and high values, and a progressive reduction of methylation level and entropy closer to transcription start sites.

Although observed dissimilarities, including differences between probability distributions of methylation that were computed from the nanopore and WGBS data using the CMD (Fig. S10), can be attributed to biological, technical, and statistical variability associated with the two methodologies and data used, our results consistently showed a shift of low and high MML values estimated from the WGBS data towards intermediate values when using the nanopore data (Fig. 2b), in agreement with a previous observation^10^. Notably, this behavior can be explained by pointing to recent results obtained by comparing WGBS and methylation array data, which show that, on average, WGBS underestimates methylation levels below 0.5 while it overestimates levels above 0.5 when compared to those measured by more accurate and highly reproducible 450K and EPIC methylation arrays^22^. Markedly, this issue can introduce considerable differences between NME values estimated from nanopore and WGBS data, with the most prominent ones appearing over CGIs, shores, and promoters when using the Utah/Ceph lymphoblastoid cell line (Fig. 2a,c), which are associated with noticeable differences between the probability distributions of methylation observed over these genomic features (Fig. S10). Taken together, these results provide evidence that methylation analysis of nanopore data using CpelNano can produce similar results to those obtained from WGBS data but with the potential of effectively addressing known limitations of whole-genome bisulfite sequencing.

### CpelNano leads to superior methylation analysis of repetitive DNA

An important feature of nanopore sequencing is its potential for detecting base modifications inside long repetitive elements of the genome, known as transposable elements (TEs)^3,23^, which cannot be reliably identified by short-read sequencing technologies^12^. TEs make up a large fraction of the human genome (about $45\%$), whereas their activities can seriously affect cellular function by altering the expression of protein-coding genes and by leading to genomic instability. It is therefore not surprising that aberrant TE transcription has been increasingly linked to many human diseases, including cancer^24–27^.

DNA methylation, along with other epigenetic mechanisms, is known to provide a critical process for silencing TE transcription^28^. This motivated us to investigate the possibility of employing CpelNano and nanopore data to model DNA methylation over TEs and contrast our results to those obtained from WGBS data. To that end, we used the nanopore and WGBS Utah/Ceph lymphoblastoid cell line data, NA12878 and GSM2308632, and compared the results over long interspersed nuclear elements 1 (LINE-1 or simply L1), a family of non-long terminal repeat retrotransposons that constitute about $17\%$ of the human genome^24–26^. We found several examples of L1 subfamilies in Chr. 22, such as L1PA1 (a.k.a. L1HS), L1PA2, L1PA3, and L1PA5, for which modeling the DNA methylation landscape was not successful when using the WGBS data due to ambiguous alignment, despite their high coverage ($\sim \!\! 100$×). Nevertheless, many regions were successfully analyzed by CpelNano using nanopore data. For instance, although DNA methylation over the L1PA1 and L1PA5 subfamilies was only partially modeled using the WGBS data, it was fully modeled by CpelNano using nanopore data (Fig. 3a,b). Moreover, we were not able to model DNA methylation over the L1PA2 and L1PA3 subfamilies using the WGBS data, a problem that was again successfully addressed by CpelNano using the nanopore data (Fig. 3c,d). Notably, the results obtained with CpelNano showed low MMLs over the corresponding retrotransposons and their proximal regions, which were associated with high levels of NME, demonstrating a highly variable DNA methylation landscape.

The previous examples are representative of what one would find when performing genome-wide analysis. Indeed, repetitive DNA sequences are known to frequently result in ambiguous alignments of second-generation sequencing data, which can introduce biases that can affect downstream analysis^12^, and explains our inability to reliably estimate the DNA methylation landscape over long TEs using WGBS. However, nanopore sequencing does not suffer from such issues, given the significantly larger read size produced by this technology. We therefore expect that, by using nanopore sequencing data, we can reliably model and analyze DNA methylation over repetitive regions of the human genome, provided that we use a method, such as CpelNano, which successfully accounts for the effect of noise introduced by the nanopore chemistry on the data.

### Differential methylation analysis of real nanopore data

We further tested and validated CpelNano by performing targeted differential DNA methylation analysis (“Methods”) using real nanopore data and by comparing our results to previously reported findings. Targeted differential analysis is a commonly used approach for evaluating DNA methylation discordance at specific genomic regions of interest that allows for a high depth of coverage, increased statistical power, and reduced sequencing costs. Here, we used publicly available methylation data (“Methods”) recently obtained via nanopore Cas9-targeted sequencing^29^ using the non-tumorigenic epithelial cell line MCF-10A as “normal” and the epithelial human breast cancer cell line MDA-MB-231 (metastatic mammary adenocarcinoma) as “cancer”. These data correspond to genomic regions that fully or partially overlap with the following cancer-associated genes: *BRAF*, *CA9*, *GPX1*, *GSTP1*, *KRAS*, *KRT15*, *KRT19*, *RHOA*, *SLC12A4*, *TP53*, and *TPM2*.

Meaningful statistical evaluation of DNA methylation requires the availability of a sufficient number of replicates, which are currently not available for the previous cell lines. We addressed this issue by randomly partitioning the normal nanopore reads ($271$× median average coverage over 10 CpG sites) into two groups of 5 normal samples, each with an average coverage of $\sim \!25$×, and did similarly with the cancer nanopore reads ($249$× median average coverage over 10 CpG sites) to generate a group of 5 cancer samples (“Methods”). For each analysis region and each sample, we employed CpelNano to compute the MMLs, NMEs, and CMDs from two CPEL models estimated from the nanopore reads using the EM-based maximum-likelihood module. CpelNano compared two groups of methylation summaries by performing (two-tailed) permutation-based hypothesis testing using three differential test statistics. These statistics summarize the differences between the average MML and average NME values in the two groups, as well as the average of all differences between the probability distributions of methylation (quantified by the CMD) observed between the groups (“Methods” and Supplementary Methods).

Computed values of the differential methylation statistics at 480 analysis regions comprising 3086 CpG sites showed considerably larger MML, NME, and CMD values when comparing one of the two normal groups to the cancer group than when comparing the two normal groups to each other (Fig. S11a), presenting the possibility of statistically significant dysregulation of DNA methylation in the cancer samples. Indeed, the computed empirical cumulative probability functions (eCDFs) of the *P*-values obtained for each differential test statistic in the normal/cancer comparison were heavily skewed to the left (Fig. S11b), with many eCDF values being smaller than the significance level used (0.05), and the same was true for the computed *Q*-values (Fig. S11c) obtained by the Benjamini-Hochberg procedure for FDR control, showing that many analysis regions exhibited statistically significant differences in MML, NME, and in the probability distribution of methylation. By comparison, the eCDFs of the *Q*-values obtained in the normal/normal comparison were heavily skewed to the right (Fig. S11c), showing that none of the analysis regions exhibited statistically significant differences, which is expected to be true when using a hypothesis testing procedure that effectively accounts for biological, statistical, and technical variability present in the normal data. Notably, the computed eCDFs for the *P*-values were almost linear (Fig. S11b), implying that the *P*-values were (approximately) uniformly distributed under the null hypothesis, as theoretically expected. Therefore, the probability of observing a *P*-value that is no larger than a given significance level $\alpha$ equals $\alpha$, confirming the theoretical result that the permutation-based hypothesis testing method used by CpelNano properly controls the Type I error, resulting in an error rate that is no more than $5\%$ in a normal/normal comparison ($4.76\%$ to be exact; see Supplementary Methods).

**Figure 4:**
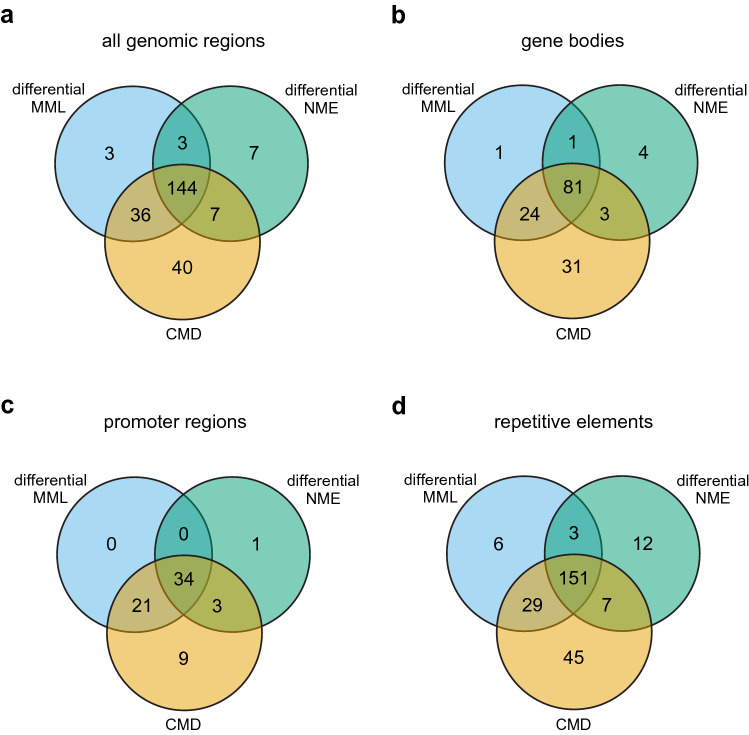
Methylation discordance and analysis regions in the targeted breast normal/cancer comparison. (**a**) Venn diagram showing the number of analysis regions overlapping all genomic regions examined that exhibited significant differences in mean methylation level (MML) and normalized methylation entropy (NME), as well as significant discordance in the probability distribution of methylation quantified by the coefficient of methylation divergence (CMD). (**b**) Venn diagram of significantly dysregulated analysis regions that overlap gene bodies. (**c**) Venn diagram of significantly dysregulated analysis regions that overlap promoter regions. (**d**) Venn diagram of significantly dysregulated analysis regions that overlap known repetitive elements.

We overall found 240 analysis regions exhibiting significant ($q \le 0.05$) dysregulation in DNA methylation, which were associated with significant differential MML (77%), differential NME (67%), and CMD (95%) values (Fig. 4a). Interestingly, 22% of the significantly dysregulated analysis regions did not exhibit significant MML differences, whereas 17% of the significantly dysregulated analysis regions exhibited only significant CMD values and 3% demonstrated only significant differences in NME. This demonstrates the need to use all three test statistics when evaluating DNA methylation discordance between groups. However, our results indicate that the CMD is the most comprehensive quantity for evaluating methylation discordance, since it is associated with 95% of the significantly dysregulated analysis regions. We also obtained similar results over gene bodies and promoter regions (Fig. 4b–d) and acquired detailed associations of types, numbers, and locations of significantly dysregulated analysis regions (Tables S1 and S2). Moreover, we investigated DNA methylation discordance over known repetitive elements along the targeted regions and found many types of repetitive sequences exhibiting significant DNA methylation discordance in breast cancer (Table S3), with $46\%$ of significantly dysregulated analysis regions overlapping Alu elements and $12\%$ overlapping L1 repeats.

**Figure 5:**
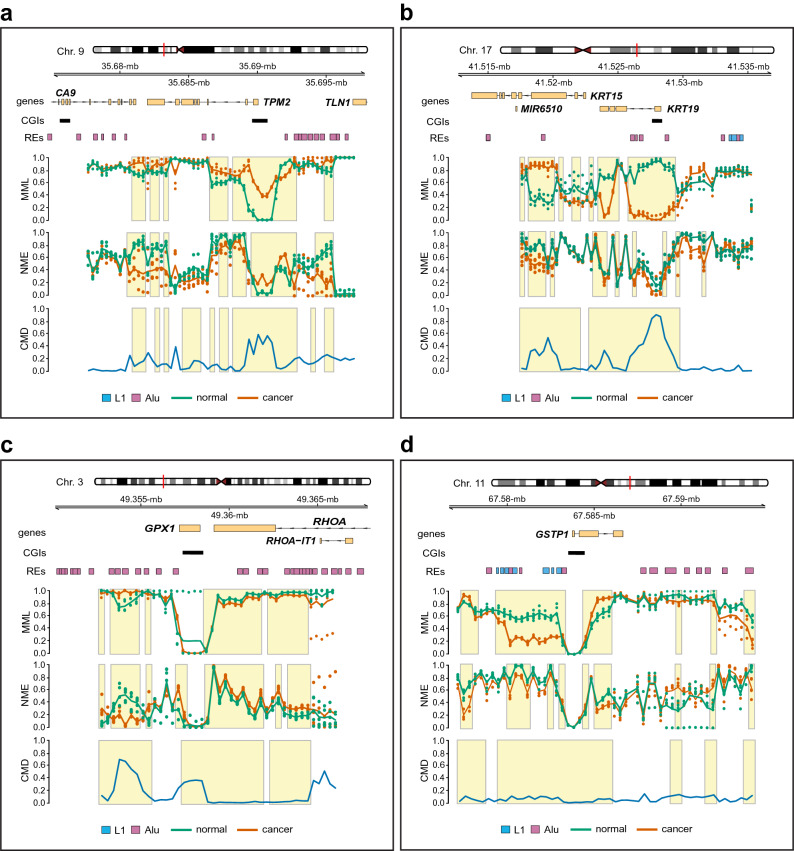
Methylation discordance, genes, and repetitive elements in the targeted breast normal/cancer comparison. (**a**) Averages of mean methylation levels (MMLs) and normalized methylation entropies (NMEs), observed in two groups of five “normal” (green lines) and five “cancer” (red lines) samples used for differential analysis, over genomic regions overlapping *TPM2* and *CA9*. The average of all differences in the probability distributions of methylation between the two groups, quantified by the coefficient of methylation divergence (CMD), is also depicted (blue line). Dots indicate individual MML and NME values for each group and sample, whereas boxes delineate genomic regions of significant ($q \le 0.05$) DNA methylation discordance. CGIs track: CpG islands; REs track: L1 (blue) and Alu (purple) repetitive elements. (**b**) Results of methylation discordance associated with *KRT19* and *KRT15*. (**c**) Results of methylation discordance associated with *GPX1* and *RHOA*. (**d**)  Results of methylation discordance associated with *GSTP1*.

Among the genes that were fully covered by the nanopore data, $\beta$-tropomyosin (*TPM2*), a gene that has been implicated in cell proliferation, migration, and apoptosis, exhibited significant dysregulation of the DNA methylation landscape over its promoter region. This was associated with significant hypermethylation over the gene’s CGI, which was found to be fully unmethylated in the normal group, and a significant increase in methylation entropy, implying increased variability of DNA methylation in breast cancer (Fig. 5a). Interestingly, *TPM2* was recently found to be a tumor suppressor gene whose expression is down-regulated in breast cancer^30^. We also discovered profound changes in the DNA methylation landscape over the promoter region of the cytokeratin-19 (*KRT19*), a coding gene whose CGI was almost fully methylated in normal but exhibited minimal methylation in cancer (Fig. 5b). Notably, DNA hypomethylation and overexpression of *KRT19* has been recently linked to adenocarcinoma^31^, a form of cancer that starts in the epithelial cells that line organs and tissues throughout the body and leads to breast and lung tumors, as well as other types of tumors. Moreover, *KRT19* has been found to be highly upregulated in breast cancer with expression that significantly correlates with cell proliferation, migration, invasion, and prognosis^32–34^.

The breast nanopore data provide full coverage for two additional genes, glutathione peroxidase 1 (*GPX1*) and glutathione S-transferase P1 (*GSTP1*). Despite the fact that both genes have been implicated in certain forms and stages of breast cancer^35,36^, they did not exhibit significant MML or NME discordance over their CGIs, and they were fully unmethylated in both normal and cancer (Fig. 5c,d). Notably, by using bisulfite sequencing, the *GPX1* promoter was also found to be unmethylated in the MDA-MB-453 and BT-474 breast cancer cell lines^36^. Nonetheless, our analysis revealed profound dysregulation of the DNA methylation landscape over a region near the CGI associated with the *GPX1* promoter, linked with significant hypermethylation and loss of entropy (Fig. 5c). Moreover, *GSTP1* exhibited significant hypomethylation over a 4-kb region near its CGI and significant hypermethylation over a portion of its body, which were both associated with a noticeable reduction in methylation entropy (Fig. 5d). Interestingly, aberrant *GSTP1* methylation has been found to be significantly associated with the risk of breast cancer^35^.

Our results also pointed to methylation discordance associated with two additional genes, carbonic anhydrase IX (*CA9*) and cytokeratin-15 (*KRT15*), although it was not possible to provide a complete picture of their methylation status due to incomplete nanopore data covering these genes (Fig. 5a,b). However, *KRT15* exhibited significant dysregulation of the methylation landscape, which was associated with considerable hypermethylation and loss of methylation entropy over a portion of its body. Interestingly, *CA9* has been related to breast cancer and other tumors^37,38^, whereas, *KRT15* was recently found to be hypermethylated and underexpressed in gastric cancer, as well as underexpressed in breast invasive carcinomas, with its expression being significantly associated with overall patient survival in both types of cancer^39,40^. Finally, our analysis produced similar results for the *BRAF*, *KRAS*, *SLC12A4*, and *TP53* genes (Fig. S12), although a full assessment of their methylation status was not possible due to their incomplete nanopore data coverage.

With respect to repetitive elements, CpelNano found four Alu repeats, AluY (314 bp), AluJb (83 bp), Aluz (310 bp), and AluSz (296 bp), at Chr. 17: 41,525,959–41,529,006 near the promoter CGI of *KRT19* exhibiting profound loss of methylation in breast cancer (Fig. 5b and Table S3). This is in agreement with recent results demonstrating early loss of DNA methylation over a small subset of Alu elements in breast cancer^41^. CpelNano also identified three nearby L1 elements, HAL1 (219 bp), L1ME3G (417 bp), and L1ME3G (249 bp), at Chr. 17: 41,533,458–41,534,634, which exhibited high but variable methylation in both normal and cancer, a methylation state that is common to most L1 retrotransposons^24^. Interestingly, we found a cluster of five Alu elements, AluY (297 bp), AluSx1 (301 bp), AluYb8 (318 bp), AluSx1 (307 bp), and AluSx1 (276 bp), at Chr. 3: 49,353,360–49,355,409 near *GPX1*, exhibiting hypermethylation and loss of methylation entropy in breast cancer (Fig. 5c and Table S3). Finally, CpelNano identified a cluster of seven L1 elements, L1MEh (159 bp), L1MEh (258 bp), L1MEh (267 bp), L1MEh (296 bp), L1PA14 (357 bp), L1M5 (182 bp), and L1PA11 (354 bp), separated by three Alu repeats, AluSq (293 bp), AluJb (139 bp), and AluSx (277 bp), at Chr. 11: 67,579,281–67,583,297 near the CGI associated with *GSTP1* showing considerable hypomethylation and noticeable entropy reduction in breast cancer (Fig. 5d and Table S3). This concurs with emerging evidence that hypomethylation of L1 elements is an early event in carcinogenesis that leads to aberrant transcription activation and chromosomal instability in many types of cancer^42^, including breast cancer^43,44^.

Taken together, the previous results show remarkable consistency with known biological evidence and demonstrate the effectiveness of CpelNano for generating a comprehensive description of DNA methylation discordance at high resolution using nanopore data. Evidently, this is also true at regions of the genome rich in repetitive elements, which are difficult to map and study using short-read sequencing technologies.

## Discussion

A statistically robust method for methylation analysis of nanopore data must account for the presence of noise, intrinsically introduced by the nanopore chemistry, which can significantly affect the reliability of downstream analysis and lead to non-reproducible results. In contrast to existing methods of methylation analysis, CpelNano addresses this problem by using a data-generative hidden Markov model (HMM) that employs a previously introduced Ising model to characterize the true DNA methylation state as a “hidden” state and appropriate emission probabilities, computed via Nanopolish^4^, to account for the presence of noise. By performing realistic simulations and analysis of real data, we have shown the utility of CpelNano as a comprehensive and reliable method for DNA methylation analysis of nanopore data that allows for reliable statistical analysis of the methylation state even in regions of the genome that are hard to map when using bisulfite sequencing, such as repetitive elements. Moreover, we have demonstrated the superiority of CpelNano over approaches that directly estimate methylation statistics from erroneous methylation states detected when using nanopore-based methylation callers.

In this paper, we used CpelNano to carry out differential methylation analysis in an unmatched sample pairs group comparison by permuting the group labels of nanopore data samples. However, the current version of CpelNano can also be used for a matched sample pairs group comparison (Supplementary Methods), a common experimental design that is particularly useful when an unbiased assessment of differential methylation is of interest. In addition, CpelNano can be used for a two-sample comparison by performing permutations on the sample labels of nanopore reads (Supplementary Methods). This experimental design is quite useful in a clinical setting, which is often characterized by a lack of replicates or when performing allele-specific methylation analysis, an important area of epigenetic research in which third-generation nanopore sequencing has a clear advantage over older technologies^10^. Finally, CpelNano has been implemented in a user-friendly Julia package, which allows the user to perform DNA methylation analysis by writing a few lines of code. Given its ease of use, statistical reliability, and versatility in terms of experimental design, we believe that CpelNano will become very useful in the epigenetic nanopore community.

In the future, the issue of intrinsic noise affecting third-generation sequencing could become more prominent if the nanopore chemistry is upgraded to include more efficient and faster motor enzymes in an effort to increase sequencing speed. This is because increasing the speed with which a DNA molecule passes through a nanopore may effectively reduce the number of current measurements, resulting in significantly higher levels of noise at the output of the sequencer. While existing analysis methods will be highly unreliable in this case, we expect CpelNano to still be effective, requiring only a minor update of the emission probabilities, which can be accomplished by retraining Nanopolish^4^ using the new nanopore chemistry.

In its present form, CpelNano can only model nearest-neighbor methylation interactions along the genome. However, nanopore sequencing offers the possibility of simultaneously observing DNA methylation at genomically distant CpG sites, due to the significantly large size of its reads^3,10^. This is a natural approach for capturing recently revealed coordination of DNA methylation activities between genomically distant but spatially proximal regions of the genome^45^, which is not possible when using bisulfite sequencing. Improving CpelNano with respect to this issue will entail, for example, extending the current CPEL model of the hidden methylation state to a long-range ferromagnetic Ising model^46^. In this model, the “interaction” term associated with its potential energy function decays to zero at increasing genomic distances between CpG sites in a much slower rate than in the current CPEL model, allowing for interactions beyond nearest-neighbor CpG sites. Nevertheless, such an extension can lead to serious statistical and computational challenges, which will require a significant amount of experimental data and computational resources to achieve meaningful results. Some of these issues, however, could most likely be addressed by using epigenetic data from additional experimental modalities, such as HiC. These data can provide prior information about chromatin structure and organization that is necessary for constructing a more suitable statistical model for the hidden methylation state than the CPEL model considered in this paper to capture long-range correlations in methylation.

Overall, we have demonstrated in this paper that CpelNano is a versatile and innovative method for DNA methylation analysis of nanopore data that provides substantial improvements over currently available approaches. Moreover, this method establishes a blueprint for developing new statistical approaches for the analysis of epigenetic information using third-generation sequencing and opens the possibility of new applications in the field of epigenetics. In fact, third-generation sequencing provides a unique opportunity for studying other epigenetic marks, such as 5-hydroxymethylcytosine (5hmC) and 5-formylcytosine (5fC), among others, which play a functional role in DNA demethylation, cell differentiation, gene transcription, and chromatin regulation^47–49^ but are yet to be thoroughly investigated and characterized^3^. The work presented in this paper offers a solid foundation upon which computational analysis of such epigenetic marks can be jointly performed by simultaneously observing their states over large or previously unmapped regions of the genome. We therefore believe that CpelNano can enable and accelerate both basic and clinical research in several new directions.

## Methods

### CPEL model estimation

The first step of CpelNano is the estimation of the parameters of the underlying CPEL model from nanopore data. To that effect, and in agreement with previous work^13,14^, each chromosome is partitioned into appropriately defined non-overlapping *estimation regions*. Parameter estimation is then performed by a maximum-likelihood estimation method, implemented by means of the expectation-maximization (EM) algorithm, which takes into account the availability of multiple independent nanopore reads as well as nanopore noise. This method operates in conjunction with Nanopolish^4^ (v0.13.2; https://github.com/jts/nanopolish/), which computes the necessary information required for evaluating the likelihood of observed nanopore current signals.

### Analysis regions

To facilitate analysis of methylation information, CpelNano partitions each estimation region into the minimum number of equally-sized non-overlapping *analysis regions*, whose size is no more than a given maximum size $s_{\max}$, and performs methylation analysis at a resolution of one analysis region. The value of $s_{\max}$ was determined by balancing two competing interests while reducing variation in the sizes of the resulting analysis regions: first, methylation analysis must be performed at high resolution by reducing the size of the analysis regions and, second, the size of the analysis regions must be expanded in order to increase the number of analysis regions that contain more than one CpG site in order to account for the effect of pairwise correlations. Computation of histograms of CpG site populations within analysis regions in the human genome for different values of $s_{\max}$ revealed that the majority of the analysis regions contained more than one CpG site when $s_{\max} = 350\,$ bp while the resulting sizes were closely clustered around this value, thus satisfying both requirements (Figs. S13 and S14).

### Methylation analysis

To perform methylation analysis, CpelNano quantifies the average amount of DNA methylation in an analysis region that contains *K* CpG sites by using the mean methylation level (MML) $\mu$, given by^13^

$$\begin{aligned} \mu = \frac{1}{K} \sum _{k=1}^{K} \mu _k , \end{aligned}$$

where $\mu _k$ is the mean methylation at the *k*-th CpG site of the analysis region. The MML evaluates the fraction of CpG sites that are methylated in the analysis region, taking its minimum value when all CpG sites are unmethylated and achieving its maximum value when all CpG sites are methylated.

CpelNano also quantifies the amount of methylation stochasticity (pattern heterogeneity) in an analysis region by using the normalized methylation entropy (NME) *h*, an information-theoretic measure of stochasticity given by^13^

$$\begin{aligned} h = -\frac{1}{K} \sum _{\pmb {x}} g(\pmb {x}) \log _2 g(\pmb {x}), \end{aligned}$$

where $g(\pmb {x})$ is the probability distribution of the methylation state within the analysis region. The NME ranges between 0 and 1, taking its minimum value when a single methylation pattern is observed over the analysis region (perfectly ordered methylation) and achieving its maximum value when all methylation patterns are equally likely (fully disordered methylation).

Finally, to quantify differences between two probability distributions $g_1(\pmb {x})$ and $g_2(\pmb {x})$ of the methylation state in an analysis region corresponding to two conditions (e.g., normal/cancer), CpelNano employs the coefficient of methylation divergence (CMD) $d_{12}$, an information-theoretic criterion defined by

$$\begin{aligned} d_{12} := \frac{D(g_1 \, \Vert \, \overline{g}) + D(g_2 \, \Vert \, \overline{g})}{H(g_1 , \overline{g}) + H(g_2 , \overline{g})}. \end{aligned}$$

Here, $\overline{g}(\pmb {x})$ is a probability distribution of the methylation state in the analysis region associated with a CPEL model whose potential energy function is the average of the potential energy functions of the two CPEL models associated with the two conditions. Moreover,

$$\begin{aligned} D(f_1 \, \Vert \, f_2) = \sum _{\pmb {u}} f_1(\pmb {u}) \log _2 \frac{f_1(\pmb {u})}{f_2(\pmb {u})} \end{aligned}$$

is the Kullback-Leibler divergence between two probability distributions $f_1$ and $f_2$, and

$$\begin{aligned} H(f_1,f_2) = - \sum _{\pmb {u}} f_1(\pmb {u}) \log _2 f_2(\pmb {u}) \end{aligned}$$

is the cross-entropy between two random vectors with probability distributions $f_1$ and $f_2$. The CMD ranges between 0 and 1, taking its minimum value when the probability distributions $g_1$ and $g_2$ are identical and achieving its maximum when their supports do not overlap with the support of $\overline{g}$, indicating that $g_1$ is radically different from $g_2$.

### Hypothesis testing

To identify analysis regions exhibiting statistically significant DNA methylation discordance in the breast normal/cancer comparison, CpelNano was used to perform an unmatched sample pairs group comparison associated with a group of 5 breast normal nanopore samples and a second group associated with 5 breast cancer samples. CpelNano performed this task by using a “randomization model” that randomly assigned 5 out of the 10 samples to the first group and the remaining 5 samples to the second group, thus leading to 252 group assignments. A group permutation-based hypothesis testing method was then employed to test, for each analysis region, the null hypothesis that each pair of samples exhibited no methylation discordance regardless of their specific group assignment, achieving a $4.76\%$ false positive rate when a 0.05 significance level is used.

To that effect, CpelNano employed the following differential test statistics:

$$\begin{aligned} T_{\text {MML}}&= \frac{1}{5} \sum _{m=1}^{5} \mu _1^{(m)} - \frac{1}{5} \sum _{m=1}^{5} \mu _2^{(m)} \\ T_{\text {NME}}&= \frac{1}{5} \sum _{m=1}^{5} h_1^{(m)} - \frac{1}{5} \sum _{m=1}^{5} h_2^{(m)} \\ T_{\text {CMD}}&= \frac{1}{25} \sum _{m=1}^{5} \sum _{m'=1}^{5} d_{m m'}, \end{aligned}$$

where $\mu _1^{(m)}$, $h_1^{(m)}$ and $\mu _2^{(m)}$, $h_2^{(m)}$ are the MMLs and NMEs associated with the *m*-th sample in the first group and the *m*-th sample in the second group, and $d_{m m'}$ is the CMD obtained by comparing the probability distributions of methylation associated with the *m*-th sample of the first group and the $m'$-th sample of the second group. Notably, the test statistic $T_{\text {MML}}$ quantifies the difference between the average of the mean methylation levels in the first and second groups, $T_{\text {NME}}$ assesses the difference between the average of normalized methylation entropies, and $T_{\text {CMD}}$ quantifies the average of all observed differences between the probability distributions of methylation in the two groups. Finally, the Benjamini-Hochberg procedure for FDR control was applied and an analysis region was declared to be statistically significant if its corrected *P*-value (*Q*-value) was no larger than 0.05.

### Benchmarking model estimation

Performance evaluation of the EM-based maximum-likelihood approach employed by CpelNano for estimating the parameters of the CPEL model was performed using a simulation-based benchmarking scheme (Fig. S4). Four levels of nanopore noise were considered with standard deviations $\text {sd}=2,2.5,3,3.5$. For each standard deviation, the DeepSimulator^50,51^ was used to construct five sets of nanopore data with coverages $5$×, $10$×, $15$×, $20$×, and $25$× by means of an iterative procedure that considered only a portion of available nanopore reads. During the first iteration, one read was picked at random from the initial pool $\text {P}(0)$ of all available nanopore reads and two new pools $\text {P}(1)$ and $\text {P}'(1)$ were formed, with the first containing the read removed from $\text {P}(0)$ and the second containing the remaining reads. During the second iteration, one read was picked at random from $\text {P}'(1)$ and two new pools $\text {P}(2)$ and $\text {P}'(2)$ were generated, with the first containing all reads removed from $\text {P}(0)$ and the second containing the remaining reads. At each iteration *k*, the coverage at each CpG site was calculated as the number of reads in *P*(*k*) overlapping the CpG site, and subsequent iterations proceeded until the average of all CpG coverages in Chr. 22 was no less than the desired amount.

The reads in a nanopore data set with a given coverage were base-called and aligned to the reference genome using minmap2^52^ and subsequently used as input to Nanopolish^4^ to produce the information required for performing EM-based maximum-likelihood estimation of the $\alpha$, $\beta$, and $\gamma$ parameters of the CPEL model. This was performed in estimation regions that contained at least 10 CpG sites, had at least the desired average coverage per CG-group (a well-defined genomic region containing a cluster of CpG sites; see Supplementary Methods), and for which methylation information was available for at least 2/3 of their CG-groups. To assess parameter estimation performance in an estimation region, the closeness of estimated CPEL parameter values $\widehat{\alpha },\widehat{\beta }, \widehat{\gamma }$ to their “true” values $\alpha , \beta ,\gamma$ was evaluated by using cosine similarity as a measure, given by

$$\begin{aligned} s = \frac{\widehat{\alpha } \alpha + \widehat{\beta } \beta + \widehat{\gamma } \gamma }{\sqrt{(\widehat{\alpha }^2 + \widehat{\beta }^2+\widehat{\gamma }^2) (\alpha ^2+\beta ^2+\gamma ^2)}}. \end{aligned}$$

Notably, $-1 \le s \le 1$, with $s=-1$ implying maximum dissimilarity, $s=1$ implying maximum similarity, $s=0$ implying orthogonality or decorrelation, and in-between values indicating intermediate similarity or dissimilarity.

The closeness of the means ${\text {E}}[X_n]$ and pairwise correlations ${\text {E}}[X_n X_{n+1}]$ predicted by estimated CPEL models to their true values was also evaluated at each CpG site *n* of Chr. 22 by using the absolute error as a measure of goodness. This evaluation was also done for the means and pairwise correlations predicted by CPEL models estimated directly from the methylation calls made by Nanopolish^4^, as well as empirically. Notably, the computed absolute errors cannot exceed 1 since ${\text {E}}[X_n] = \Pr [X_n=1]$ and ${\text {E}}[X_nX_{n+1}] = \Pr [X_n=1, X_{n+1}=1]$, implying that $0 \le {\text {E}} [X_n], {\text{E}}[X_nX_{n+1}] \le 1$. Here, we set $X_n=0$ if the *n*-th CpG site is unmethylated and $X_n=1$ if the site is methylated.

### Data preprocessing and alignment

Quality control and adapter trimming of the raw WGBS data was performed using Trim Galore (v0.5.0; https://github.com/FelixKrueger/TrimGalore/). WGBS reads were aligned to the human reference assembly GRCh38.p12 (https://www.ncbi.nlm.nih.gov/assembly/GCF_000001405.38/) using minmap2, followed by removal of PCR duplicates using Bismark^53^ (v0.20.0). Basecalling of the current signals in the FAST5 nanopore files was performed using ONT’s Guppy (CPU mode) whereas alignment of the resulting nanopore reads to GRCh38.p12 was done using minimap2.

### Genomic features and regions

Files and tracks bear genomic coordinates for the human assembly GRCh38.p12. Annotations for CGIs were obtained from the University of California Santa Cruz (UCSC) (http://hgdownload.cse.ucsc.edu/goldenpath/hg38/database/cpgIslandExt.txt.gz). CGI shores were defined as sequences flanking 2-kb on either side of CGIs, CGI shelves as sequences flanking 2-kb beyond the shores, and open seas as everything else. Genes and TSSs were identified using the R package “TxDb.Hsapiens.UCSC.hg19.knownGene” and promoter regions of genes were taken to be the 4-kb window centered at their TSSs. Annotations for repetitive elements were obtained from UCSC by using the Table Browser functionality (http://genome.ucsc.edu/cgi-bin/hgTables/) and by choosing group “Repeats” and track “RepeatMasker”.

## Supplementary Information

Supplementary Information.

## Data Availability

This study used four publicly available datasets. The GSM2308632 WGBS data (Illumina HiSeq 2500, coverage $\sim \!\! 100$×) and the NA12878 nanopore data (MinION, coverage $\sim \! 30$×) identified with the human GM12878 Utah/Ceph lymphoblastoid cell line, were respectively obtained from ENCODE^54^ (https://www.ncbi.nlm.nih.gov/sra/SRX2157047/) and Jain et al.^8^ (https://github.com/nanopore-wgs-consortium/NA12878/). The MCF-10A nanopore data (MinION, $271$× median average coverage over 10 CpG sites) identified with the human breast normal epithelial cell line MCF-10A, and the MDA-MB-231 nanopore data (MinION, $249$× median average coverage over 10 CpG sites) identified with the human breast cancer epithelial cell line MDA-MB-231, were acquired from the original paper^29^ (https://www.ncbi.nlm.nih.gov/bioproject/?term=PRJNA531320/). In addition, all necessary unpublished data for replicating the results, including data obtained from the simulations, can be downloaded from http://www.cis.jhu.edu/~goutsias/data/paper-results.zip.

## Code availability

The method presented in this paper has been implemented in a Julia package called CpelNano. The source code and associated instructions can be downloaded from https://github.com/jordiabante/CpelNano.jl/.
