# Supplementary material for: Estimating DNA methylation potential energy landscapes from nanopore sequencing data
Source: Sci Rep. 2021 Nov 3;11:21619. doi: 10.1038/s41598-021-00781-x (PMC8566571; doi:10.1038/s41598-021-00781-x)
Supplement: Supplementary file 1 — Supplementary Information. [file 41598_2021_781_MOESM1_ESM.pdf]

SUPPLEMENTARY INFORMATION

Estimating DNA methylation potential energy landscapes from nanopore sequencing data

Jordi Abante[1,2,3*], Sandeep Kambhampati[4,5], Andrew P. Feinberg[4,6,7] and John Goutsias[1,2*]

---

[1]Whitaker Biomedical Engineering Institute, Johns Hopkins University, Baltimore, MD 21218, USA. [2]Department of Electrical & Computer Engineering, Johns Hopkins University, Baltimore, MD 21218, USA. [3]Present address: Department of Biomedical Data Science, Stanford University School of Medicine, Stanford, CA 94305, USA. [4]Department of Biomedical Engineering, Johns Hopkins University, Baltimore, MD 21205, USA. [5]Present address: Department of Biomedical Informatics, Harvard Medical School, Boston, MA 02115, USA. [6]Center for Epigenetics, Johns Hopkins University School of Medicine, Baltimore, MD 21205, USA. [7]Department of Medicine, Johns Hopkins University School of Medicine, Baltimore, MD 21205, USA.

[*]email: jabante@stanford.edu or goutsias@jhu.edu

Supplementary Methods

1. Modeling DNA methylation

For reasons to become clear in the next section, CpelNano follows Nanopolish[1] and clusters the CpG sites along the reference genome into groups, so that the last C nucleotide of a group and the first C nucleotide of the next group are separated by at least 10 bases. It then forms the base sequence between the first C nucleotide and the last G nucleotide in each group and extends each sequence by 5 bases upstream and 4 bases downstream (Fig. 1a). This process defines DNA segments $\mathcal{G}_l$, $l = 1, 2, \ldots$, along the genome, which we refer to as *CG-groups*, that include at least one CpG site. We found that 84.1% (18,770,638) of the CG-groups in the human reference genome (autosomal chromosomes, built GRCh38.p12) contain 11 bases, and therefore include 1 CpG site, whereas the remaining 15.9% (3,549,239) of the CG-groups contain from 12 to 1,002 bases and include 2 to 212 CpG sites.

CpelNano also partitions each chromosome into non-overlapping genomic regions $\mathcal{R}_k$, $k = 1, 2, \ldots$, which we refer to as *estimation regions*, using the following recursive scheme (Fig. 1b). For $k = 0, 1, \ldots$, a 3-kb window is placed along the genome starting at the first nucleotide after the most downstream nucleotide in $\mathcal{R}_k$ (when $k = 0$, the starting nucleotide is taken to be the first nucleotide in the chromosome). This window defines the estimation region $\mathcal{R}_{k+1}$, provided that its most downstream nucleotide does not intersect a CG-group. Note, however, that if this is not true and if the window contains more than 50% of an intersecting CG-group, its size is increased to form the smallest region $\mathcal{R}_{k+1}$ that fully contains the intersecting CG-group. Otherwise, the window is decreased to form the largest region $\mathcal{R}_{k+1}$ whose downstream nucleotide does not intersect a CG-group. We found 66 estimation regions in the human genome (of size between 2,994 and 3,000 bases) containing 1 CG-group, which include 1 or 2 CpG sites each, and 919,255 estimation regions (of size between 2,905 and 3,071 bases) containing at least 2 CG-groups, which include from 2 to 487 CpG sites each.

Within an estimation region $\mathcal{R}$ that contains N CpG sites $n = 1, 2, \ldots, N$, CpelNano characterizes the true state of DNA methylation using the $N \times 1$ random state vector $\boldsymbol{X} = [X_1 \; X_2 \; \cdots \; X_N]^T$, where $X_n = 0$, if the n-th CpG site is unmethylated, and $X_n = 1$, if the CpG site is methylated. As a consequence of the well-known maximum-entropy principle[2], the probability distribution of methylation that is consistent with methylation means and pairwise correlations at each CpG site is given by

$$p(\boldsymbol{x}) := \Pr[\boldsymbol{X} = \boldsymbol{x}] = \frac{1}{\zeta} \exp\{-U(\boldsymbol{x})\}, \text{ for every } \boldsymbol{x} \in \mathcal{X}, \quad (1)$$

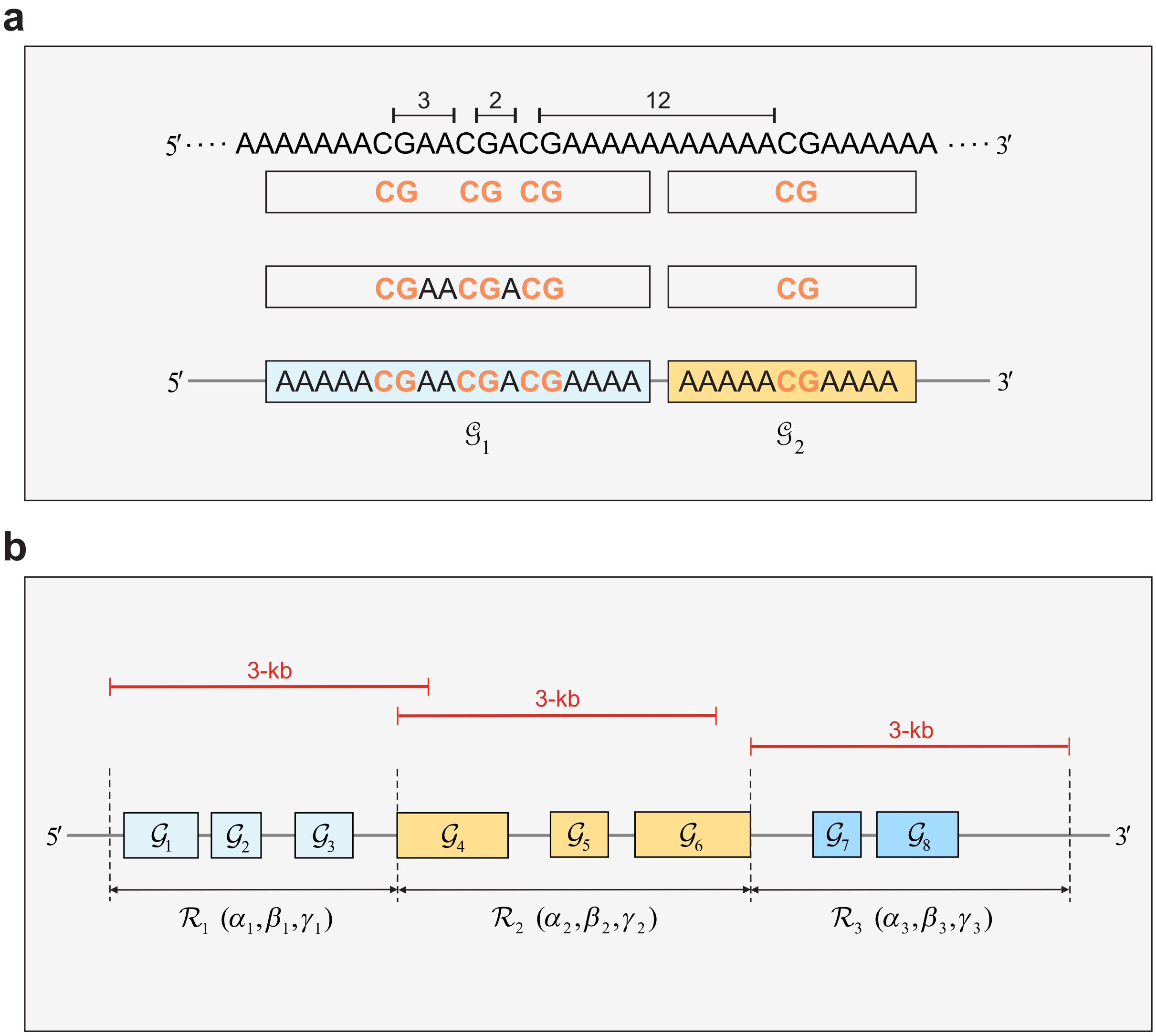

Figure 1. CG-groups and estimation regions. (**a**) Example of clustering CpG sites along the genome into groups of CG dinucleotides that are separated by at least 10 bases. The CG-groups $\mathcal{G}_1$ and $\mathcal{G}_2$ are formed from two CG clusters by inserting the bases between the first C nucleotide and the last G nucleotide in each cluster and by extending each sequence by 5 bases upstream and 4 bases downstream. (**b**) Example of partitioning a given chromosome into estimation regions. Estimation region $\mathcal{R}_1$ is less than 3,000 bases long, whereas $\mathcal{R}_2$ is more than 3,000 bases long. This guarantees that each estimation region fully contains CG-groups. Note that the CPEL model given by Eqs. (7)-(9) is associated with different parameters α, β, and γ within each estimation region, whose values must be estimated from nanopore data corresponding to the specific region.

where $\mathcal{X}$ is the set of all 2^N possible methylation patterns associated with $\mathcal{R}$,

$$U(\boldsymbol{x}) = -\sum_{n=1}^{N} a_n(2x_n - 1) - \sum_{n=1}^{N-1} b_n(2x_n - 1)(2x_{n+1} - 1) \tag{2}$$

is the potential energy function of $\boldsymbol{X}$, a_n and b_n are two parameters associated with the n-th CpG site, and

$$\zeta = \sum_{\boldsymbol{x} \in \mathcal{X}} \exp\{-U(\boldsymbol{x})\} \tag{3}$$

is a normalizing constant known as the partition function. Note that parameter a_n affects the propensity of the n-th CpG site to be methylated without the influence of nearby CpG sites, whereas parameter b_n accounts for the possibility that the methylation states of two contiguous CpG sites n and $n+1$ would be correlated.

The previous probability distribution generalizes the classical one-dimensional Ising model of statistical physics[3] by including "external field" parameters a_n, $n = 1, 2, \ldots, N$, and "interaction" parameters b_n, $n = 1, 2, \ldots, N-1$, which are not necessarily constant. Note however that this distribution does not account for evidence suggesting that the likelihood of a given CpG site to be methylated depends strongly on the fraction of CpG sites in a local neighborhood, as well as on the methylation status of nearby CpG sites whose influence diminishes as their nucleotide distance from the given CpG site increases[4,5]. To address this issue, CpelNano follows a previous approach by Jenkinson et al.[6,7] and sets

$$a_n = \alpha + \beta\rho_n \quad \text{and} \quad c_n = \frac{\gamma}{d_n}, \tag{4}$$

where α, β, and γ are parameters characteristic to the estimation region $\mathcal{R}$, ρ_n is the CpG density, defined as the fraction of dinucleotides that are CpG sites in a symmetric neighborhood of 1,000 nucleotides centered at the n-th CpG site, given by

$$\rho_n = \frac{1}{1{,}000} \times [\text{\# of CpG sites within } \pm 500 \text{ nucleotides downstream and upstream of } n], \tag{5}$$

and d_n is the distance of the n-th CpG site from its downstream CpG site $n+1$, given by

$$d_n = [\text{\# of base-pair steps between the cytosines of CpG sites } n \text{ and } n+1]. \tag{6}$$

Notably, parameter α accounts for intrinsic factors that affect the propensity of CpG sites to be methylated, whereas parameters β and γ modulate the influence of CpG density and distance on methylation, respectively, which we assume here to be applied uniformly on all CpG sites in each estimation region $\mathcal{R}$.

As a consequence of Eqs. (1)-(6), CpelNano characterizes the true methylation vector $\boldsymbol{X}$ over an estimation region $\mathcal{R}$ that contains N CpG sites $n = 1, 2, \ldots, N$ using the probability distribution

$$p(\boldsymbol{x};\boldsymbol{\theta}) = \frac{1}{\zeta(\boldsymbol{\theta})} \exp\left\{-U(\boldsymbol{x};\boldsymbol{\theta})\right\}, \text{ for every } \boldsymbol{x} \in \mathcal{X}, \tag{7}$$

with potential energy function

$$U(\boldsymbol{x};\boldsymbol{\theta}) = -\alpha \sum_{n=1}^{N} (2x_n - 1) - \beta \sum_{n=1}^{N} \rho_n (2x_n - 1) - \gamma \sum_{n=1}^{N-1} (2x_n - 1)(2x_{n+1} - 1)/d_n \tag{8}$$

and partition function

$$\zeta(\boldsymbol{\theta}) = \sum_{\boldsymbol{x}\in\mathcal{X}} \exp\{-U(\boldsymbol{x};\boldsymbol{\theta})\}, \tag{9}$$

where $\boldsymbol{\theta} = [\alpha\ \beta\ \gamma]^T$ is the vector of the underlying parameters associated with region $\mathcal{R}$. We refer to this probability distribution as the correlated potential energy landscape (CPEL) model. This is an exponential family of distributions with three sufficient statistics[8],

$$S_1(\boldsymbol{X}) := \sum_{n=1}^{N} (2X_n - 1), \tag{10}$$

$$S_2(\boldsymbol{X}) := \sum_{n=1}^{N} \rho_n (2X_n - 1), \tag{11}$$

and

$$S_3(\boldsymbol{X}) := \sum_{n=1}^{N-1} (2X_n - 1)(2X_{n+1} - 1)/d_n, \tag{12}$$

each summarizing all information available in a given data sample about the values of the model parameters α, β, and γ, respectively, in terms of the methylation states $2X_n - 1$ at individual CpG sites and the methylation co-occurrences $(2X_n - 1)(2X_{n+1} - 1)$ at pairs of consecutive CpG sites.

Notably, the CPEL model summarizes the common understanding that methylation of a CpG site depends on two distinct factors: the local CpG architecture, specified by CpG densities and distances, as well as the biochemical environment provided by the methylation machinery, quantified by parameters α, β, and γ. Moreover, Eqs. (7)-(9) lead to a form of the classical Ising model of statistical physics that has been successful in predicting the probability of DNA methylation over regions of the genome from CpG density and distance alone[6]. However, additional sequence-specific factors may influence DNA methylation[9,10], which could be included in future versions of the model if necessary.

2. Maximum-likelihood parameter estimation

Use of the CPEL model for methylation analysis of nanopore sequencing data requires estimation of its parameters $\boldsymbol{\theta}$. Performing this task requires availability of a set $\boldsymbol{y}$ of nanopore data that contain sufficient information about the true methylation state $\boldsymbol{x}$ over an estimation region $\mathcal{R}$. Given such observations, a value $\widehat{\boldsymbol{\theta}}$ can then be found that maximizes the likelihood that the observed nanopore data $\boldsymbol{y}$ have been generated by the CPEL model with parameters $\widehat{\boldsymbol{\theta}}$ by solving the following optimization problem:

$$\widehat{\boldsymbol{\theta}} = \arg\max_{\boldsymbol{\theta}} f(\boldsymbol{y};\boldsymbol{\theta}) = \arg\max_{\boldsymbol{\theta}} \sum_{\boldsymbol{x}\in\mathcal{X}} q(\boldsymbol{y} \mid \boldsymbol{x}) p(\boldsymbol{x};\boldsymbol{\theta}). \tag{13}$$

In this equation, $q(\boldsymbol{y} \mid \boldsymbol{x})$ is the conditional probability distribution of observed nanopore data $\boldsymbol{Y}$, given that the true methylation state in $\mathcal{R}$ is $\boldsymbol{x}$ (also known as emission probabilities), and $f(\boldsymbol{y};\boldsymbol{\theta})$ is the marginal probability distribution of $\boldsymbol{Y}$ when the parameter values of the generating CPEL model are given by $\boldsymbol{\theta}$. Notably, both p and q depend on the genetic context, but for notational convenience, we do not explicitly denote this dependence here.

CpelNano uses observations $\boldsymbol{Y}$ obtained through Nanopolish[1]. Although Nanopolish[1] has been developed for detecting 5mC methylation, it does so by finding abrupt changes in the nanopore current signals during sequencing, which define "events" of relatively stationary behavior indicating discrete motion of the DNA sequence through the nanopore[1,11]. Moreover, and for a given genomic region $\mathcal{R}$ that fully contains a set $\{\mathcal{G}_l,\, l = 1, 2, \ldots, L\}$ of CG-groups, it evaluates the conditional probabilities (emission probabilities) $q_l(\boldsymbol{y}_l \mid \boldsymbol{x}_l)$ of the vector $\boldsymbol{Y}_l$ of all signal values mapped to a CG-group $\mathcal{G}_l(\boldsymbol{x}_l)$ whose CpG sites are methylated or unmethylated in accordance to the corresponding methylation state $\boldsymbol{x}_l$. By setting $\boldsymbol{Y} = [\boldsymbol{Y}_1\, \boldsymbol{Y}_2\, \cdots\, \boldsymbol{Y}_L]^T$ and by making the reasonable assumption that, given the methylation state $\boldsymbol{x}$ in $\mathcal{R}$, the signal values associated with individual CG-groups $\mathcal{G}_l$, $l = 1, 2, \ldots, L$, are conditionally independent, we obtain

$$q(\boldsymbol{y} \mid \boldsymbol{x}) = \prod_{l=1}^{L} q_l(\boldsymbol{y}_l \mid \boldsymbol{x}_l), \tag{14}$$

which is employed by CpelNano to compute the emission probabilities $q(\boldsymbol{y}|\boldsymbol{x})$ using Nanopolish[1].

Unfortunately, the current version of Nanopolish[1] has been trained to compute the emission probabilities $q_l(\boldsymbol{y}_l \mid \boldsymbol{x}_l)$ only when the values of $\boldsymbol{x}_l$ are all ones (fully methylated state) or all zeros (fully unmethylated sate). To take this issue into account, note that

$$f(\boldsymbol{y};\boldsymbol{\theta}) = \sum_{\boldsymbol{x}\in\mathcal{X}} q(\boldsymbol{y} \mid \boldsymbol{x}) p(\boldsymbol{x};\boldsymbol{\theta}) = \sum_{\boldsymbol{x}\in\bar{\mathcal{X}}} q(\boldsymbol{y} \mid \boldsymbol{x}) p(\boldsymbol{x};\boldsymbol{\theta}) + \sum_{\boldsymbol{x}\in\mathcal{X}\setminus\bar{\mathcal{X}}} q(\boldsymbol{y} \mid \boldsymbol{x}) p(\boldsymbol{x};\boldsymbol{\theta}), \tag{15}$$

where $\bar{\mathcal{X}}$ is the set of all methylation states in $\mathcal{X}$ for which the CpG sites within the CG-groups in $\mathcal{R}$ are all methylated or unmethylated, and $\mathcal{X} \setminus \bar{\mathcal{X}}$ are the remaining states. Then, by considering evidence that DNA methylation at CpG sites that are closely clustered to each other are most often strongly correlated[4,5], we can assume that

$$p(\boldsymbol{x};\boldsymbol{\theta}) \simeq 0, \quad \text{for every } \boldsymbol{x} \in \mathcal{X} \setminus \bar{\mathcal{X}}, \tag{16}$$

in which case we can approximately set

$$f(\boldsymbol{y};\boldsymbol{\theta}) = \sum_{\boldsymbol{x} \in \bar{\mathcal{X}}} q(\boldsymbol{y} \mid \boldsymbol{x}) \bar{p}(\boldsymbol{x};\boldsymbol{\theta}), \tag{17}$$

where

$$\bar{p}(\boldsymbol{x};\boldsymbol{\theta}) = \begin{cases} p(\boldsymbol{x};\boldsymbol{\theta}), & \text{for } \boldsymbol{x} \in \bar{\mathcal{X}} \\ 0, & \text{for } \boldsymbol{x} \in \mathcal{X} \setminus \bar{\mathcal{X}}. \end{cases} \tag{18}$$

This implies that

$$\bar{p}(\boldsymbol{x};\boldsymbol{\theta}) = \frac{1}{\bar{\zeta}(\boldsymbol{\theta})} \exp\left\{-\bar{U}(\boldsymbol{x};\boldsymbol{\theta})\right\}, \text{ for every } \boldsymbol{x} \in \bar{\mathcal{X}}, \tag{19}$$

where

$$\bar{U}(\boldsymbol{x};\boldsymbol{\theta}) = \begin{cases} U(\boldsymbol{x};\boldsymbol{\theta}), & \text{for } \boldsymbol{x} \in \bar{\mathcal{X}} \\ \infty, & \text{for } \boldsymbol{x} \in \mathcal{X} \setminus \bar{\mathcal{X}}, \end{cases} \tag{20}$$

and

$$\bar{\zeta}(\boldsymbol{\theta}) = \sum_{\boldsymbol{x} \in \bar{\mathcal{X}}} \exp\{-\bar{U}(\boldsymbol{x};\boldsymbol{\theta})\}. \tag{21}$$

Notably, $\bar{p}(\boldsymbol{x};\boldsymbol{\theta}) \rightarrow p(\boldsymbol{x};\boldsymbol{\theta})$ as $\bar{\mathcal{X}} \rightarrow \mathcal{X}$, which shows that the previous approximation can be eliminated by a better training of Nanopolish[1].

With each CG-group $\mathcal{G}_l$, we can now associate the random variable

$$Z_l = \begin{cases} 1, & \text{if } X_n = 1 \text{ at every CpG site } n \in \mathcal{G}_l \\ 0, & \text{if } X_n = 0 \text{ at every CpG site } n \in \mathcal{G}_l, \end{cases} \tag{22}$$

which we refer to as the *methylation index* of the CG-group, since its value indicates whether the CG-group is fully methylated or fully unmethylated. It can be shown from Eqs. (8), (19), (20), & (22) that, if $\boldsymbol{Z} = [Z_1\, Z_2\, \cdots\, Z_L]^T$ is the random vector of methylation indices associated with the CG-groups in an estimation region $\mathcal{R}$, then its probability distribution $\pi(\boldsymbol{z};\boldsymbol{\theta}) := \Pr[\boldsymbol{Z} = \boldsymbol{z};\boldsymbol{\theta}]$ is given by

$$\pi(\boldsymbol{z};\boldsymbol{\theta}) = \frac{1}{\zeta(\boldsymbol{\theta})} \exp\left\{-V(\boldsymbol{z};\boldsymbol{\theta})\right\}, \text{ for every } \boldsymbol{z} \in \mathcal{Z}, \tag{23}$$

with potential energy function

$$V(\boldsymbol{z};\boldsymbol{\theta}) = -\alpha \sum_{l=1}^{L} N_l(2z_l - 1) - \beta \sum_{l=1}^{L} \bar{\rho}_l N_l(2z_l - 1) - \gamma \sum_{l=1}^{L-1} (2z_l - 1)(2z_{l+1} - 1)/\bar{d}_l \quad (24)$$

and partition function

$$\zeta(\boldsymbol{\theta}) = \sum_{\boldsymbol{z}\in\mathcal{Z}} \exp\{-V(\boldsymbol{z};\boldsymbol{\theta})\}, \quad (25)$$

where $\mathcal{Z}$ is the set of all possible 2^L methylation index values in $\mathcal{R}$. In these equations, which we refer to as the *reduced* CPEL model,

$$\bar{\rho}_l = \frac{1}{N_l} \sum_{n\in\mathcal{N}_l} \rho_n \quad (26)$$

is the average CpG density within the l-th CG-group $\mathcal{G}_l$ containing N_l CpG sites in $\mathcal{N}_l$, and $\bar{d}_l$ is the distance between the last CpG site in the CG-group $\mathcal{G}_l$ and the first CpG site in the CG-group $\mathcal{G}_{l+1}$.

Although the reduced CPEL model always depends on parameters α and β, it also depends on the interaction parameter γ, provided that $\mathcal{R}$ contains at least two CG-groups. In this case, the original CPEL model can be estimated from available data obtained by Nanopolish[1] by fitting the reduced CPEL model to that data. For this reason, CpelNano does not model regions $\mathcal{R}$ that contain only one CG-group, which are nevertheless very few (we found only 66 such regions in the human genome) and insignificant (each contains only 1 or 2 CpG sites). In addition, and for reliable parameter estimation, it only models regions that contain at least 10 CpG sites, with average coverage of at least $5\times$ per CG-group, and for which methylation information is available for at least $2/3$ of their CG-groups.

Note now that, every methylation pattern $\boldsymbol{x} \in \bar{\mathcal{X}}$ can be generated by a *unique* vector of methylation indices $\boldsymbol{z} \in \mathcal{Z}$. Consequently, if $\boldsymbol{x} = \boldsymbol{s}(\boldsymbol{z})$ is the methylation pattern in $\bar{\mathcal{X}}$ associated with $\boldsymbol{z}$, then Eq. (17) implies that

$$\begin{aligned} f(\boldsymbol{y};\boldsymbol{\theta}) &= \sum_{\boldsymbol{x}\in\bar{\mathcal{X}}} q(\boldsymbol{y} \mid \boldsymbol{x})\bar{p}(\boldsymbol{x};\boldsymbol{\theta}) \\ &= \sum_{\boldsymbol{z}\in\mathcal{Z}} q(\boldsymbol{y} \mid \boldsymbol{s}(\boldsymbol{z}))\bar{p}(\boldsymbol{s}(\boldsymbol{z});\boldsymbol{\theta}) \\ &= \sum_{\boldsymbol{z}\in\mathcal{Z}} q(\boldsymbol{y} \mid \boldsymbol{s}(\boldsymbol{z}))\pi(\boldsymbol{z};\boldsymbol{\theta}), \end{aligned} \quad (27)$$

by virtue of the fact that $\pi(\boldsymbol{z};\boldsymbol{\theta}) = \bar{p}(\boldsymbol{s}(\boldsymbol{z});\boldsymbol{\theta})$. This result, together with Eqs. (13) & (14), leads to the following maximum-likelihood parameter estimation problem:

$$\begin{aligned}\widehat{\boldsymbol{\theta}} &= \arg\max_{\boldsymbol{\theta}} f(\boldsymbol{y};\boldsymbol{\theta}) \\ &= \arg\max_{\boldsymbol{\theta}} \sum_{\boldsymbol{z}\in\mathcal{Z}} \pi(\boldsymbol{z};\boldsymbol{\theta}) q(\boldsymbol{y} \mid \boldsymbol{s}(\boldsymbol{z})) \\ &= \arg\max_{\boldsymbol{\theta}} \sum_{\boldsymbol{z}\in\mathcal{Z}} \pi(\boldsymbol{z};\boldsymbol{\theta}) \prod_{l=1}^{L} q_l(\boldsymbol{y}_l \mid \boldsymbol{s}_l(z_l)),\end{aligned} \tag{28}$$

where $\boldsymbol{s}_l(z_l)$ is either the fully methylated pattern within $\mathcal{G}_l$ (when $z_l = 1$) or the fully unmethylated pattern (when $z_l = 0$). By solving this problem, CpelNano facilitates estimation of the parameters of the original CPEL model via maximum likelihood. We summarize the main steps of this approach with an example in Fig. 2.

REMARKS:

1. We show in Section 4 below that, when the true methylation state $\boldsymbol{X}$ over a genomic region $\mathcal{R}$ is modeled via a CPEL model, it forms a (non-homogeneous) Markov chain. As a consequence, CpelNano addresses the statistical challenge of nanopore noise by employing a data-generative hidden Markov model (HMM) approach. This approach considers the fact that the true Markovian methylation state $\boldsymbol{X}$ cannot be directly observed by nanopore sequencing (i.e., it is a "hidden" state) but only indirectly through an observable state $\boldsymbol{Y}$ of average nanopore current values, which is conditionally specified by using the emission probabilities $q_l(\boldsymbol{y}_l \mid \boldsymbol{s}_l(z_l))$ computed from Nanopolish[1]. The first objective of CpelNano is to learn the CPEL model of $\boldsymbol{X}$ by observing $\boldsymbol{Y}$.

2. Although a number of artificial neural network approaches have been recently proposed in the literature for detecting 5mC methylation using nanopore sequencing, including DeepMod[12] and DeepSignal[13], only Nanopolish[1] can be used to compute the emission probabilities required for inferring a presumed stochastic DNA methylation model from nanopore data. This is due to the fact that neural network approaches only address the *inverse problem* of learning the probabilities of methylation at individual CpG sites from nanopore data. Although Nanopolish[1] has been designed to perform the same task, it does so by also addressing the *forward problem* of computing the probabilities of observed nanopore data generated by a given input methylation sequence.

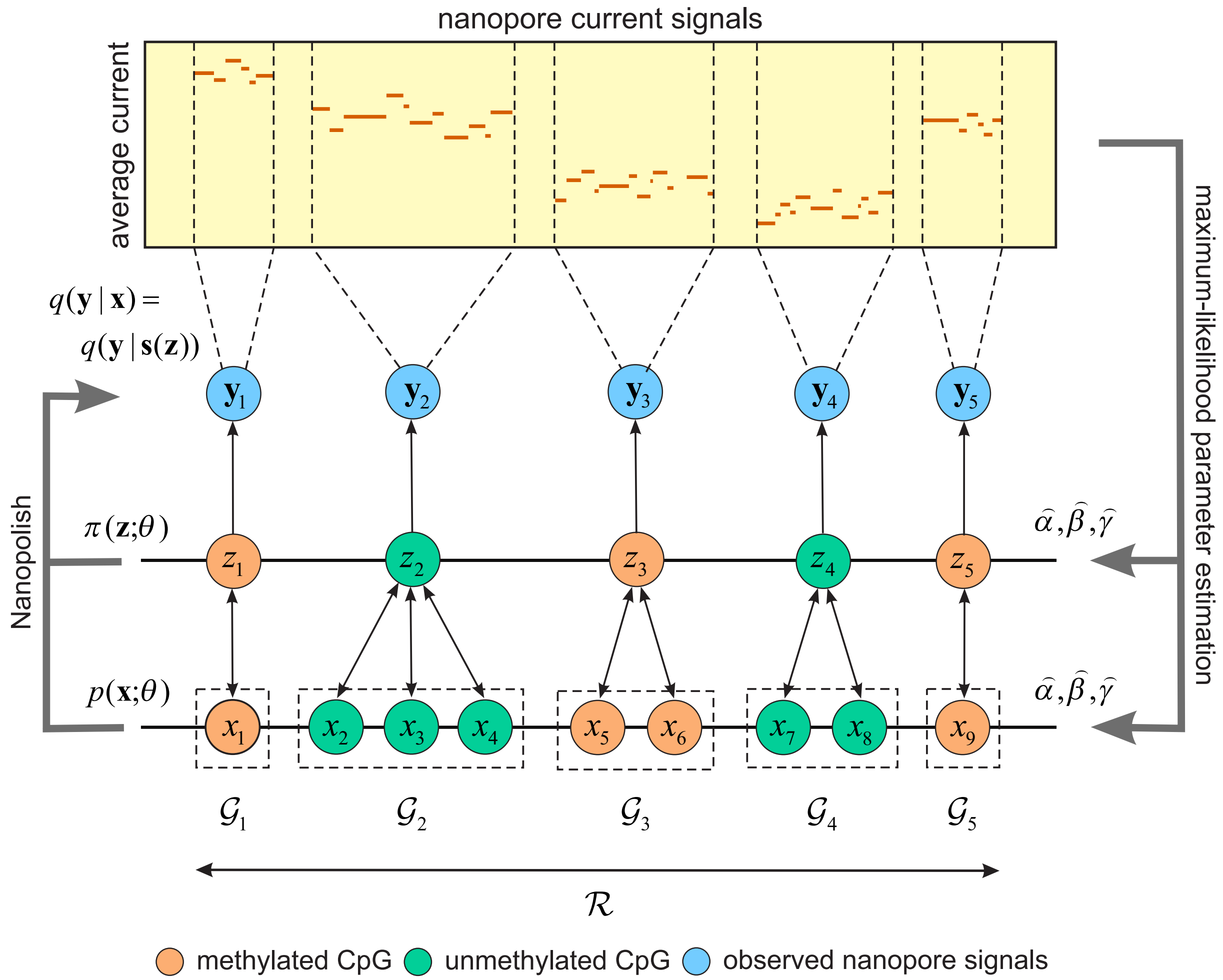

Figure 2. Maximum-likelihood estimation of the CPEL model. Within an estimation region $\mathcal{R}$ that fully contains five CG-groups $\mathcal{G}_1$, $\mathcal{G}_2$, $\mathcal{G}_3$, $\mathcal{G}_4$, and $\mathcal{G}_5$, CpelNano estimates the parameters α, β, and γ of the CPEL model $p(\boldsymbol{x};\boldsymbol{\theta})$ by maximizing the likelihood that observed average current events $\boldsymbol{y} = \{\boldsymbol{y}_1, \boldsymbol{y}_2, \boldsymbol{y}_3, \boldsymbol{y}_4, \boldsymbol{y}_5\}$ (blue) have been generated by the reduced CPEL model $\pi(\boldsymbol{z};\boldsymbol{\theta})$, where $\boldsymbol{z} = \{z_1, z_2, z_3, z_4, z_5\}$ are the methylation indices associated with the CG-groups. This requires knowledge of the emission probabilities $q(\boldsymbol{y} \mid \boldsymbol{x}) = q(\boldsymbol{y} \mid \boldsymbol{s}(\boldsymbol{z}))$ of the observed nanopore current signals given the methylation state $\boldsymbol{x} = \boldsymbol{s}(\boldsymbol{z})$ within $\mathcal{R}$ in which all CpG sites inside each CG-group are either methylated (orange) or unmethylated (green), which are computed by Nanopolish[1].

3. Gigante et al.[14] have proposed a method for estimating the mean methylation level at CpG sites within a CG-group $\mathcal{G}_l$ using Nanopolish[1]. They did so by considering the fact that

$$\frac{\Pr[\boldsymbol{X}_l = \boldsymbol{s}_l(1) \mid \boldsymbol{Y}_l = \boldsymbol{y}_l^{(m)}]}{\Pr[\boldsymbol{X}_l = \boldsymbol{s}_l(0) \mid \boldsymbol{Y}_l = \boldsymbol{y}_l^{(m)}]} = \frac{\Pr[\boldsymbol{Y}_l = \boldsymbol{y}_l^{(m)} \mid \boldsymbol{X}_l = \boldsymbol{s}_l(1)]\,\Pr[\boldsymbol{X}_l = \boldsymbol{s}_l(1)]}{\Pr[\boldsymbol{Y}_l = \boldsymbol{y}_l^{(m)} \mid \boldsymbol{X}_l = \boldsymbol{s}_l(0)]\,\Pr[\boldsymbol{X}_l = \boldsymbol{s}_l(0)]} \tag{29}$$

in our notation, where $\boldsymbol{y}_l^{(m)}$, $m = 1, 2, \ldots, M$, are multiple independent nanopore reads associated with $\mathcal{G}_l$. By setting

$$\Pr[\boldsymbol{X}_l = \boldsymbol{s}_l(0) \mid \boldsymbol{Y}_l = \boldsymbol{y}_l] \simeq 1 - \Pr[\boldsymbol{X}_l = \boldsymbol{s}_l(1) \mid \boldsymbol{Y}_l = \boldsymbol{y}_l], \tag{30}$$

which can be justified by assuming that, given a nanopore read $\boldsymbol{y}_l$, the CpG sites within the CG-group $\mathcal{G}_l$ can approximately be only fully methylated or fully unmethylated [which is related to our Eq. (16)], it can be shown that

$$\Pr[\boldsymbol{X}_l = \boldsymbol{s}_l(0)] \simeq 1 - \Pr[\boldsymbol{X}_l = \boldsymbol{s}_l(1)]. \tag{31}$$

Then, Eqs. (29)-(31) lead to

$$\mu_l^{(m)} := \Pr[\boldsymbol{X}_l = \boldsymbol{s}_l(1) \mid \boldsymbol{Y}_l = \boldsymbol{y}_l^{(m)}] \simeq \left[1 + \frac{1-\pi}{\pi}\frac{q(\boldsymbol{y}_l^{(m)} \mid \boldsymbol{s}_l(0))}{q(\boldsymbol{y}_l^{(m)} \mid \boldsymbol{s}_l(1))}\right]^{-1}, \tag{32}$$

where $\pi := \Pr[\boldsymbol{X}_l = \boldsymbol{s}_l(1)]$ is the probability of the CG group $\mathcal{G}_l$ to be fully methylated, and $q(\boldsymbol{y}_l^{(m)} \mid \boldsymbol{s}_l(z)) = \Pr[\boldsymbol{Y}_l = \boldsymbol{y}_l^{(m)} \mid \boldsymbol{X}_l = \boldsymbol{s}_l(z)]$, for $z = 0, 1$, which is computed by Nanopolish[1]. Notably,

$$\Pr[\boldsymbol{X}_l = \boldsymbol{s}_l(1) \mid \boldsymbol{Y}_l = \boldsymbol{y}_l^{(m)}] \simeq \Pr[X_n = 1 \mid \boldsymbol{Y}_l = \boldsymbol{y}_l^{(m)}], \ \text{ for every } n \in \mathcal{G}_l, \tag{33}$$

due to Eq. (30). Therefore, and as a consequence of Eqs. (32) & (33), $\mu_l^{(m)}$ approximately provides the methylation mean at any CpG site $n \in \mathcal{G}_l$. Finally, and by making the (reasonable) assumption that methylation reads are randomly generated by the sequencer, the methylation mean at any CpG site in $\mathcal{G}_l$ is approximately computed by

$$\mu_n = \frac{1}{M}\sum_{m=1}^{M} \mu_l^{(m)}, \ \text{ for every } n \in \mathcal{G}_l, \tag{34}$$

where $\mu_l^{(m)}$ is calculated from Eq. (32) by setting $\pi = 1/2$ for each m. We should note, however, that this approach has several drawbacks: (i) a value must be assumed for π which must be the same for all CG-groups in the genome; (ii) computing different means at each CpG site inside a CG-group is not possible, unless Nanopolish[1] is better trained to facilitate such a feature (this is not an issue with CpelNano); (iii) computation of higher order methylation statistics, such as pairwise correlations, entropies, and probability distributions, is not possible.

3. Parameter estimation using the EM algorithm

The previous parameter estimation approach must be modified in order to consider the availability of multiple independent nanopore reads $\boldsymbol{y}_l^{(m)}$, $m = 1, 2, \ldots, M$, associated with each CG-group $\mathcal{G}_l$. Therefore, CpelNano is designed to solve the following maximum-likelihood problem:

$$\widehat{\boldsymbol{\theta}} = \arg\max_{\boldsymbol{\theta}} \prod_{m=1}^{M} \sum_{\boldsymbol{z} \in \mathcal{Z}} \pi(\boldsymbol{z}; \boldsymbol{\theta}) q(\boldsymbol{y}^{(m)} \mid \boldsymbol{z}), \tag{35}$$

with

$$q(\boldsymbol{y}^{(m)} \mid \boldsymbol{z}) = \prod_{l=1}^{L} \left[q_l(\boldsymbol{y}_l^{(m)} \mid \boldsymbol{s}_l(z_l)) \right]^{w_l^{(m)}}, \tag{36}$$

where $w_l^{(m)} = 1$, if the m-th observation is present in $\mathcal{G}_l$, and $w_l^{(m)} = 0$, if this observation is missing. Notably, and due to the required summation over all possible methylation indices $\boldsymbol{z}$ within an estimation region $\mathcal{R}$, evaluating the likelihood function in Eq. (35) is not computationally feasible when $\mathcal{R}$ contains many CG-groups and, therefore, directly performing maximum-likelihood parameter estimation using this equation is not appropriate. However, CpelNano addresses this issue by employing the expectation-maximization (EM) algorithm which results in iteratively applying the following two steps:

Expectation step: Given nanopore data $\boldsymbol{y} = \{\boldsymbol{y}^{(1)}, \boldsymbol{y}^{(2)}, \ldots, \boldsymbol{y}^{(M)}\}$ and a currently estimated value $\widehat{\boldsymbol{\theta}}_{i-1}$ of the parameters $\boldsymbol{\theta}$, the conditional expected value of the logarithm of the likelihood function $p(\boldsymbol{y}, \boldsymbol{Z})$ with respect to the methylation index vector $\boldsymbol{Z}$ is computed by

$$J(\boldsymbol{\theta}; \widehat{\boldsymbol{\theta}}_{i-1}) = \sum_{m=1}^{M} \sum_{\boldsymbol{z} \in \mathcal{Z}} \pi(\boldsymbol{z} \mid \boldsymbol{y}^{(m)}; \widehat{\boldsymbol{\theta}}_{i-1}) \Big[\ln \pi(\boldsymbol{z}; \boldsymbol{\theta}) + \sum_{l=1}^{L} w_l^{(m)} \ln q_l(\boldsymbol{y}_l^{(m)} \mid \boldsymbol{s}_l(z_l)) \Big], \tag{37}$$

where $\pi(\boldsymbol{z} \mid \boldsymbol{y}^{(m)}; \widehat{\boldsymbol{\theta}}_{i-1})$ is the posterior probability distribution of the methylation index vector $\boldsymbol{Z}$ given the nanopore data $\boldsymbol{y}^{(m)}$.

Maximization step: Given $J(\boldsymbol{\theta}; \widehat{\boldsymbol{\theta}}_{i-1})$, a new parameter estimate $\widehat{\boldsymbol{\theta}}_i$ is found by

$$\widehat{\boldsymbol{\theta}}_i = \arg\max_{\boldsymbol{\theta}} J(\boldsymbol{\theta}; \widehat{\boldsymbol{\theta}}_{i-1}). \tag{38}$$

CpelNano implements the expectation step as follows. From Eqs. (23) & (24) note that

$$\ln \pi(\boldsymbol{z}; \boldsymbol{\theta}) = -\ln \zeta(\boldsymbol{\theta}) + \alpha \phi_1(\boldsymbol{z}) + \beta \phi_2(\boldsymbol{z}) + \gamma \phi_3(\boldsymbol{z}), \tag{39}$$

where $\boldsymbol{\theta} = [\alpha\ \beta\ \gamma]^T$,

$$\phi_1(\boldsymbol{z}) := \sum_{l=1}^{L} N_l (2z_l - 1), \tag{40}$$

$$\phi_2(\boldsymbol{z}) := \sum_{l=1}^{L} \bar{\rho}_l N_l (2z_l - 1), \tag{41}$$

and

$$\phi_3(\boldsymbol{z}) := \sum_{l=1}^{L-1} (2z_l - 1)(2z_{l+1} - 1)/\bar{d}_l. \tag{42}$$

Consequently, Eq. (37) becomes

$$J(\boldsymbol{\theta}; \widehat{\boldsymbol{\theta}}_{i-1}) = -M \ln \zeta(\boldsymbol{\theta}) + \alpha \omega_1(\widehat{\boldsymbol{\theta}}_{i-1}) + \beta \omega_2(\widehat{\boldsymbol{\theta}}_{i-1}) + \gamma \omega_3(\widehat{\boldsymbol{\theta}}_{i-1}) + \omega_4(\widehat{\boldsymbol{\theta}}_{i-1}), \tag{43}$$

where

$$\omega_j(\widehat{\boldsymbol{\theta}}_{i-1}) := \sum_{m=1}^{M} \sum_{\boldsymbol{z} \in \mathcal{Z}} \phi_j(\boldsymbol{z}) \pi(\boldsymbol{z} \mid \boldsymbol{y}^{(m)}; \widehat{\boldsymbol{\theta}}_{i-1}), \quad \text{for } j = 1, 2, 3, \tag{44}$$

and

$$\omega_4(\widehat{\boldsymbol{\theta}}_{i-1}) := \sum_{m=1}^{M} \sum_{\boldsymbol{z} \in \mathcal{Z}} \phi_4^{(m)}(\boldsymbol{z}) \pi(\boldsymbol{z} \mid \boldsymbol{y}^{(m)}; \widehat{\boldsymbol{\theta}}_{i-1}), \tag{45}$$

with

$$\phi_4^{(m)}(\boldsymbol{z}) := \sum_{l=1}^{L} w_l^{(m)} \ln q_l(\boldsymbol{y}_l^{(m)} \mid \boldsymbol{s}_l(z_l)). \tag{46}$$

On the other hand, to implement the maximization step in Eq. (38), CpelNano sets the gradient of $J(\boldsymbol{\theta}; \widehat{\boldsymbol{\theta}}_{i-1})$ with respect to $\boldsymbol{\theta} = [\alpha\ \beta\ \gamma]^T$ equal to zero at $\boldsymbol{\theta} = \widehat{\boldsymbol{\theta}}_i$. In this case, and from Eqs. (24) & (25), as well as Eqs. (40)-(43), the following system of nonlinear equations are obtained

$$\sum_{\boldsymbol{z} \in \mathcal{Z}} \phi_j(\boldsymbol{z}) \pi(\boldsymbol{z}; \widehat{\boldsymbol{\theta}}_i) = \frac{1}{M} \omega_j(\widehat{\boldsymbol{\theta}}_{i-1}), \quad \text{for } j = 1, 2, 3, \tag{47}$$

which are solved for $\widehat{\boldsymbol{\theta}}_i$ by using NLsolve, v5.5.0 (`https://github.com/JuliaNLSolvers/NLsolve.jl`), a Julia implementation of the trust region approach[15]. Note that these formulas require evaluation of the posterior probability distribution $\pi(\boldsymbol{z} \mid \boldsymbol{y}; \boldsymbol{\theta})$ of the methylation index vector $\boldsymbol{Z}$, as well as evaluation of expectations and correlations of $\boldsymbol{Z}$ with respect to its prior and posterior probability distributions. We discuss these computations next.

4. Computational implementation

We now provide formulas associated with the general Ising model $p(\boldsymbol{x})$ given by Eqs. (1)-(3), which are used by CpelNano for efficient computations. These also apply to the CPEL model given by Eqs. (7)-(9), as well as to the reduced CPEL model given by Eqs. (23)-(25), since these models are special cases of the general Ising model. For example, the reduced CPEL model can be obtained from the general Ising model by replacing $\boldsymbol{x}$, n, and N in Eqs. (1)-(3) with $\boldsymbol{z}$, l, and L, respectively, and by setting $a_l = N_l(\alpha + \beta\bar{\rho}_l)$ and $b_l = \gamma/\bar{d}_l$.

Partition function. By employing the transfer matrix method[3], it can be shown that

$$\zeta = \boldsymbol{u}_1^T \boldsymbol{W}_1 \boldsymbol{W}_2 \cdots \boldsymbol{W}_{N-1} \boldsymbol{u}_N, \tag{48}$$

where

$$\boldsymbol{u}_1 = \begin{bmatrix} e^{-a_1/2} \\ e^{+a_1/2} \end{bmatrix}, \quad \boldsymbol{u}_N = \begin{bmatrix} e^{-a_N/2} \\ e^{+a_N/2} \end{bmatrix}, \tag{49}$$

and

$$\boldsymbol{W}_n = \begin{bmatrix} e^{-(a_n+a_{n+1})/2+b_n} & e^{-(a_n-a_{n+1})/2-b_n} \\ e^{+(a_n-a_{n+1})/2-b_n} & e^{+(a_n+a_{n+1})/2+b_n} \end{bmatrix}, \quad \text{for } n = 1, 2, \ldots, N-1. \tag{50}$$

This formula is used to compute partition functions by successive vector/matrix multiplications.

Expectations. CpelNano computes the expectations

$$e_n := \mathrm{E}[2X_n - 1], \ \text{ for } \ n = 1, 2, \ldots, N, \tag{51}$$

via successive vector/matrix multiplications using the following formula:

$$e_n = \frac{1}{\zeta}\, \boldsymbol{u}_1^T \boldsymbol{W}_1 \cdots \boldsymbol{W}_{n-1} \boldsymbol{W}_n^{(e)} \boldsymbol{W}_{n+1} \cdots \boldsymbol{W}_{N-1} \boldsymbol{u}_N, \tag{52}$$

where

$$\boldsymbol{W}_n^{(e)} = \boldsymbol{W}_n \circ \boldsymbol{E} \tag{53}$$

is the Hadamard product between matrices $\boldsymbol{W}_n$ and

$$\boldsymbol{E} = \begin{bmatrix} -1 & -1 \\ 1 & 1 \end{bmatrix}. \tag{54}$$

From the e_n's, the expectations $\mu_n := \mathrm{E}[X_n]$ are then computed by $\mu_n = (e_n + 1)/2$.

Correlations. The pairwise correlations

$$c_n := \mathrm{E}[(2X_n - 1)(2X_{n+1} - 1)], \text{ for } n = 1, 2, \ldots, N-1, \tag{55}$$

are computed via successive vector/matrix multiplications using the following formula:

$$c_n = \frac{1}{\zeta} \boldsymbol{u}_1^T \boldsymbol{W}_1 \cdots \boldsymbol{W}_{n-1} \boldsymbol{W}_n^{(c)} \boldsymbol{W}_{n+1} \cdots \boldsymbol{W}_{N-1} \boldsymbol{u}_N, \tag{56}$$

where

$$\boldsymbol{W}_n^{(c)} = \boldsymbol{W}_n \circ \boldsymbol{C} \tag{57}$$

is the Hadamard product between matrices $\boldsymbol{W}_n$ and

$$\boldsymbol{C} = \begin{bmatrix} 1 & -1 \\ -1 & 1 \end{bmatrix}. \tag{58}$$

From the c_n's, the pairwise correlations $r_n := \mathrm{E}[X_n X_{n+1}]$ are then computed by $r_n = [c_n + 2(\mu_n + \mu_{n+1}) - 1]/4$.

Sampling. The general Ising model is equivalent to a first-order Markov chain with *inhomogeneous* transition probabilities. Indeed, it can be shown that

$$\Pr[X_n = x_n \mid X_{n-1} = x_{n-1}, \ldots, X_1 = x_1] = \begin{cases} p_1(x_1), & \text{for } n = 1 \\ p_n(x_n \mid x_{n-1}), & \text{for } n = 2, 3, \ldots, N, \end{cases} \tag{59}$$

where

$$p_1(x_1) = \frac{w_1(x_1) \exp\{a_1 x_1\}}{\sum_u w_1(u) \exp\{a_1 u\}}, \tag{60}$$

and

$$p_n(x_n \mid x_{n-1}) = \frac{w_n(x_n) \exp\{a_n(2x_n - 1) + b_{n-1}(2x_{n-1} - 1)(2x_n - 1)\}}{\sum_u w_n(u) \exp\{a_n(2u - 1) + b_{n-1}(2x_{n-1} - 1)(2u - 1)\}}, \tag{61}$$

for $n = 2, 3, \ldots, N$, with

$$w_n(x_n) = \sum_{x_{n+1}} \cdots \sum_{x_N} \exp\left\{ \sum_{n'=n+1}^{N} a_{n'}(2x_{n'} - 1) + \sum_{n'=n}^{N-1} b_{n'}(2x_{n'} - 1)(2x_{n'+1} - 1) \right\}, \tag{62}$$

for $n = 1, 2, \ldots, N-1$, and

$$w_N(x_N) = 1. \tag{63}$$

Notably, function $w_n(x_n)$ is efficiently computed by CpelNano using the following matrix/vector formula:

$$w_n(x_n) = \boldsymbol{g}_n^T(2x_n - 1)\, \boldsymbol{G}_n(2x_n - 1) \boldsymbol{W}_{n+2} \boldsymbol{W}_{n+3} \cdots \boldsymbol{W}_{N-1} \boldsymbol{u}_N, \tag{64}$$

where

$$\boldsymbol{g}_n(x) = \begin{bmatrix} e^{-(a_{n+1}+b_n x)/2} \\ e^{+(a_{n+1}+b_n x)/2} \end{bmatrix}, \tag{65}$$

and

$$\boldsymbol{G}_n(x) = \begin{bmatrix} e^{-(a_{n+1}+b_n x)/2 - a_{n+2}/2 + b_{n+1}} & e^{-(a_{n+1}+b_n x)/2 + a_{n+2}/2 - b_{n+1}} \\ e^{+(a_{n+1}+b_n x)/2 - a_{n+2}/2 - b_{n+1}} & e^{+(a_{n+1}+b_n x)/2 + a_{n+2}/2 + b_{n+1}} \end{bmatrix}. \tag{66}$$

Consequently,

$$p(\boldsymbol{x}) = p_1(x_1) \prod_{n=2}^{N} p_n(x_n \mid x_{n-1}), \tag{67}$$

which allows CpelNano to recursively draw a sample $\boldsymbol{x}$ of the methylation state $\boldsymbol{X}$ from the Ising model $p(\boldsymbol{x})$, by first drawing a sample x_1 from the initial probability distribution $p_1(x_1)$ and by sequentially drawing samples x_n, $n = 2, 3, \ldots, N$, from the transition probabilities $p_n(x_n \mid x_{n-1})$.

Marginalization. From the general Ising model $p(\boldsymbol{x})$ of the methylation state $\boldsymbol{X} = [X_1, X_2, \ldots, X_N]^T$ over an estimation region $\mathcal{R}$, the probability distribution $g(\boldsymbol{x}')$ of the methylation state $\boldsymbol{X}' = [X_k, X_{k+1}, \ldots, X_{k+K-1}]^T$ over a subregion $\mathcal{S}$ of $\mathcal{R}$ that contains K contiguous CpG sites $k, k+1, \ldots, k+K-1$ can be obtained using marginalization; i.e., by setting $g(\boldsymbol{x}') = \sum_{\boldsymbol{x}''} p(\boldsymbol{x})$, where $\boldsymbol{x}'' = [x_1, \ldots, x_{k-1}, x_{k+K}, \ldots, x_N]^T$. From Eqs. (1)-(3), it can be shown that

$$g(\boldsymbol{x}') = \frac{\zeta_1(x_k)\zeta_2(x_{k+K-1})}{\zeta} \exp\{-W(\boldsymbol{x}')\}, \tag{68}$$

where ζ is the partition function of the general Ising model, given by Eqs. (2) & (3), and

$$W(\boldsymbol{x}') = -\sum_{n=k}^{k+K-1} a_n(2x_n - 1) - \sum_{n=k}^{k+K-2} b_n(2x_n - 1)(2x_{n+1} - 1), \tag{69}$$

$$\zeta_1(x_k) = \sum_{\boldsymbol{x}_1''} \exp\Big\{ \sum_{n=1}^{k-1} a_n(2x_n - 1) + \sum_{n=1}^{k-1} b_n(2x_n - 1)(2x_{n+1} - 1) \Big\}, \tag{70}$$

$$\zeta_2(x_{k+K-1}) = \sum_{\boldsymbol{x}''} \exp\Big\{ \sum_{n=k+K}^{N} a_n(2x_n - 1) + \sum_{n=k+K-1}^{N-1} b_n(2x_n - 1)(2x_{n+1} - 1) \Big\}, \tag{71}$$

with $\boldsymbol{x}_1'' = [x_1, x_2, \ldots, x_{k-1}]^T$ and $\boldsymbol{x}_2'' = [x_{k+K}, x_{k+K+1}, \ldots, x_N]^T$. Efficient computation of the the partition functions $\zeta_1(x)$ and $\zeta_2(x)$ for $x = 0, 1$ is performed by employing formulas similar to the one used to compute the partition function ζ.

Posterior distribution of methylation indices. To compute the posterior distribution $\pi(\boldsymbol{z} \,|\, \boldsymbol{y}; \boldsymbol{\theta})$ of the methylation index vector $\boldsymbol{Z}$, note that

$$\pi(\boldsymbol{z} \mid \boldsymbol{y}; \boldsymbol{\theta}) = \frac{q(\boldsymbol{y} \mid \boldsymbol{z})\pi(\boldsymbol{z}; \boldsymbol{\theta})}{\sum_{\boldsymbol{z}' \in \mathcal{Z}} q(\boldsymbol{y} \mid \boldsymbol{z}')\pi(\boldsymbol{z}'; \boldsymbol{\theta})}. \tag{72}$$

This equation, together with Eqs. (23), (24), & (36), implies that

$$\pi(\boldsymbol{z} \mid \boldsymbol{y}; \boldsymbol{\theta}) = \frac{1}{\zeta(\boldsymbol{\theta}, \boldsymbol{y})} \exp\{-V(\boldsymbol{z}; \boldsymbol{\theta}, \boldsymbol{y})\}, \tag{73}$$

where

$$V(\boldsymbol{z}; \boldsymbol{\theta}, \boldsymbol{y}) = -\sum_{l=1}^{L} A_l(z_l; \alpha, \beta, \boldsymbol{y})(2z_l - 1) - \sum_{l=1}^{L-1} B_l(2z_l - 1)(2z_{l+1} - 1), \tag{74}$$

and

$$\zeta(\boldsymbol{\theta}, \boldsymbol{y}) = \sum_{\boldsymbol{z} \in \mathcal{Z}} \exp\{-V(\boldsymbol{z}; \boldsymbol{\theta}, \boldsymbol{y})\}, \tag{75}$$

with

$$A_l(z_l; \alpha, \beta, \boldsymbol{y}) = N_l(\alpha + \beta \bar{\rho}_l) + w_l^{(m)} \frac{\ln q_l(\boldsymbol{y}_l \mid \boldsymbol{s}_l(z_l))}{2z_l - 1}, \tag{76}$$

and

$$B_l = \gamma / \bar{d}_l. \tag{77}$$

Consequently, $\pi(\boldsymbol{z} \mid \boldsymbol{y}; \boldsymbol{\theta})$ is the probability distribution of an Ising model obtained by replacing $\boldsymbol{x}$, n, and N in Eqs. (1)-(3) with $\boldsymbol{z}$, l, and L, respectively, and by setting $a_l = A_l(z_l; \alpha, \beta, \boldsymbol{y})$ and $b_l = B_l$. In this case, computations can be efficiently preformed using the previous formulas by replacing N with L, $-a_n$ with $A_l(0; \alpha, \beta, \boldsymbol{y})$, $+a_n$ with $A_l(1; \alpha, \beta, \boldsymbol{y})$, and b_n with B_l.

Mean methylation level. CpelNano computes the mean methylation level (MML) μ over a subregion $\mathcal{S}$ of an estimation region $\mathcal{R}$ that contains K contiguous CpG sites $k, k+1, \ldots, k+K-1$ by setting

$$\mu = \frac{1}{K} \sum_{n=k}^{k+K-1} \mu_n. \tag{78}$$

Here, μ_n is the mean methylation at CpG site n, which is calculated by setting $\mu_n = (e_n + 1)/2$, where e_n is computed from Eqs. (52)-(54).

Normalized methylation entropy. To compute the normalized methylation entropy (NME) h over a subregion $\mathcal{S}$ of an estimation region $\mathcal{R}$ that contains K contiguous CpG sites $k, k+1, \ldots, k+K-1$, CpelNano uses the following formula:

$$\begin{aligned} h = \frac{1}{K\ln 2}\Big\{ & \ln\zeta - (1-\mu_k)\ln\zeta_1(0) - \mu_k \ln\zeta_1(1) \\ & - (1-\mu_{k+K-1})\ln\zeta_2(0) - \mu_{k+K-1}\ln\zeta_2(1) \\ & - \sum_{n=k}^{k+K-1}(\alpha+\beta\rho_n)(2\mu_n - 1) - \sum_{n=k}^{k+K-2}\frac{\gamma}{d_n}[4r_n - 2(\mu_n+\mu_{n+1})+1]\Big\}. \end{aligned} \tag{79}$$

Here, μ_n is the mean methylation at CpG site n, r_n is the pairwise correlation between CpG sites n and $n+1$, which is calculated by setting $r_n = [c_n + 2(\mu_n + \mu_{n+1}) - 1]/4$, where c_n is computed from Eqs. (56)-(58), and ζ, ζ_1, ζ_2 are the partition functions computed from Eqs. (48)-(50), as well as Eqs. (70) & (71).

Coefficient of methylation divergence. CpelNano also computes the coefficient of methylation divergence (CMD), d_{12}, between two probability distributions g_1 and g_2 of the methylation state over a subregion $\mathcal{S}$ of an estimation region $\mathcal{R}$ that contains K contiguous CpG sites $k, k+1, \ldots, k+K-1$, which are obtained by marginalizing two CPEL models, p_1 and p_2, of the methylation state over $\mathcal{R}$. This quantity is defined by

$$d_{12} := \frac{D(g_1 \parallel \overline{g}) + D(g_2 \parallel \overline{g})}{H(g_1, \overline{g}) + H(g_2, \overline{g})}, \tag{80}$$

where $\overline{g}(\boldsymbol{x})$ is a probability distribution of the methylation state over $\mathcal{S}$, which is obtained by marginalizing a CPEL model $\overline{p}$ whose potential energy function is the *average* of the potential energy functions associated with p_1 and p_2. Moreover,

$$D(f_1 \parallel f_2) = \sum_{\boldsymbol{u}} f_1(\boldsymbol{u}) \log_2 \frac{f_1(\boldsymbol{u})}{f_2(\boldsymbol{u})} \tag{81}$$

is the Kullback-Leibler (KL) divergence between two probability distributions f_1 and f_2, and

$$H(f_1, f_2) = -\sum_{\boldsymbol{u}} f_1(\boldsymbol{u}) \log_2 f_2(\boldsymbol{u}) \tag{82}$$

is the cross-entropy between two random vectors with probability distributionsf_1 and f_2.

It can be shown that

$$d_{12} = 1 - \frac{h_1 + h_2}{\overline{h}_1 + \overline{h}_2}, \tag{83}$$

where h_i is the NME associated with the the i-th probability distribution g_i, and $\overline{h}_i$ is the normalized cross methylation entropy between g_i and $\overline{g}$, which is the formula used by CpelNano to compute the CMD. This is done by evaluating, in addition to the NMEs h_1 and h_2, the normalized cross methylation entropies $\overline{h}_1$ and $\overline{h}_2$ by means of

$$\begin{aligned}\overline{h}_i = \frac{1}{K\ln 2}\Big\{ & \ln\overline{\zeta} - (1-\mu_k^{(i)})\ln\overline{\zeta}_1(0) - \mu_k^{(i)}\ln\overline{\zeta}_1(1) \\ & -(1-\mu_{k+K-1}^{(i)})\ln\overline{\zeta}_2(0) - \mu_{k+K-1}^{(i)}\ln\overline{\zeta}_2(1) - \sum_{n=k}^{k+K-1}(\overline{\alpha}+\overline{\beta}\rho_n)(2\mu_n^{(i)}-1) \\ & -\sum_{n=k}^{k+K-2}\frac{\overline{\gamma}}{d_n}[4r_n^{(i)} - 2(\mu_n^{(i)}+\mu_{n+1}^{(i)})+1]\Big\}, \quad \text{for } i = 1, 2,\end{aligned} \tag{84}$$

which is similar to Eq. (79). Here, $\overline{\alpha}$ is the average of the two α parameters associated with the potential energy functions of the CPEL models p_1 and p_2, and similarly for $\overline{\beta}$ and $\overline{\gamma}$. Moreover, $\overline{\zeta}$ is the partition function of $\overline{p}$, $\overline{\zeta}_1$ and $\overline{\zeta}_2$ are the partition functions obtained by marginalizing $\overline{p}$ within the subregion $\mathcal{S}$ using Eqs. (70) & (71), and $\mu_n^{(i)}$, $r_n^{(i)}$ are the mean methylation and pairwise correlation at CpG site n associated with p_i.

5. Hypothesis testing

CpelNano is designed to identify analysis regions that demonstrate significant discordance in DNA methylation between two conditions (e.g., normal/cancer) by means of a hypothesis testing approach that uses permutation methods[16] to perform unmatched sample pairs group comparisons, matched sample pairs group comparisons, or two-sample comparisons, depending on the particular experimental design used in a given application (details follow). Due to multiple hypothesis testing, and in addition to P-values, CpelNano also computes Q-values using the Benjamini-Hochberg procedure for FDR control.

Unmatched sample pairs group comparison. When a group of M_1 nanopore samples associated with one condition (e.g., normal) and a group of M_2 *unmatched* nanopore samples associated with another condition (e.g., cancer) are available, CpelNano performs hypothesis

testing in each analysis region using the following differential test statistics:

$$T_{\text{MML}} = \frac{1}{M_1} \sum_{m=1}^{M_1} \mu_1^{(m)} - \frac{1}{M_2} \sum_{m=1}^{M_2} \mu_2^{(m)} \tag{85}$$

$$T_{\text{NME}} = \frac{1}{M_1} \sum_{m=1}^{M_1} h_1^{(m)} - \frac{1}{M_2} \sum_{m=1}^{M_2} h_2^{(m)} \tag{86}$$

$$T_{\text{CMD}} = \frac{1}{M_1 M_2} \sum_{m=1}^{M_1} \sum_{m'=1}^{M_2} d_{mm'}. \tag{87}$$

In these formulas, $\mu_1^{(m)}$, $h_1^{(m)}$ and $\mu_2^{(m)}$, $h_2^{(m)}$ are the MMLs and NMEs computed using the CPEL models estimated from the m-th sample in the first group and the m-th sample in the second group, and $d_{mm'}$ is the CMD obtained by comparing the estimated probability distributions of methylation associated with the m-th sample in the first group and the m'-th sample in the second group. The test statistic T_{MML} quantifies the difference between the average of the mean methylation levels in the first and second groups, T_{NME} assesses the difference between the average of normalized methylation entropies, and T_{CMD} quantifies the average of all observed differences between the probability distributions of methylation in the two groups. Notably, this approach requires a total of $M_1 + M_2$ CPEL model estimations.

For each test statistic T, a (two-tailed) hypothesis test requires knowledge of the null cumulative distribution function $F_0(t) = \Pr[\, |T| < t \mid H_0 \,]$, which can then be used to calculate the P-value associated with an observation t^* of T by $p = 1 - F_0(|t^*|)$. Here, H_0 is the null hypothesis that, within an analysis region, each pair of samples exhibits no methylation discordance regardless of the specific group sample assignment. To evaluate $F_0(t)$ for an analysis region, CpelNano uses a "randomization model" that randomly assigns M_1 samples to the first condition and the remaining M_2 samples to the second condition, thus forming $L = (M_1 + M_2)!/M_1!M_2!$ group assignments. This permutation is justified by the fact that, under the null hypothesis, the assignments are equally likely (with probability $1/L$). Consequently, CpelNano computes the null cumulative distribution function of the test statistic T by

$$F_0(t) = \frac{1}{L} \sum_{l=1}^{L} I[\, |t_l| < t \,], \tag{88}$$

where $t_l, l = 1, 2, \ldots, L$, are values of T computed from each of the L group assignments and $I[\cdot]$ is the Iverson bracket, taking value 1 when its argument is true and 0 otherwise. This leads

to an *exact* P-value computation, given by

$$p = 1 - \frac{1}{L}\sum_{l=1}^{L} I[\,|t_l| < |t^*|\,] = \frac{1}{L}\Big(1 + \sum_{l=1}^{L-1} I[\,|t_l| \geq |t^*|\,]\Big). \quad (89)$$

Notably, p can only take values $1/L, 2/L, \ldots, 1$ and, therefore, this method produces P-values that are not smaller than $1/L$. Moreover, by setting the test's significance level to be $a = k/L$, for some integer k such that p can take value k/L, it can be shown that the probability of the Type I error (false positives) will be given by

$$\Pr[\text{Type I error}] = \Pr\big[P \leq a \mid H_0\big] = \sum_{l=1}^{k} \Pr\big[P = k/L \mid H_0\big] = \sum_{l=1}^{k} 1/L = \frac{k}{L} = a, \quad (90)$$

leading to a false positive rate of $100 \times a$ %. On the other hand, if $a = k/L$, for some integer k such that p cannot take value k/L, then $\Pr[\text{Type I error}] < a$ and the test will be conservative. Therefore, the hypothesis testing module of CpelNano can always control the false positive rate in this case by using an appropriate value for the test's significance level. For our real data analysis in the Main Text, we had $M_1 = M_2 = 5$ and set $a = 0.05$, which implies that the P-values will be no smaller than 3.96×10^{-3} and the false positive rate will be 4.76%, since $L = 252$ and $k = 12$ in this case.

When L is large, the previous method becomes computationally intensive. For this reason, and when $L \geq L_0$ (L_0 is set to 1,000 by default), CpelNano automatically switches to a hypothesis testing approach that estimates the P-value using a permutation test based on Monte Carlo sampling. In this case, L distinct sample permutations are performed by assigning M_1 samples to the first group and the remaining samples to the second group. The null cumulative distribution function is then approximated by independently sampling, $L_0 - 1$ times, the set of L permutations with equal probability and by approximating $F_0(t)$ by

$$\widehat{F}_0(t) = \frac{1}{L_0}\Big(I[\,|t^*| < t\,] + \sum_{l=1}^{L_0-1} I[\,|t_l| < t\,]\Big), \quad (91)$$

since this method produces L_0 test statistic values, including the value t^* computed from the data. In this case, the P-value is approximated by

$$\widehat{p} = 1 - \frac{1}{L_0}\sum_{l=1}^{L_0-1} I[\,|t_l| < |t^*|\,] = \frac{1}{L_0}\Big(1 + \sum_{l=1}^{L_0-1} I[\,|t_l| \geq |t^*|\,]\Big). \quad (92)$$

Note that the only possible values for $\widehat{p}$ are $1/L_0, 2/L_0, \ldots, 1$, which implies that $\widehat{p} \geq 0.001$ when $L_0 = 1{,}000$. Moreover, if the significance level of the test is taken to be $a = k/L_0$ for

some integer k such that $\widehat{p}$ can take value k/L_0, then it can be shown that $\Pr[\text{Type I error}] = a$, whereas if the significance level is taken to be $a = k/L_0$ for some integer k such that $\widehat{p}$ cannot take a value k/L_0, then $\Pr[\text{Type I error}] < a$. Consequently, this procedure also controls the Type I error.

Matched sample pairs group comparison. CpelNano can also perform hypothesis testing within an analysis region when M pairs of *matched* nanopore samples between two conditions are available. This is done by using the previous randomization testing method, provided that the total number $L = 2^M$ of possible matched group assignments is less than L_0. In this case, the M matched sample pairs are used to form L distinct permutations, each containing all M pairs but with some group labels being reversed, values t_l are computed for the l-th permutation using each of the following two differential test statistics:

$$T_{\text{MML}} = \frac{1}{M} \sum_{m=1}^{M} \big[\mu_1^{(m)} - \mu_2^{(m)}\big] \quad (93)$$

$$T_{\text{NME}} = \frac{1}{M} \sum_{m=1}^{M} \big[h_1^{(m)} - h_2^{(m)}\big]. \quad (94)$$

However, if $L \geq L_0$, CpelNano automatically switches to the Monte Carlo based permutation procedure employed in the unmatched case in which the $L_0 - 1$ values of the test statistic required by the method are determined by independently sampling the set of all L matched group permutations with equal probability and by computing the test statistic value for each permutation.

Unfortunately, this procedure cannot be used to perform hypothesis testing using the CMD because of its symmetry; i.e., due to the fact that $d_{mm'} = d_{m'm}$. For this reason, CpelNano simply calculates the average $\big(\sum_{m=1}^{M} d_{mm}\big)/M$ of all CMDs associated with the analysis regions and outputs the result.

Two-sample comparison. CpelNano can perform hypothesis testing within an analysis region even when only one sample is available for each condition. It does so by employing the same "randomization model" used in the case of the unmatched sample pairs group comparison for which M_1 is the number of nanopore reads associated with the first condition overlapping the analysis region and M_2 is the number of reads associated with the second condition. In this case, evaluation of the null cumulative distribution function $F_0(t)$ is performed by randomly assigning M_1 nanopore reads to the first condition and the remaining M_2 samples to the second condition. However, evaluating the MMLs, NMEs, and probability distributions of

methylation necessary for computing the three test statistics given by Eqs. (85)-(87) requires $(M_1 + M_2)!/M_1!M_2!$ CPEL model estimations in this case, which can be much larger than the $L = M_1 + M_2$ CPEL model estimations required by the unmatched sample pairs group comparison. For this reason, CpelNano automatically switches to the Monte Carlo version of the permutation test when $L \geq L_0$, where L_0 is now set to 100 by default.

6. Simulation-based evaluation of Nanopolish

To evaluate the methylation calling performance of Nanopolish[1], we developed a simulation-based benchmarking approach (Fig. 3), which can be appropriately modified to accommodate other methylation callers if desired. This scheme employs the GSM2308632 WGBS data identified with the well-characterized human GM12878 Utah/Ceph lymphoblastoid cell line and constructs a "ground-truth" CPEL methylation model, given by Eqs. (7)-(9), within Chr. 22 of the human reference genome that contains 622,083 CpG sites. It does so by estimating the parameters α, β, and γ of the CPEL model, using a previous maximum-likelihood approach[6,7], over 3-kb estimation regions that contain sufficient data to perform reliable estimation (specifically, regions that contain at least 10 CpG sites, with an average coverage of at least $5\times$ per CpG site, and for which methylation information is available for at least $2/3$ of their CpG sites). To determine parameter values within the remaining estimation regions, the method uses all estimated α, β, and γ values and computes their empirical probability distributions (Fig. 4). It then assigns parameter values to these regions by drawing samples from the corresponding empirical distributions.

To generate DNA fragments that satisfy the length distribution and coverage requirements of nanopore reads, the benchmarking method computes the length distribution of nanopore reads in available nanopore sequencing data (NA12878) identified with the GM12878 Utah/Ceph lymphoblastoid cell line[17]. In agreement with Li et al.[18,19], the read lengths in these data were found to follow an exponential distribution with rate 1.18×10^{-4}, which was estimated from the real data via maximum-likelihood (Fig. 5). The method then produces DNA fragments by determining their start location and length along Chr. 22. The start location of a fragment is specified by randomly drawing a number between 1 and $L - M + 1$, where $L = 50{,}818{,}468$ bp is the length of Chr. 22, and its length M (in bp) is computed by sampling the previous exponential length distribution. To control for methylation coverage, fragment generation is repeated until the average of all nucleotide coverages within Chr. 22 is no less than $25\times$, with the coverage at each nucleotide being computed as the number of DNA fragments overlapping the nucleotide.

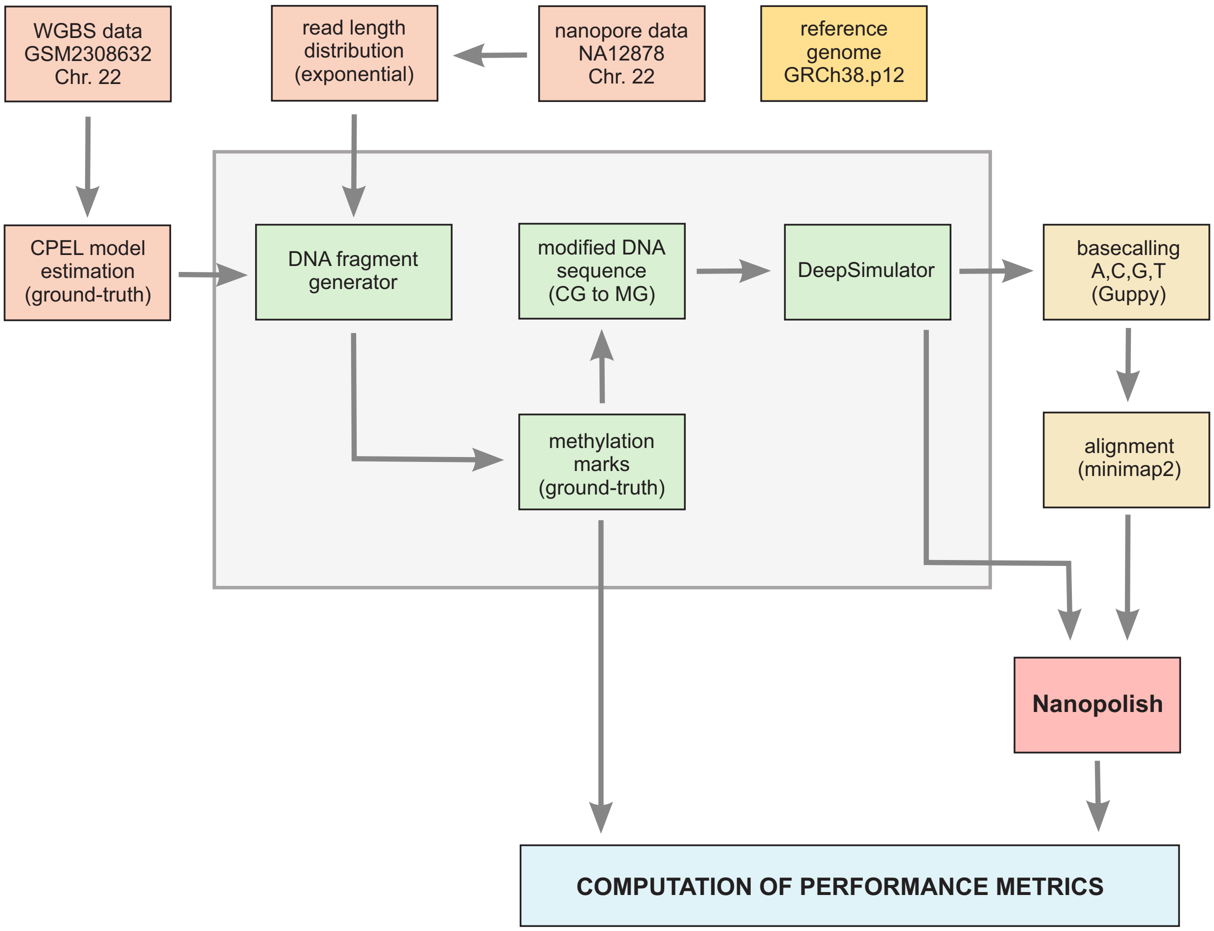

Figure 3. Scheme for benchmarking Nanopolish[1]. Simulation-based benchmarking method for evaluating the methylation calling performance of Nanopolish[1]. This approach uses human cell-line WGBS and nanopore sequencing data to generate DNA fragments of known methylation states, which are then processed by the DeepSimulator[18,19] to produce realistic nanopore reads. Evaluation is performed by comparing the output of the caller to the known ground-truth methylation states of the input DNA fragments that generate these reads.

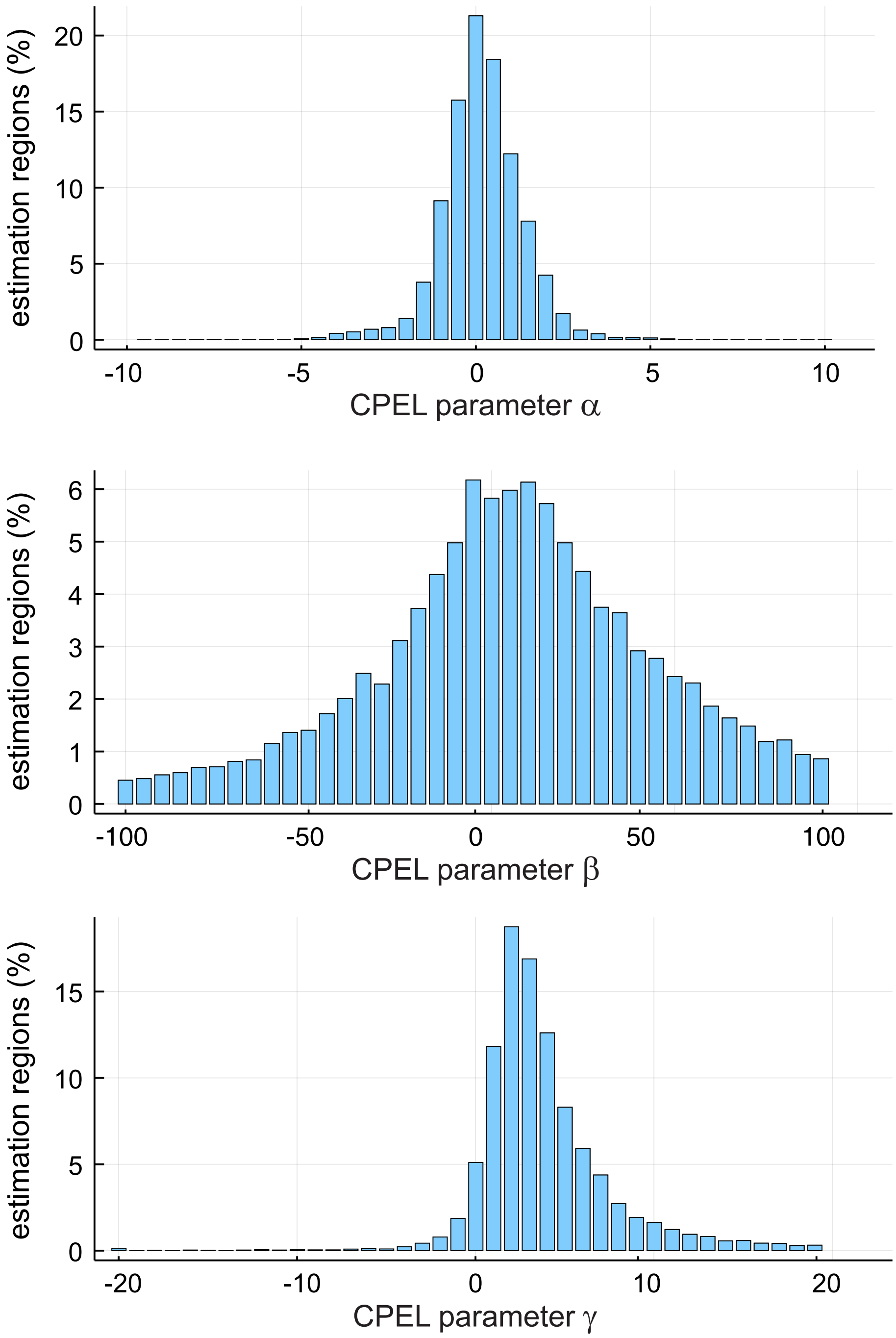

Figure 4. Distributions of CPEL model parameter values. Distributions of the values of the CPEL model parameters α, β, and γ in Chr. 22, which are estimated via maximum-likelihood from human Utah/Ceph lymphoblastoid WGBS data (GSM2308632).

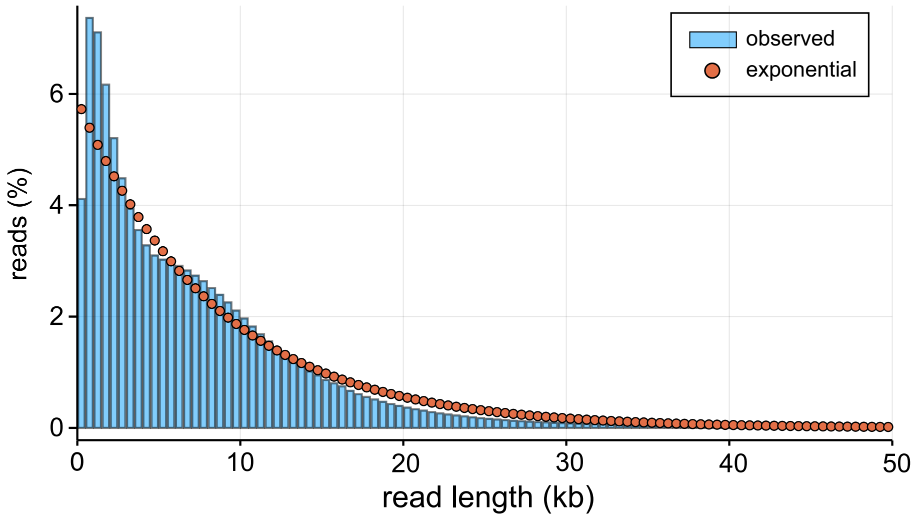

Figure 5. Length distribution of nanopore reads. Distribution of read lengths observed in the NA12878 human Utah/Ceph lymphoblastoid nanopore data (blue) was found to be approximately exponential (red dots) with rate 1.18×10^{-4}.

Subsequently, and for each DNA fragment, the benchmarking method identifies all estimation regions that overlap the fragment and, for each estimation region, it generates its methylation state by sampling the ground-truth CPEL model associated with that region using the Markov chain sampling approach discussed in Section 4. This information is then used to assign a ground-truth binary methylation state to each CpG site in the DNA fragment by marking its CG dinucleotides as being methylated (1) or unmethylated (0) based on their methylation status in the associated estimation regions. Finally, and within each DNA fragment, the C's of all CG dinucleotides marked by 1 are replaced with M's, a step that modifies the DNA sequence in each fragment by incorporating the methylation of CG dinucleotides, as determined by the methylation states drawn from the ground-truth CPEL model.

Each modified DNA fragment generated by the previous approach is processed by the DeepSimulator[18,19] (used in its context-independent mode), a computational tool that faithfully simulates the entire pipeline of nanopore sequencing and produces nanopore reads consisting of the current values measured by the nanopore. However, and in order to take into account methylated 6-mers, the pore model used by DeeepSignal, which is based on official statistics provided by Oxford Nanopore Technologies, is replaced with the one used by Nanopolish[1]. In addition, raw nanopore reads are generated by adding random noise on the event sequence using the default option of the DeepSimulator[18,19] and by setting the cutoff frequency of the low-pass filter, which removes high-frequency components from the signal generated from the

event sequence, to its default value. Finally, and in order to investigate the effect of nanopore noise on methylation calling, zero-mean Gaussian noise is added to the raw nanopore reads with standard deviations 2, 2.5, 3, and 3.5, which encompasses values that are normally observed in the pore model used by Nanopolish[1].

The raw nanopore reads produced by the DeepSimulator[18,19] are subsequently used to perform base calling via ONT's Guppy (CPU mode) whose output is then aligned to the reference genome (GRCh38.p12) by minmap2[20]. The aligned data, together with the raw nanopore reads produced by the DeepSimulator[18,19], are then fed as inputs to Nanopolish[1] whose output is used to quantify methylation calling performance by computing several performance metrics, which include accuracy (probability that a CpG site is correctly predicted to be methylated or unmethylated), precision (probability that a CpG site is correctly predicted to be methylated), true positive rate, and true negative rate.

References

1. Simpson, J. T. et al. Detecting DNA cytosine methylation using nanopore sequencing. *Nat. Methods* **14**, 407-410 (2017).
2. Pressé, S., Ghosh, K., Lee, J. & Dill, K. A. Principles of maximum entropy and maximum caliber in statistical physics. *Rev. Mod. Phys.* **85**, 1115–1141 (2013).
3. Baxter, R. J. Exactly Solved Models in Statistical Mechanics (Academic Press, San Diego, 1982).
4. Lökvist, C., Dodd, I. B., Sneppen, K. & Haerter, J. O. DNA methylation in human epigenomes depends on local topology of CpG sites. *Nucleic Acids Res.* **44**, 5123–5132 (2016).
5. Affinito, O. et al. Nucleotide distance influences co-methylation between nearby CpG sites. *Genomics* **112**, 144-150 (2020).
6. Jenkinson, G., Pujadas, E., Goutsias, J. & Feinberg, A. P. Potential energy landscapes identify the information-theoretic nature of the epigenome. *Nat. Genetics* **49**, 719–729 (2017).
7. Jenkinson, G., Abante, J., Feinberg, A. P. & Goutsias, J. An information-theoretic approach to the modeling and analysis of whole-genome bisulfite sequencing data. *BMC Bioinformatics* **19**, 87 (2018).

8. Bickel, P. J. and Doksum, K. A. Mathematical Statistics. Basic Ideas and Selected Topics. Vol. I, Second Edition (CRC Press, Boca Raton, 2015).
9. Zhang, Y. et al. DNA methylation analysis of chromosome 21 gene promoters at single base pair and single allele resolution. *PLoS Genet.* **5**, e1000438 (2009).
10. Bell, J. T. et al. DNA methylation patterns associate with genetic and gene expression variation in HapMap cell lines. *Genome Biol.* **12**, R10 (2011).
11. Loman, N. J., Quick, J. & Simpson, J. T. A complete bacterial genome assembled *de novo* using only nanopore sequencing data. *Nat. Methods* **12**, 733–735 (2015).
12. Liu, Q. et al. Detection of DNA base modifications by deep recurrent neural network on Oxford Nanopore sequencing data. *Nat. Commun.* **10**, 2449 (2019).
13. Ni, P. et al. DeepSignal: detecting DNA methylation state from Nanopore sequencing reads using deep-learning. *Bioinformatics* **35**, 4586-4595 (2019).
14. Gigante, S. et al. Using long-read sequencing to detect imprinted DNA methylation. *Nucleic Acids Res.* **47**, e46 (2019).
15. Nocedal, J. & Wright, S. J. Numerical Optimization, Second Edition (Springer, New York, 2006).
16. Ernst, M.D. Permutation methods: A basis for exact inference. *Stat. Sci.* **19**, 676-68 (2004).
17. Jain, M. *et al.* Nanopore sequencing and assembly of a human genome with ultra-long reads. *Nat. Biotechnol.* **36**, 338-345 (2018).
18. Li, Y. *et al.* DeepSimulator: a deep simulator for Nanopore sequencing. *Bioinformatics* **34**, 2899-2908 (2018).
19. Li, Y. *et al.* DeepSimulator1.5: a more powerful, quicker and lighter simulator for Nanopore sequencing. *Bioinformatics* **36**, 2578–2580 (2020).
20. Li, H. Minimap2: pairwise alignment for nucleotide sequences. *Bioinformatics* **34**, 3094-3100 (2018).

Supplementary Figures

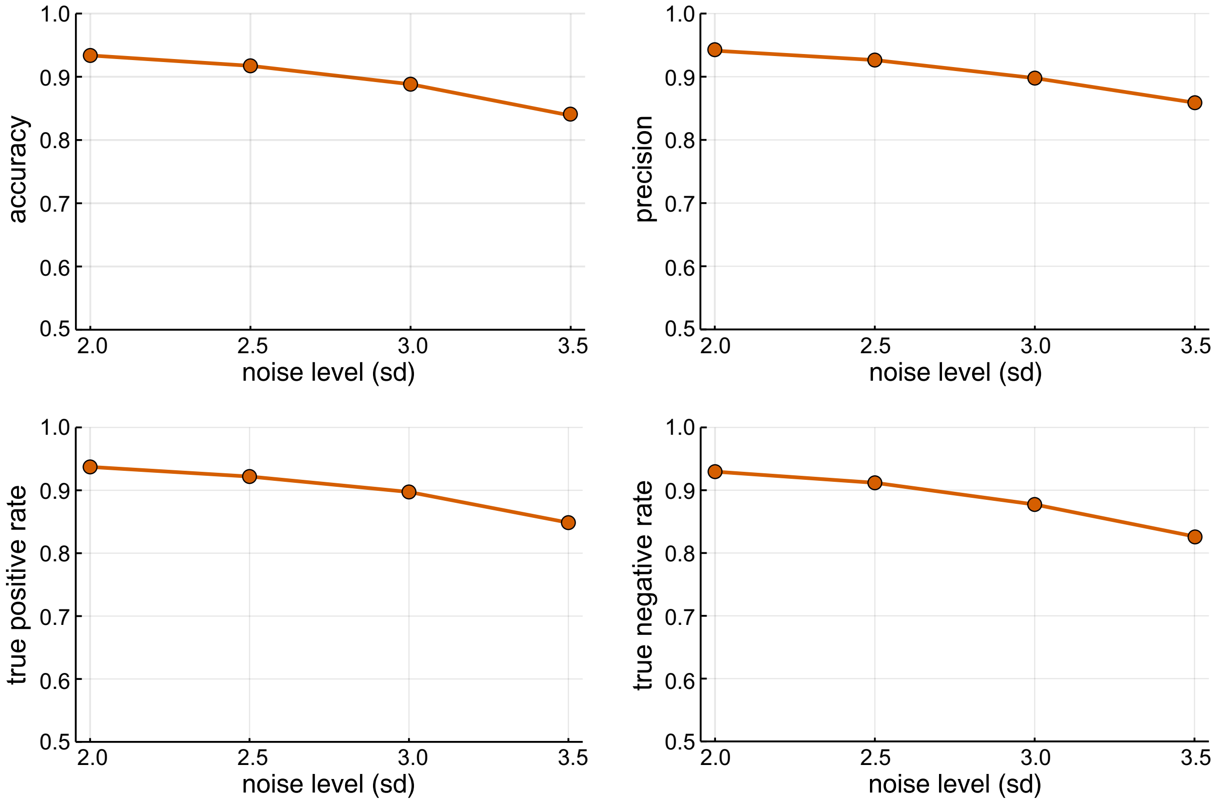

Figure S1. Performance characteristics of Nanopolish[1]. Accuracy (probability that a CpG site is correctly predicted to be methylated or unmethylated), precision (probability that a CpG site is correctly predicted to be methylated), true positive rate, and true negative rate characteristics for nanopore noise with standard deviations sd $= 2$, 2.5, 3, 3.5. The results were obtained by using our simulation-based scheme for benchmarking Nanopolish[1] (Supplementary Methods, Fig. 3) and by setting the detection threshold of Nanopolish[1] equal to zero. Nanopolish[1] exhibited reduced per-read detection performance at higher levels of nanopore noise and achieved no more that 94% accuracy, precision, true positive rate, and true negative rate at all noise levels, which dropped to less than 90% for sd ≥ 3.

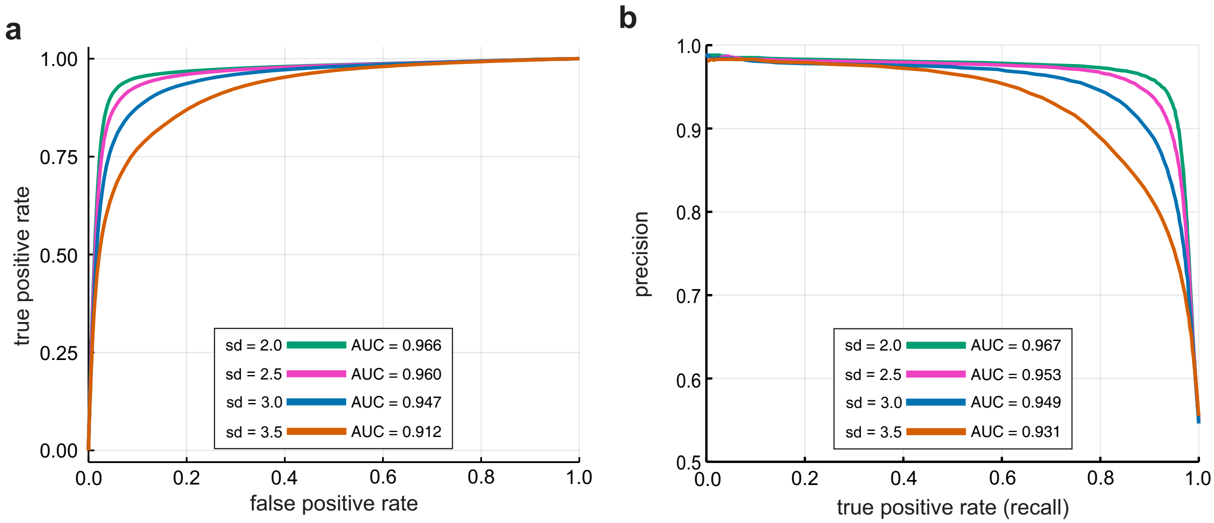

Figure S2. Detection performance of Nanopolish[1]. (**a**) Receiver operating characteristic (ROC) curves; (**b**) precision-recall (PR) curves. These results were obtained by using our simulation-based scheme for benchmarking Nanopolish[1] (Supplementary Methods, Fig. 3), by considering nanopore noise with standard deviations sd $= 2, 2.5, 3, 3.5$, and by varying the detection threshold of Nanopolish[1]. The area under the curve (AUC) values are also provided in each case. The results show a trade-off between the true positive rate and the false positive rate, as well as between precision and true positive rate (recall). Moreover, the areas under the ROC curves in (**a**) decrease with increasing noise, ranging between 0.947 and 0.912 for $3 \leq$ sd ≤ 3.5, indicating that Nanopolish[1] exhibits in our simulations only a 91.2% to 94.7% chance of distinguishing between truly methylated and truly unmethylated CpG sites at those noise levels. Notably, and by using real data, Yuen et al. [Systematic benchmarking of tools for CpG methylation detection from Nanopore sequencing. *Nat. Commun.* **12**, 3438 (2021)] reported an area under the ROC curve of 0.921 for Nanopolish[1]. A similar remark is also true for the areas under the PR curves in (**b**), which range between 0.949 and 0.931 when $3 \leq$ sd ≤ 3.5, as compared to the value of 0.924 reported by Yuen et al. Interestingly, the area under the PR curve can be interpreted as the fraction of true detections made by a randomly selected threshold [Boyd, K., Eng, K. H. & Page C. D. Area under the precision-recall curve: Point estimates and confidence intervals. In: Machine Learning and Knowledge Discovery in Databases, Lecture Notes in Computer Science, vol. 8190 (Springer, Berlin, 2013)]. We therefore conclude that, when $3 \leq$ sd ≤ 3.5, Nanopolish[1] exhibits only a 93.1% to 94.9% chance that calls at individual CpG sites made by a randomly selected detection threshold will be true methylation calls.

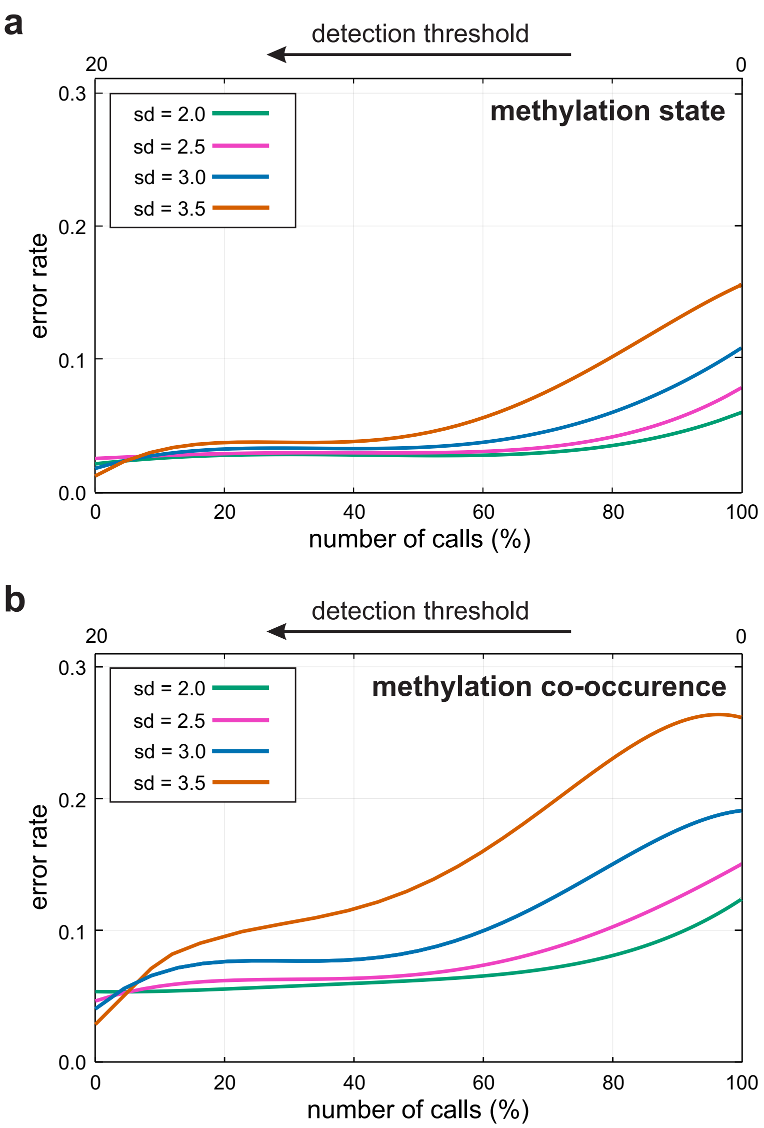

Figure S3. Methylation calling performance of Nanopolish[1]. (**a**) Error rates in calling the true methylation state at individual CpG sites for different levels of nanopore noise, as a function of the detection threshold of Nanopolish[1] and the number of calls made. (**b**) Error rates in calling the true methylation co-occurrence at pairs of consecutive CpG sites. These results were obtained by using our simulation-based scheme for benchmarking Nanopolish[1] (Supplementary Methods, Fig. 3) and by considering nanopore noise with standard deviations sd $= 2, 2.5, 3, 3.5$. Methylation co-occurrence identifies pairs of consecutive CpG sites that are both methylated or unmethylated.

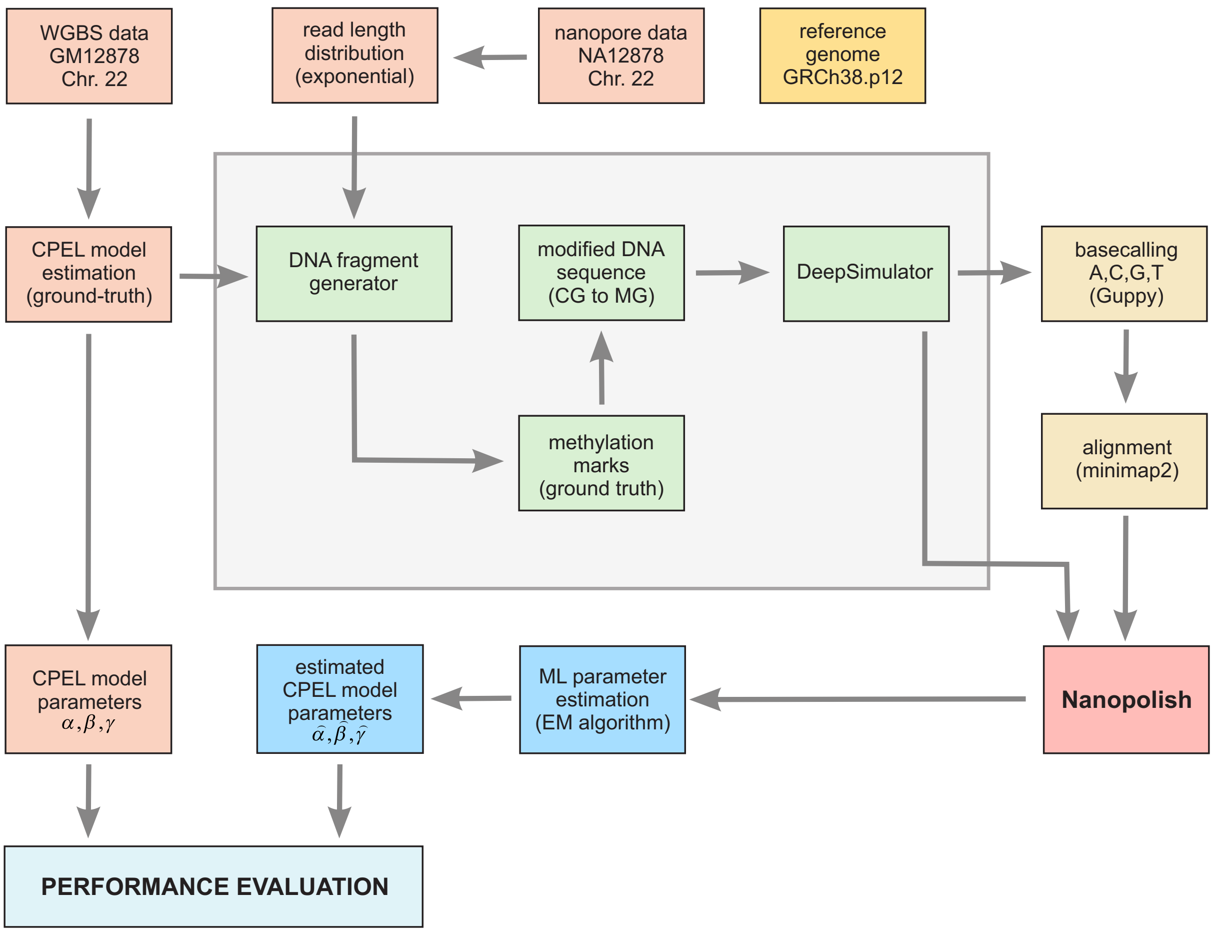

Figure S4. Parameter estimation benchmarking scheme. Simulation-based method for evaluating the EM-based maximum-likelihood parameter estimation module of CpelNano. Within each estimation region of Chr. 22, values of the CPEL model parameters are estimated from simulated data generated by using our simulation-based scheme for benchmarking Nanopolish[1] (Supplementary Methods, Fig. 3). These values are then compared to "true" values, which are computed by fitting the CPEL model to GM12878 WGBS data. Performance evaluation is carried out by computing cosine similarities, boxplots, and binned probability distributions.

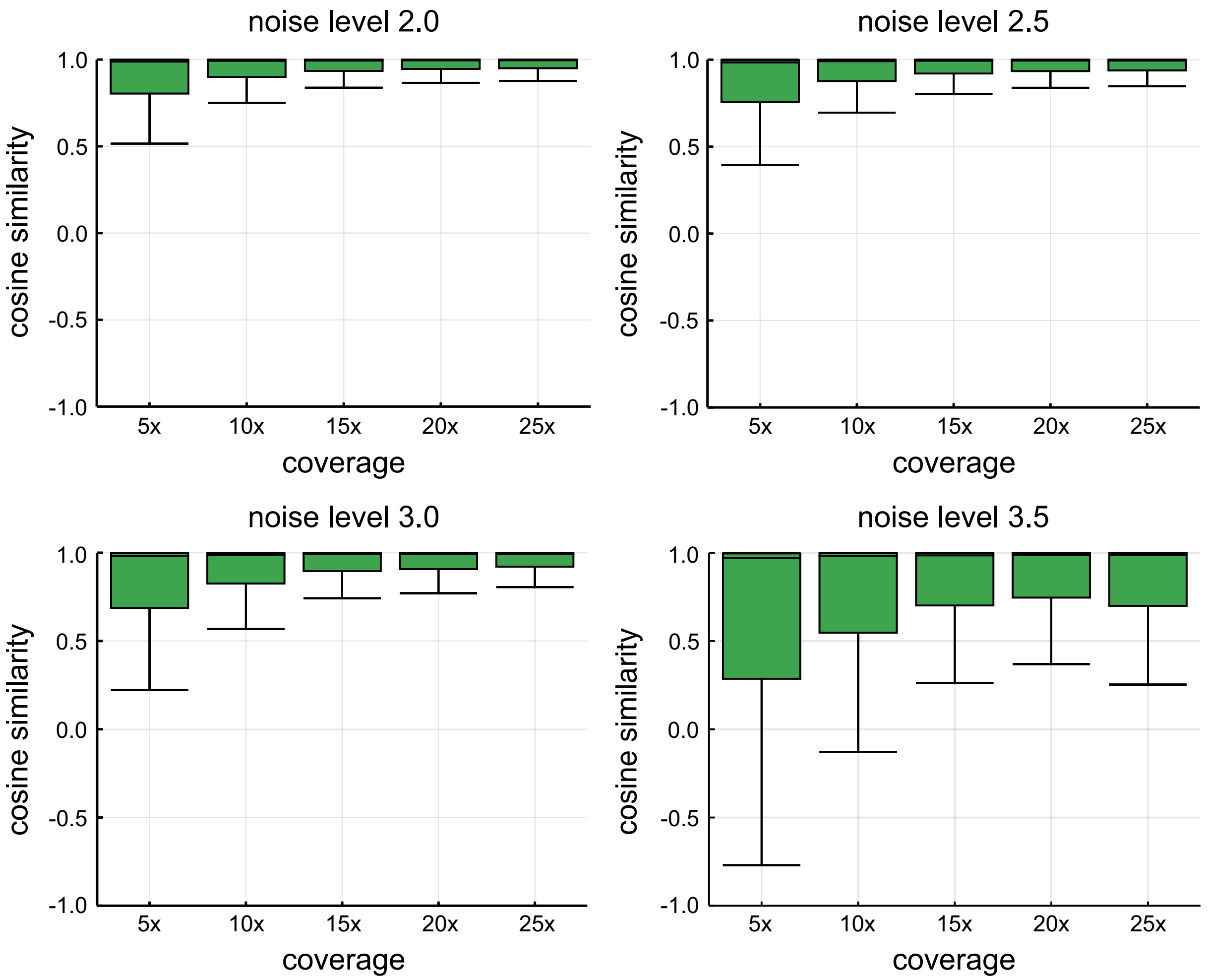

Figure S5. Quality of EM-based maximum-likelihood parameter estimation. Boxplots depicting distributions of cosine similarities when comparing estimated to true values of the CPEL model parameters obtained by using our simulation-based parameter estimation benchmarking scheme (Fig. S4). Results are shown for nanopore noise with standard deviations sd $=$ 2, 2.5, 3, 3.5 and nanopore data with coverages $5\times$, $10\times$, $15\times$, $20\times$, and $25\times$. Center line of box: median value; box bounds: 25th and 75th percentiles; lower whisker: larger of minimum value and 25th percentile minus $1.5\times$ interquartile range; upper whisker: smaller of maximum value and 75th percentile plus $1.5\times$ interquartile range.

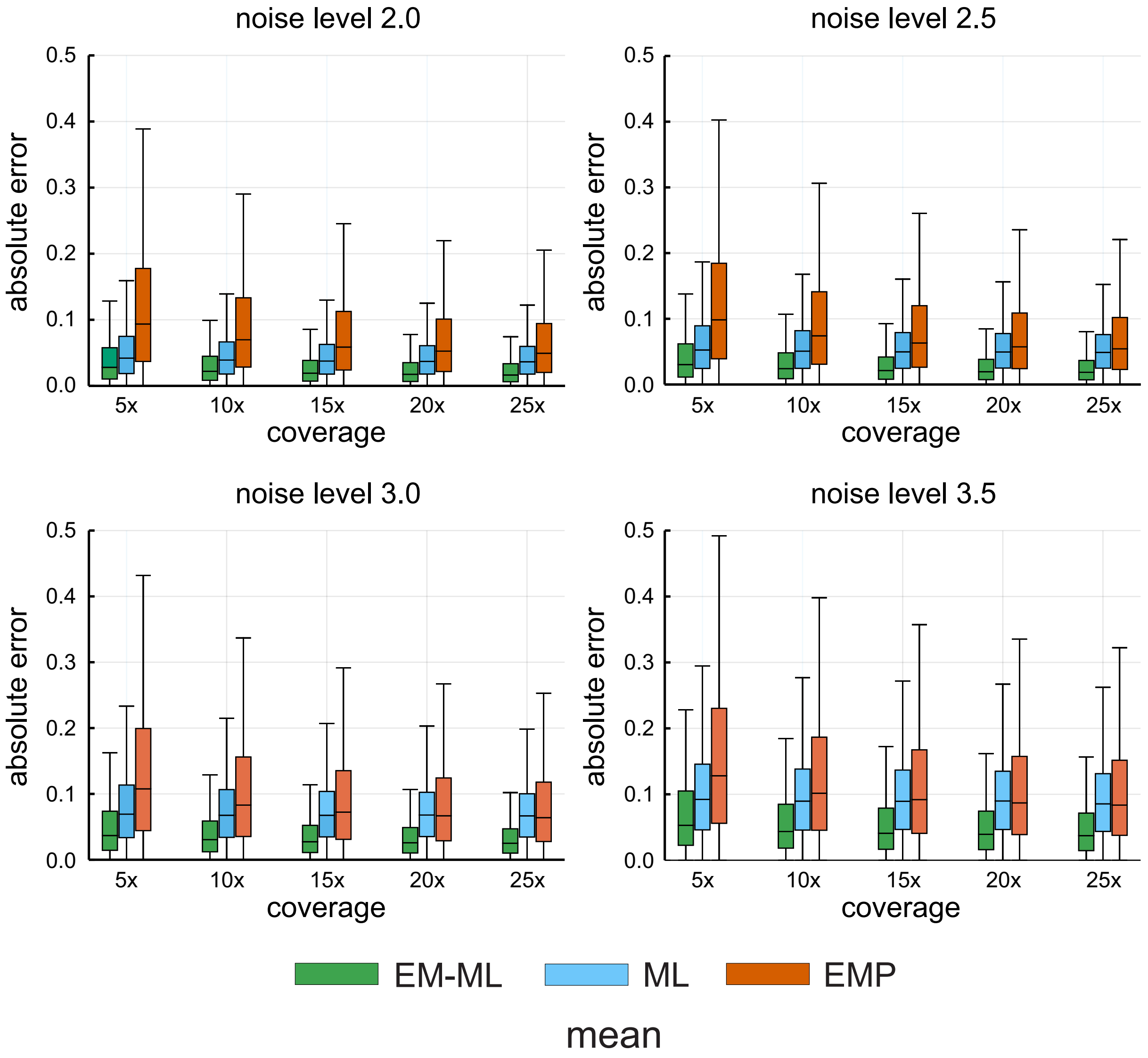

Figure S6. Quality of estimated methylation means. Boxplots depicting distributions of absolute errors between estimated methylation means at individual CpG sites and their true values. Means were estimated by using the EM-based maximum-likelihood (EM-ML) module of CpelNano, as well as by fitting the CPEL model directly to the methylation calls made by Nanopolish[1] using maximum-likelihood (ML) and empirically (EMP) from such calls. Results are shown for nanopore noise with standard deviations sd $= 2, 2.5, 3, 3.5$ and nanopore data coverages of $5\times$, $10\times$, $15\times$, $20\times$, and $25\times$. Center line of box: median value; box bounds: 25th and 75th percentiles; lower whisker: larger of minimum value and 25th percentile minus $1.5\times$ interquartile range; upper whisker: smaller of maximum value and 75th percentile plus $1.5\times$ interquartile range.

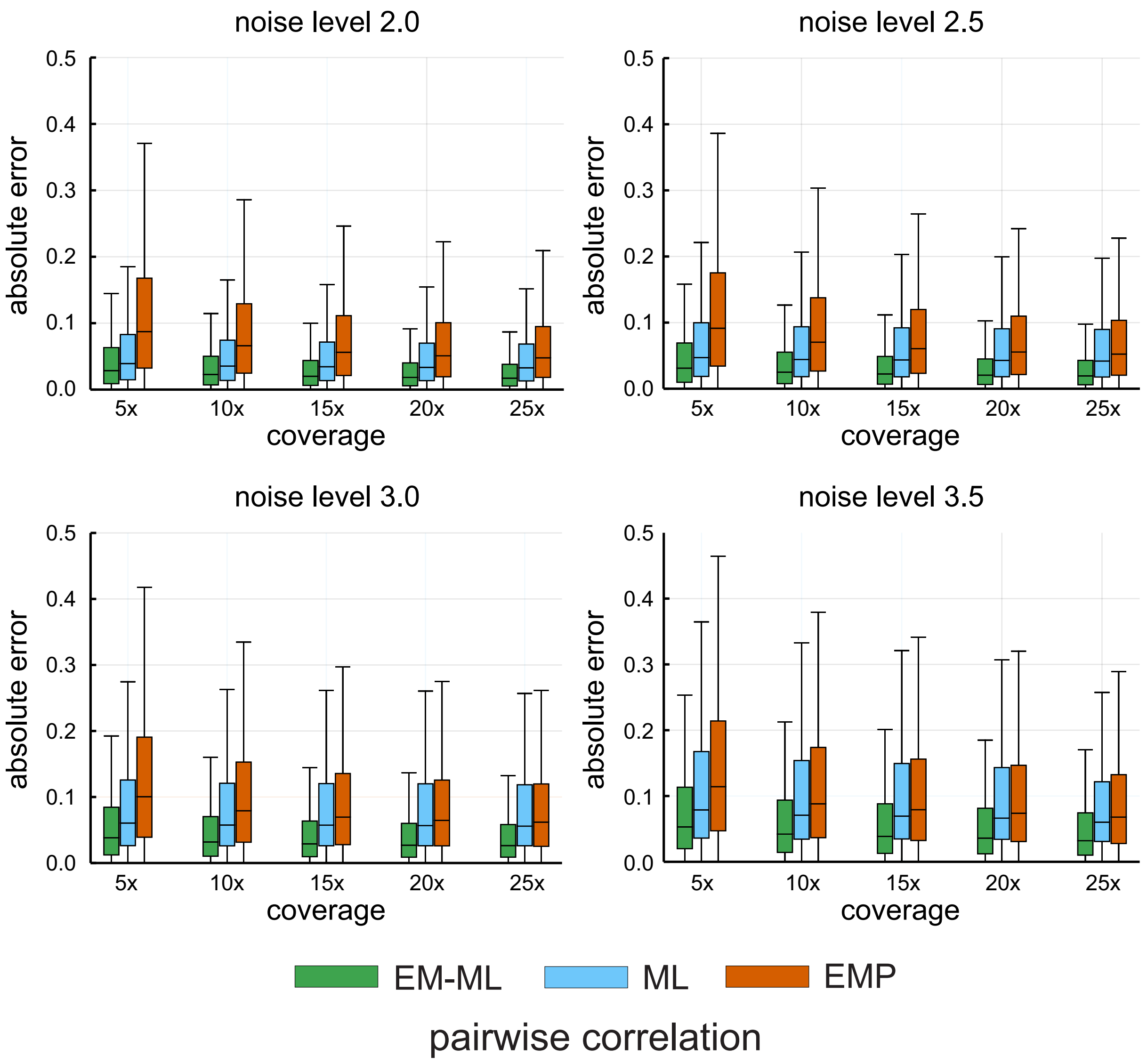

Figure S7. Quality of estimated pairwise correlations. Boxplots depicting distributions of absolute errors between estimated pairwise correlations in methylation and their true values. Correlations were estimated by using the EM-based maximum-likelihood (EM-ML) module of CpelNano, as well as by fitting the CPEL model directly to the methylation calls made by Nanopolish[1] using maximum-likelihood (ML) and empirically (EMP) from such calls. Results are shown for nanopore noise with standard deviations sd $= 2, 2.5, 3, 3.5$ and nanopore data coverages of $5\times$, $10\times$, $15\times$, $20\times$, and $25\times$. Center line of box: median value; box bounds: 25th and 75th percentiles; lower whisker: larger of minimum value and 25th percentile minus $1.5\times$ interquartile range; upper whisker: smaller of maximum value and 75th percentile plus $1.5\times$ interquartile range.

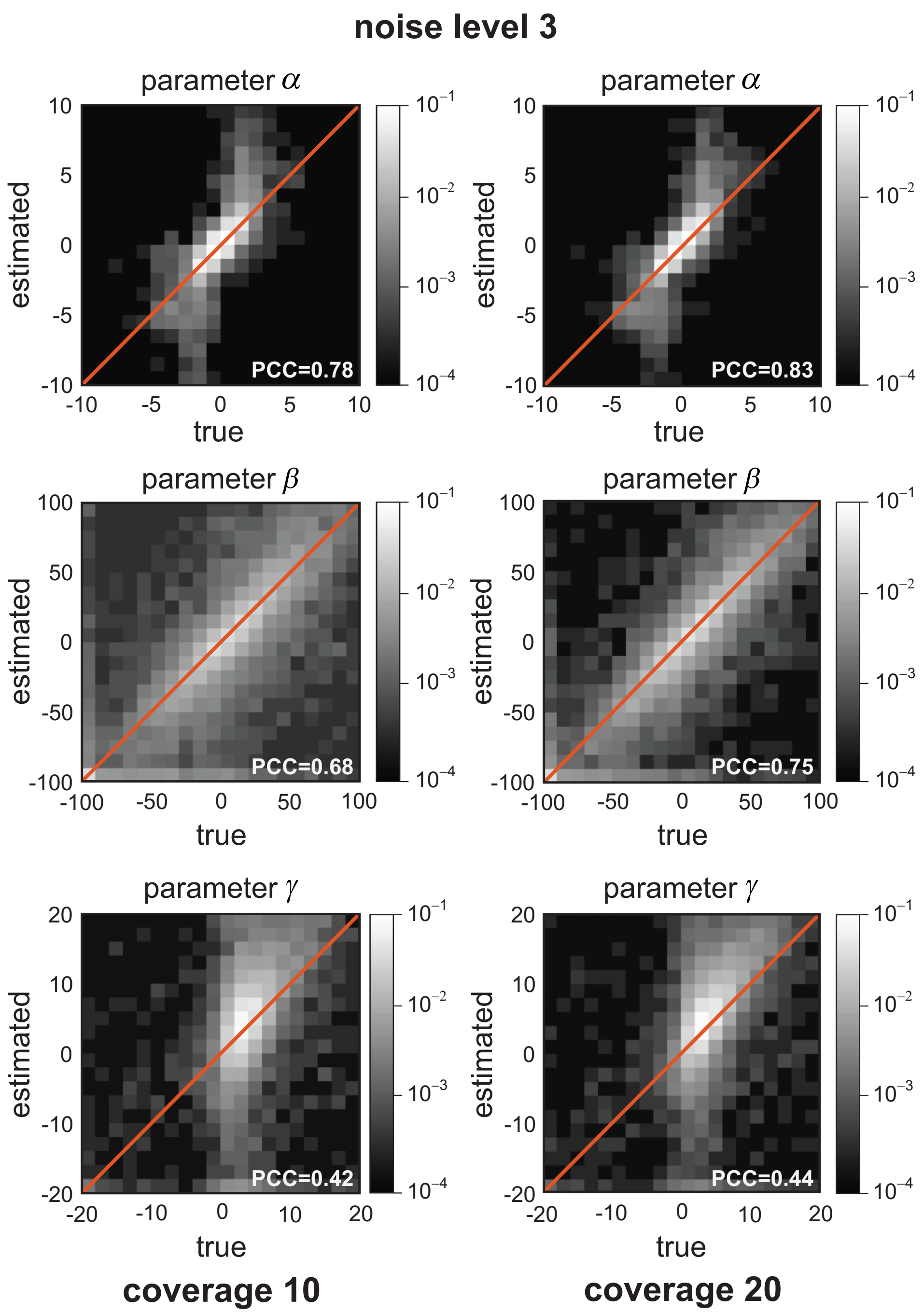

Figure S8. Quality of EM-based maximum-likelihood parameter estimation. Binned joined probability distributions, and associated Pearson correlation coefficient (PCC) values, between estimated CPEL parameter values and true values obtained by using our simulation-based parameter estimation benchmarking scheme (Fig. S4). Results are shown for nanopore noise with standard deviation sd $=3$ and nanopore data coverages of $10\times$ and $20\times$. Lighter regions indicate higher probabilities of association between estimated and true values.

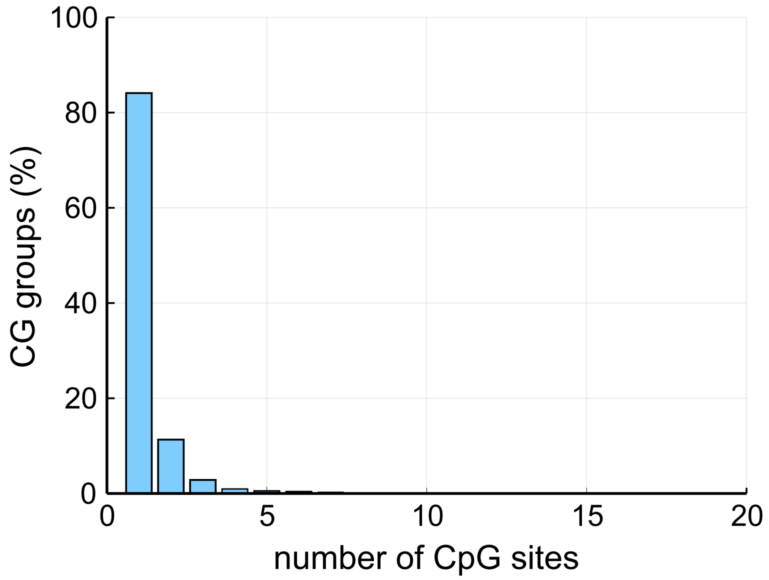

Figure S9. CG-group distribution. The distribution of CG-groups in the human genome in terms of CpG content.

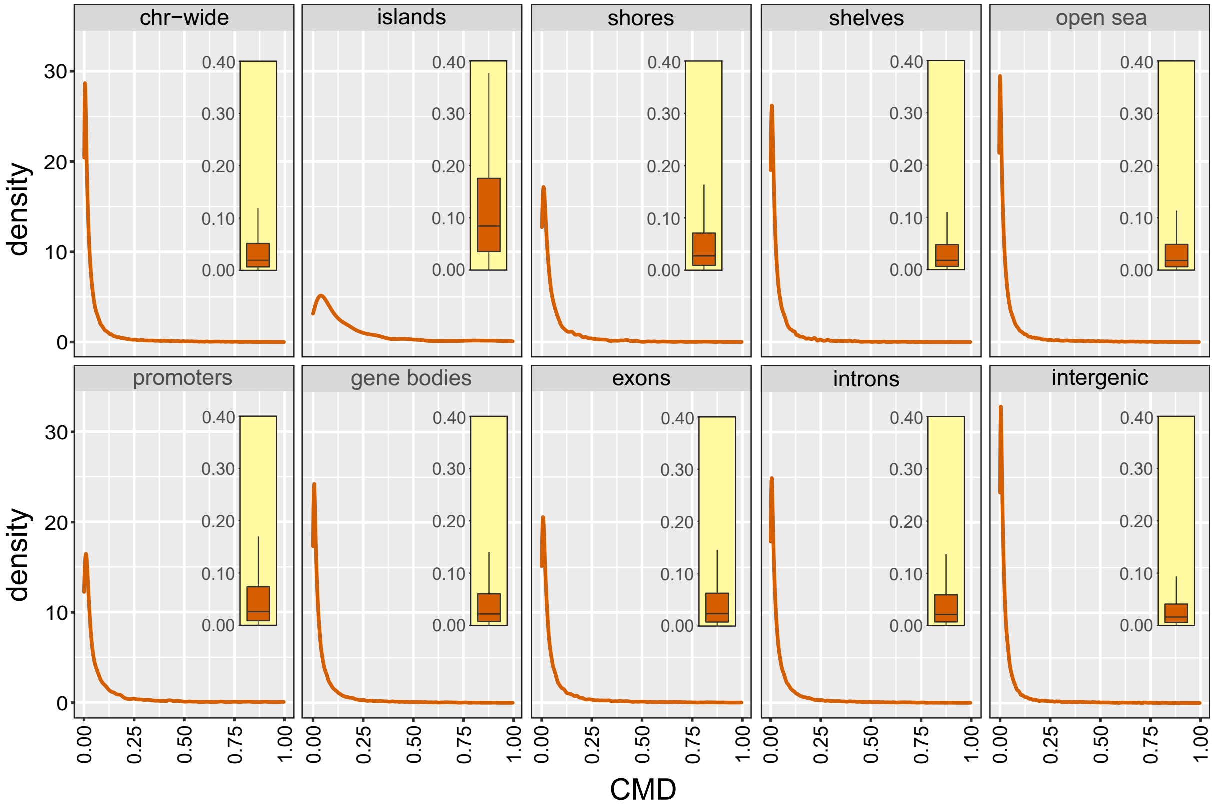

Figure S10. CMD distributions in the Utah/Ceph lymphoblastoid cell line. Densities and boxplot distributions (insets) of coefficient of methylation divergence (CMD) values over selected genomic features of the human genome (Chr. 22) when comparing the probability distributions of methylation estimated by CpelNano using nanopore data (NA12878) and by a standard maximum-likelihood approach using WGBS data (GSM2308632). Center line of box: median value; box bounds: 25th and 75th percentiles; lower whisker: larger of minimum value and 25th percentile minus 1.5× interquartile range; upper whisker: smaller of maximum value and 75th percentile plus 1.5× interquartile range.

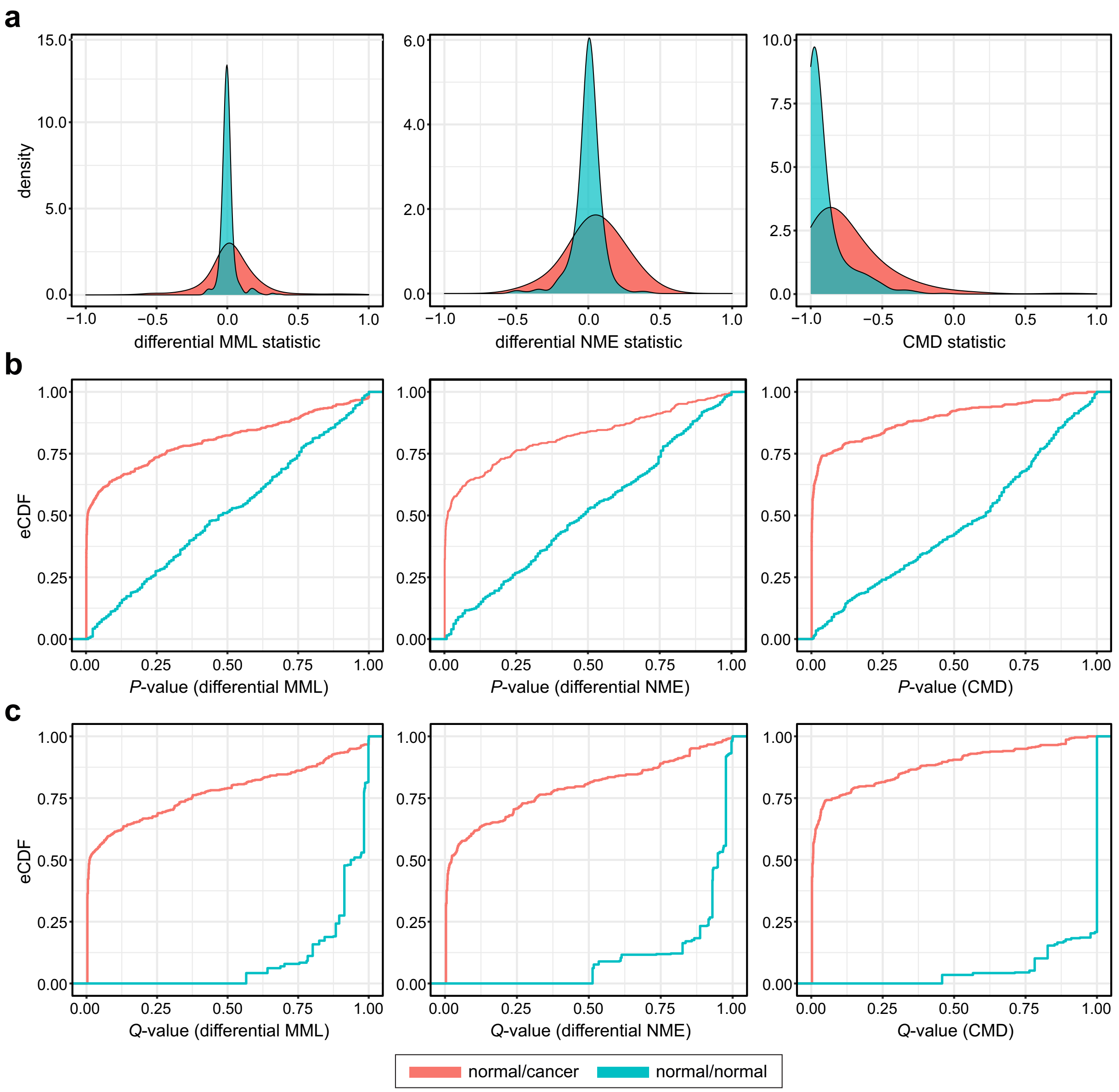

Figure S11. Differential methylation analysis results in the targeted breast normal/cancer comparison. (**a**) Densities of differential mean methylation level (MML), normalized methylation entropy (NME), and average coefficient of methylation difference (CMD) test statistic values in the normal/cancer (red) and the normal/normal (green) comparisons. (**b**) Empirical cumulative distribution functions (eCDFs) of P-values obtained by permutation testing. (**c**) eCDFs of Q-values obtained by the Benjamini-Hochberg procedure for FDR control.

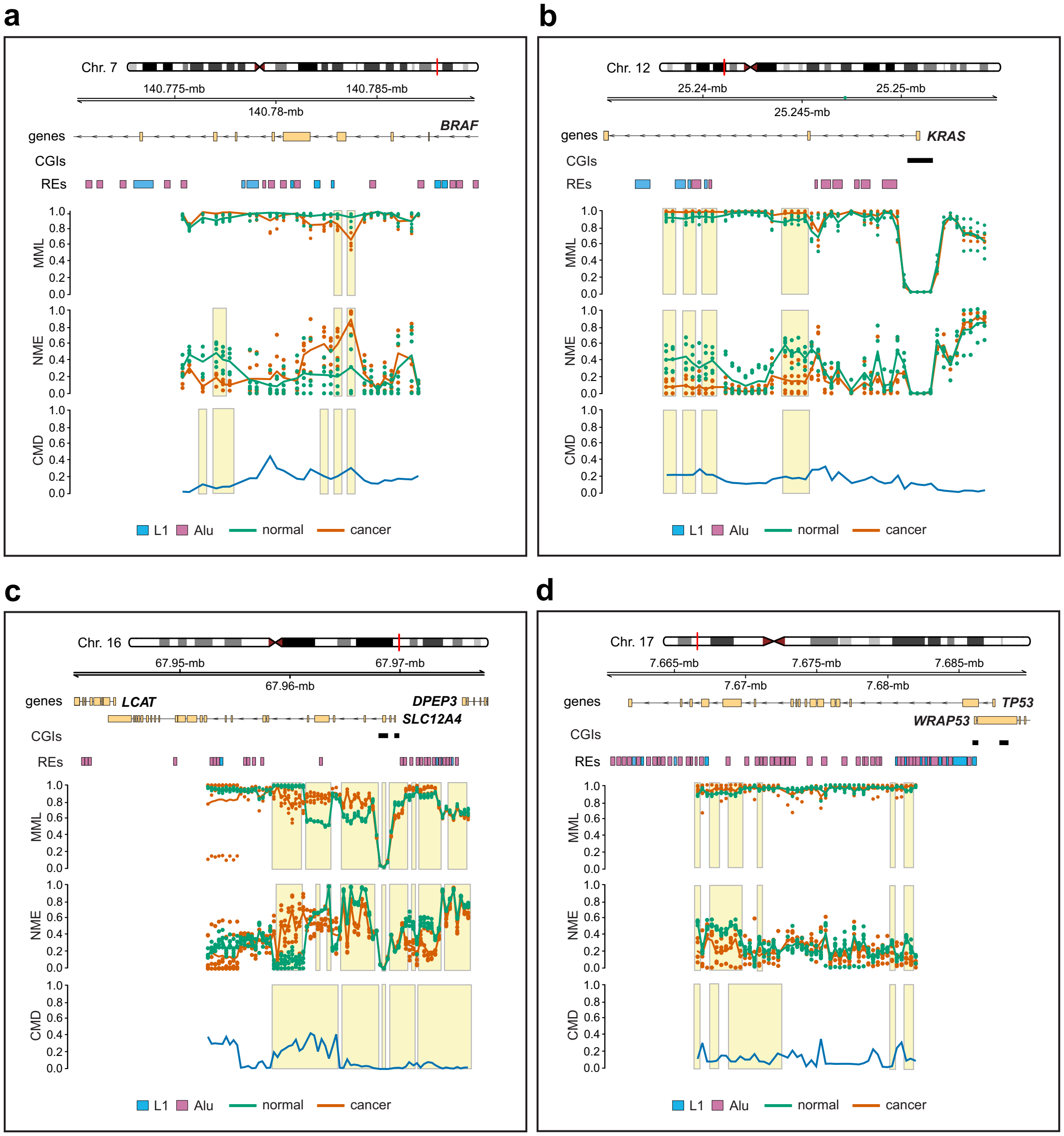

Figure S12. Additional examples of methylation discordance over genes and repetitive elements in the targeted breast normal/cancer comparison. (**a**) Averages of mean methylation levels (MMLs) and normalized methylation entropies (NMEs) over genomic regions overlapping *BRAF*, observed in two groups of five "normal" (green lines) and five "cancer" (red lines) samples used for differential analysis. The average of all differences in the probability distributions of methylation between the two groups, quantified by the coefficient of methylation divergence (CMD), is also depicted (blue line). Dots indicate individual MML and NME values for each group and sample, whereas boxes delineate genomic regions of significant ($q \leq 0.05$) DNA methylation discordance. CGIs track: CpG islands; REs track: L1 (blue) and Alu (purple) repetitive elements. (**b**) Results of methylation discordance associated with *KRAS*. (**c**) Results of methylation discordance associated with *SLC12A4*. (**d**) Results of methylation discordance associated with *TP53*.

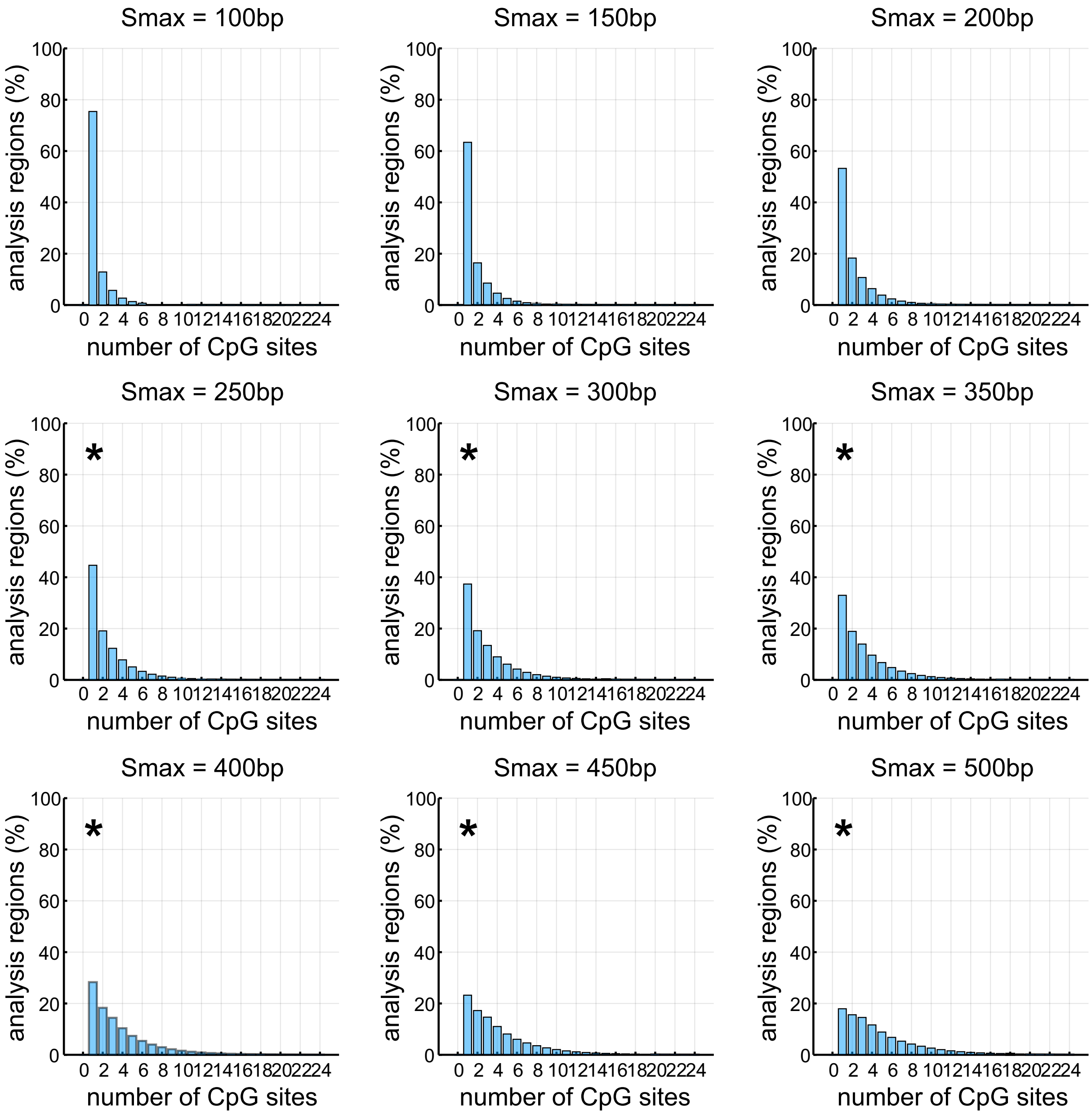

Figure S13. Distribution of analysis regions in terms of CpG population. Histograms of CpG site populations within analysis regions of the human genome for different values of $s_{\max}$. Cases for which the majority of the analysis regions contain more than one CpG site are marked with a star.

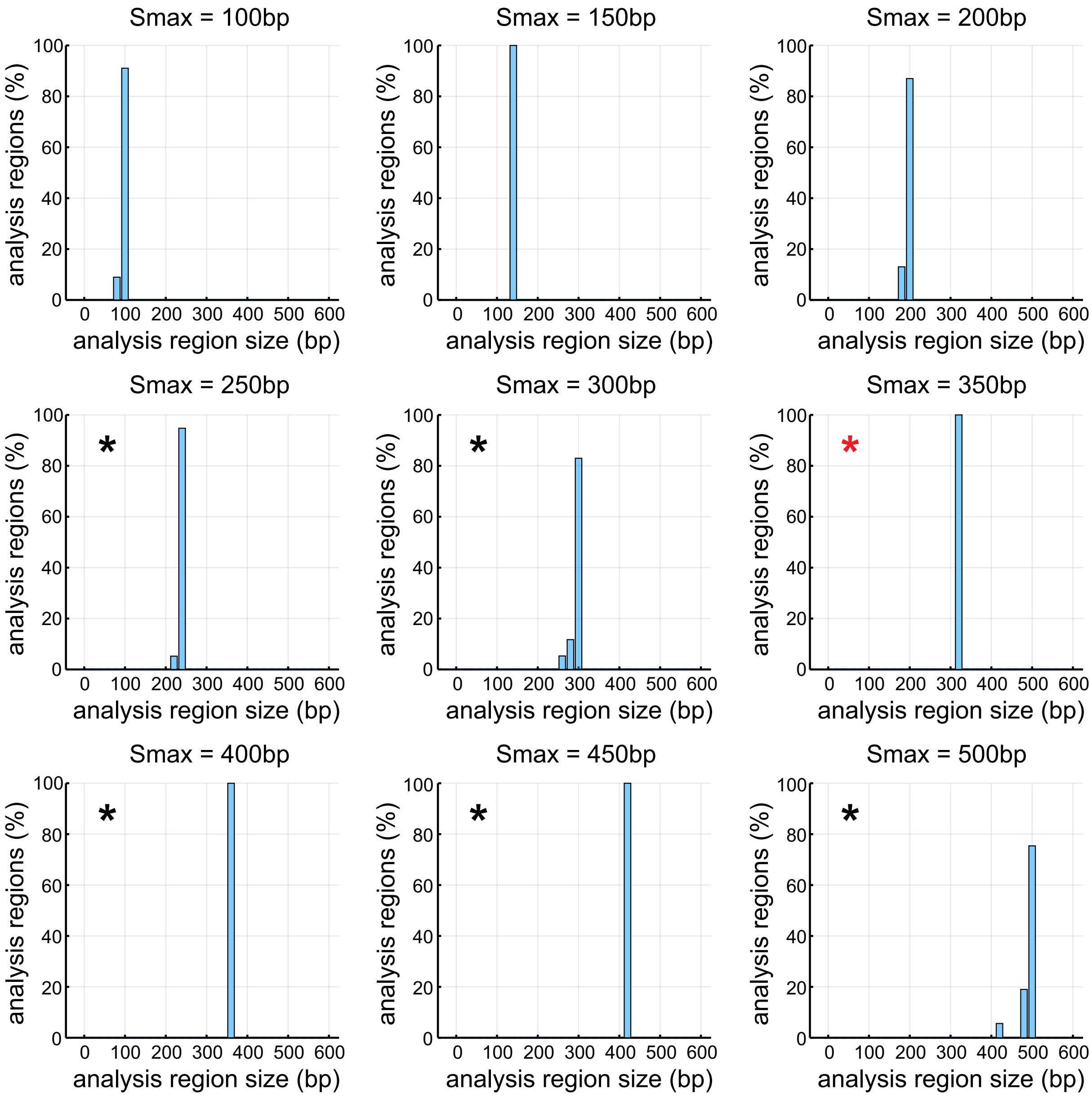

Figure S14. Distribution of analysis regions in terms of size. Histograms of the sizes of the analysis regions in the human genome for different values of $s_{\max}$. The case of $s_{\max} = 350$ bp (red star) is associated with the smallest value of $s_{\max}$ for which the majority of the analysis regions contain more than one CpG site while their sizes exhibit the least variation (see also Fig. S13).

Supplementary Tables

Table S1. Methylation discordance and genes in the targeted breast normal/cancer comparison. The (annotated) body of each listed gene was found to overlap with analysis regions exhibiting patterns (each row) of significant discordance (marked by ⋆) in mean methylation level (MML), normalized methylation entropy (NME), or in the probability distribution of methylation quantified by the coefficient of methylation divergence (CMD). Highlighted genes are fully covered by the data.

Chr.	Start	End	Gene	MML	NME	CMD	#
7	140719327	140924928	*BRAF*	–	–	⋆	3
				–	⋆	⋆	2
				⋆	⋆	⋆	2
9	35673918	35681159	*CA9*	–	⋆	–	1
				⋆	⋆	⋆	1
3	49357176	49358358	*GPX1*	–	⋆	–	1
				–	–	⋆	3
				–	⋆	⋆	1
11	67583595	67586656	*GSTP1*	–	–	⋆	3
				⋆	–	⋆	1
				⋆	⋆	⋆	4
12	25205246	25250936	*KRAS*	⋆	⋆	⋆	10
17	41513745	41522529	*KRT15*	–	–	⋆	4
				⋆	–	⋆	4
				⋆	⋆	⋆	6
17	41523617	41528308	*KRT19*	–	–	⋆	2
				⋆	–	⋆	9
				⋆	⋆	⋆	4
17	49359145	49412097	*RHOA*	⋆	–	⋆	2
				⋆	⋆	⋆	14
16	67943474	67969601	*SLC12A4*	–	–	⋆	4
				⋆	–	⋆	6
				⋆	⋆	⋆	21
17	7661779	7687550	*TP53*	–	⋆	–	2
				–	–	⋆	7
				⋆	⋆	⋆	10
9	35681992	35690056	*TPM2*	⋆	–	–	1
				–	–	⋆	4
				⋆	⋆	–	1
				⋆	–	⋆	2
				⋆	⋆	⋆	9

Table S2. Methylation discordance and promoter regions in the targeted breast normal/cancer comparison. The promoter region (annotated) of each listed gene was found to overlap with analysis regions exhibiting patterns (each row) of significant discordance (marked by ⋆) in mean methylation level (MML), normalized methylation entropy (NME), or in the probability distribution of methylation quantified by the coefficient of methylation divergence (CMD). Highlighted genes are fully covered by the data.

Chr.	Start	End	Gene	MML	NME	CMD	#
3	49356359	49360358	*GPX1*	–	⋆	–	1
				–	–	⋆	3
				–	⋆	⋆	1
				⋆	⋆	⋆	6
11	67581595	67585594	*GSTP1*	–	–	⋆	3
				⋆	–	⋆	5
				⋆	⋆	⋆	5
17	41520530	41524529	*KRT15*	–	–	⋆	2
				⋆	–	⋆	6
				–	⋆	⋆	1
				⋆	⋆	⋆	2
17	41526309	41530308	*KRT19*	⋆	–	⋆	8
				–	⋆	⋆	1
				⋆	⋆	⋆	2
16	67967602	67971601	*SLC12A4*	–	–	⋆	1
				⋆	–	⋆	1
				⋆	⋆	⋆	8
9	35688057	35692056	*TPM2*	⋆	–	⋆	1
				⋆	⋆	⋆	11

Table S3. Methylation discordance and repetitive elements in the targeted breast normal/cancer comparison. The listed repetitive elements (REs) were found to overlap with analysis regions exhibiting patterns (each row) of significant discordance (marked by ⋆) in mean methylation level (MML), normalized methylation entropy (NME), or in the probability distribution of methylation quantified by the coefficient of methylation divergence (CMD). Highlighted REs are Alu (purple) or L1 (blue) repeats.

Chr.	Start	End	RE	MML	NME	CMD	#
3	49,353,229	49,353,360	L2b	–	–	⋆	1
3	49,353,229	49,353,360	L2b	⋆	⋆	⋆	1
3	49,353,360	49,353,657	AluY	⋆	⋆	⋆	2
3	49,353,659	49,353,960	AluSx1	⋆	⋆	⋆	2
3	49,353,960	49,354,019	L2b	⋆	⋆	⋆	1
3	49,354,019	49,354,337	AluYb8	⋆	⋆	⋆	2
3	49,354,653	49,354,960	AluSx1	–	–	⋆	1
3	49,354,653	49,354,960	AluSx1	⋆	⋆	⋆	1
3	49,355,133	49,355,409	AluSx1	–	–	⋆	1
3	49,355,133	49,355,409	AluSx1	⋆	⋆	⋆	1
3	49,356,824	49,357,137	AluY	–	⋆	–	1
3	49,357,992	49,358,002	(CCGCC)n	–	–	⋆	1
3	49,358,240	49,358,260	(GCC)n	–	–	⋆	1
3	49,360,467	49,360,757	AluSq2	⋆	⋆	⋆	2
3	49,360,793	49,361,101	AluSc	⋆	⋆	⋆	2
3	49,361,600	49,361,893	AluSp	⋆	⋆	⋆	2
3	49,361,905	49,362,198	AluSx1	⋆	⋆	⋆	1
3	49,362,667	49,362,724	L3	⋆	⋆	⋆	1
3	49,363,136	49,363,427	AluY	⋆	–	⋆	1
3	49,363,136	49,363,427	AluY	⋆	⋆	⋆	1
3	49,363,452	49,363,575	AluJo	⋆	⋆	⋆	1
3	49,363,575	49,363,869	AluSx	⋆	⋆	⋆	2
3	49,363,869	49,364,065	AluJo	⋆	⋆	⋆	2
3	49,364,067	49,364,204	AluSx	⋆	⋆	⋆	1
3	49,364,204	49,364,511	AluSp	⋆	⋆	⋆	2
3	49,364,511	49,364,661	AluSx	⋆	⋆	⋆	1
7	140,777,229	140,777,809	L3	–	–	⋆	1
7	140,777,229	140,777,809	L3	–	⋆	⋆	1
7	140,782,463	140,782,492	A-rich	–	–	⋆	1
7	154,597,321	154,597,622	AluSc8	⋆	⋆	⋆	1
7	154,597,630	154,598,086	MLT1C	⋆	⋆	⋆	3
7	154,598,803	154,598,849	(TTGTT)n	⋆	⋆	⋆	1
7	154,599,291	154,599,413	MIRc	⋆	⋆	⋆	2

Continued on next page

Chr.	Start	End	RE	MML	NME	CMD	#
7	154,599,494	154,599,528	(TTA)n	⋆	⋆	⋆	1
7	154,599,528	154,599,865	L1PA6	⋆	⋆	⋆	2
7	154,602,840	154,603,384	L1MEf	–	–	⋆	1
7	154,603,384	154,603,683	AluSq2	–	–	⋆	1
7	154,603,384	154,603,683	AluSq2	⋆	⋆	⋆	1
7	154,603,683	154,603,742	L1MEf	⋆	⋆	⋆	1
7	154,603,743	154,604,043	L1ME3A	⋆	⋆	⋆	1
7	154,606,614	154,607,278	Tigger2b_Pri	⋆	–	–	1
7	154,606,614	154,607,278	Tigger2b_Pri	⋆	⋆	⋆	1
7	154,607,278	154,607,588	AluY	⋆	–	–	2
7	154,607,588	154,607,930	Tigger2b_Pri	⋆	–	–	1
7	154,607,588	154,607,930	Tigger2b_Pri	⋆	⋆	⋆	1
7	154,607,968	154,608,273	AluSc8	⋆	⋆	–	1
7	154,608,320	154,608,341	(AT)n	⋆	⋆	–	1
7	154,608,341	154,608,635	AluSz	⋆	⋆	–	1
7	154,608,341	154,608,635	AluSz	⋆	⋆	⋆	1
7	154,608,894	154,609,206	L1MEd	⋆	⋆	⋆	1
7	154,611,941	154,612,247	MSTD	⋆	⋆	⋆	1
7	154,612,247	154,612,841	L1MD2	⋆	⋆	⋆	3
7	154,612,861	154,613,220	MLT1A	⋆	⋆	⋆	2
7	154,613,339	154,613,649	AluSp	⋆	–	⋆	1
9	35,680,372	35,680,526	FRAM	–	⋆	–	1
9	35,681,312	35,681,539	MLT1D	⋆	⋆	⋆	2
9	35,681,586	35,681,616	(TG)n	⋆	⋆	⋆	1
9	35,683,272	35,683,374	GA-rich	⋆	⋆	⋆	1
9	35,686,528	35,686,656	FLAM_A	⋆	–	⋆	1
9	35,686,939	35,687,123	MIR	⋆	–	–	1
9	35,687,187	35,687,307	MER5A	⋆	–	–	1
9	35,687,187	35,687,307	MER5A	⋆	⋆	⋆	1
9	35,690,580	35,690,619	(CCTCC)n	⋆	⋆	⋆	1
9	35,690,734	35,690,816	(CCCCG)n	⋆	⋆	⋆	1
9	35,690,816	35,690,846	(CGCTCCC)n	⋆	⋆	⋆	1
9	35,690,925	35,690,970	(GGAGGC)n	⋆	⋆	⋆	1
9	35,691,254	35,691,305	(GCCGTGG)n	⋆	⋆	⋆	1
9	35,691,709	35,691,829	FLAM_A	⋆	⋆	⋆	1
9	35,691,840	35,692,353	L2c	⋆	⋆	⋆	3
9	35,692,407	35,692,805	Charlie1a	⋆	⋆	⋆	2
9	35,692,807	35,693,115	AluSz	⋆	⋆	⋆	1
9	35,693,622	35,693,926	AluJb	–	–	⋆	1

Continued on next page

Chr.	Start	End	RE	MML	NME	CMD	#
9	35,693,926	35,694,020	Charlie1a	–	–	⋆	1
9	35,694,020	35,694,324	AluSx	–	⋆	–	1
9	35,694,020	35,694,324	AluSx	–	–	⋆	1
9	35,694,324	35,694,485	Charlie1a	–	–	⋆	1
9	35,694,485	35,694,770	AluSx1	–	⋆	–	2
9	35,694,770	35,694,798	(AAAT)n	–	⋆	–	1
9	35,694,808	35,695,277	Charlie1a	–	⋆	–	1
9	35,694,808	35,695,277	Charlie1a	⋆	⋆	⋆	2
9	35,695,278	35,695,579	AluSg	⋆	⋆	⋆	1
11	67,577,502	67,577,623	MIR	⋆	⋆	⋆	1
11	67,577,990	67,578,406	MamGyp-int	–	–	⋆	1
11	67,577,990	67,578,406	MamGyp-int	⋆	–	⋆	1
11	67,577,990	67,578,406	MamGyp-int	⋆	⋆	⋆	1
11	67,579,281	67,579,440	L1MEh	⋆	–	⋆	1
11	67,579,646	67,579,904	L1MEh	⋆	–	⋆	2
11	67,579,991	67,580,258	L1MEh	⋆	–	⋆	1
11	67,579,991	67,580,258	L1MEh	⋆	⋆	⋆	1
11	67,580,258	67,580,551	AluSq	⋆	⋆	⋆	2
11	67,580,551	67,580,847	L1MEh	⋆	⋆	⋆	2
11	67,580,876	67,580,898	(A)n	⋆	⋆	⋆	1
11	67,580,901	67,581,040	AluJb	⋆	⋆	⋆	2
11	67,581,058	67,582,018	MER11C	⋆	–	⋆	2
11	67,581,058	67,582,018	MER11C	⋆	⋆	⋆	1
11	67,582,055	67,582,412	L1PA14	⋆	–	⋆	1
11	67,582,055	67,582,412	L1PA14	⋆	⋆	⋆	1
11	67,582,471	67,582,653	L1M5	⋆	⋆	⋆	1
11	67,582,665	67,583,019	L1PA11	⋆	–	⋆	1
11	67,582,665	67,583,019	L1PA11	⋆	⋆	⋆	1
11	67,583,020	67,583,297	AluSx	⋆	–	⋆	1
11	67,583,020	67,583,297	AluSx	⋆	⋆	⋆	1
11	67,583,297	67,583,405	(ATAAA)n	–	–	⋆	1
11	67,583,297	67,583,405	(ATAAA)n	⋆	⋆	⋆	1
11	67,584,869	67,584,914	MIR	⋆	–	⋆	1
11	67,585,658	67,585,700	(GT)n	⋆	⋆	⋆	1
11	67,589,083	67,589,373	THE1D	–	–	⋆	1
11	67,589,373	67,589,531	MER65-int	–	–	⋆	1
11	67,589,949	67,590,022	MER65-int	⋆	⋆	⋆	1
11	67,589,990	67,590,061	MER57A-int	⋆	⋆	⋆	1
11	67,591,388	67,591,691	AluSq2	–	–	⋆	1

Continued on next page

Chr.	Start	End	RE	MML	NME	CMD	#
11	67,591,854	67,592,393	LTR49-int	⋆	⋆	⋆	1
11	67,593,508	67,593,973	LTR22A	⋆	–	⋆	1
11	67,593,508	67,593,973	LTR22A	⋆	⋆	⋆	1
11	67,593,973	67,594,258	LTR1	⋆	⋆	⋆	1
12	25,238,658	25,239,260	L1ME4b	⋆	⋆	⋆	1
12	25,240,034	25,240,102	L1MC4a	⋆	⋆	⋆	1
12	25,240,103	25,240,176	FLAM_C	⋆	⋆	⋆	1
12	25,240,332	25,240,527	MER2B	⋆	⋆	⋆	1
12	25,240,539	25,240,654	L2a	⋆	⋆	⋆	1
12	25,244,206	25,244,530	L3b	⋆	⋆	⋆	2
16	67,958,376	67,958,757	L2	–	–	⋆	1
16	67,958,376	67,958,757	L2	⋆	⋆	⋆	1
16	67,959,406	67,959,487	MIRc	⋆	⋆	⋆	1
16	67,961,135	67,961,341	MIRb	–	–	⋆	1
16	67,962,559	67,962,859	AluSx1	⋆	–	⋆	1
16	67,962,559	67,962,859	AluSx1	⋆	⋆	⋆	1
16	67,965,259	67,965,501	L4_C_Mam	⋆	⋆	⋆	2
16	67,965,773	67,965,888	MIRb	⋆	⋆	⋆	1
16	67,966,039	67,966,204	MIRc	⋆	⋆	⋆	1
16	67,967,727	67,967,874	MIR	⋆	–	⋆	1
16	67,969,984	67,970,273	AluSq2	⋆	⋆	⋆	2
16	67,970,280	67,970,585	AluSx1	⋆	⋆	⋆	2
16	67,970,760	67,970,841	L2c	–	–	⋆	1
16	67,970,868	67,971,163	AluJr	–	–	⋆	1
16	67,970,868	67,971,163	AluJr	⋆	⋆	⋆	1
16	67,971,449	67,971,744	AluY	⋆	⋆	⋆	1
16	67,971,796	67,972,064	AluSx1	⋆	⋆	⋆	2
16	67,972,072	67,972,131	Alu	⋆	⋆	⋆	1
16	67,972,131	67,972,168	(AAAT)n	⋆	⋆	⋆	1
16	67,972,175	67,972,500	AluSz6	⋆	⋆	⋆	2
16	67,972,503	67,972,801	AluSq2	⋆	⋆	⋆	2
16	67,972,933	67,973,231	AluSq2	⋆	⋆	⋆	2
16	67,973,237	67,973,384	L1MB5	⋆	⋆	⋆	2
16	67,973,384	67,971,672	AluY	⋆	⋆	⋆	1
16	67,973,672	67,973,704	(AAAT)n	–	–	⋆	1
16	67,973,672	67,973,704	(AAAT)n	⋆	⋆	⋆	1
16	67,973,704	67,973,745	L1MB5	–	–	⋆	1
16	67,973,806	67,974,103	AluJb	–	–	⋆	1
16	67,973,806	67,974,103	AluJb	–	⋆	⋆	1

Continued on next page

Chr.	Start	End	RE	MML	NME	CMD	#
16	67,974,124	67,974,423	AluY	–	⋆	⋆	1
16	67,974,124	67,974,423	AluY	⋆	⋆	⋆	1
16	67,974,424	67,974,648	AluSz6	⋆	⋆	⋆	1
16	67,974,648	67,974,773	L1M5	⋆	⋆	⋆	2
16	67,975,040	67,975,346	AluSc8	⋆	⋆	⋆	1
17	7,666,567	7,666,868	AluSq2	⋆	⋆	⋆	1
17	7,668,548	7,668,856	AluJb	–	⋆	–	1
17	7,668,548	7,668,856	AluJb	⋆	⋆	⋆	1
17	7,669,141	7,669,244	MIRb	⋆	⋆	⋆	1
17	7,669,895	7,670,197	AluSp	–	–	⋆	2
17	7,670,203	7,670,293	MIRc	–	–	⋆	1
17	7,670,752	7,670,858	MER47A	–	–	⋆	1
17	7,670,752	7,670,858	MER47A	⋆	⋆	⋆	1
17	7,670,873	7,671,171	AluSx	–	–	⋆	1
17	7,670,873	7,671,171	AluSx	⋆	⋆	⋆	1
17	7,671,172	7,671,300	FLAM_C	⋆	⋆	⋆	1
17	7,671,300	7,671,599	AluSx1	–	–	⋆	2
17	7,671,599	7,671,635	AluJb	–	–	⋆	1
17	7,671,660	7,671,815	Tigger5	–	–	⋆	2
17	7,671,815	7,672,107	AluJo	–	–	⋆	1
17	7,672,110	7,672,392	AluSx1	–	–	⋆	2
17	7,672,392	7,672,430	(AAAAT)n	–	–	⋆	1
17	7,672,430	7,672,590	AluJb	–	–	⋆	1
17	7,680,191	7,680,419	MER2	⋆	⋆	⋆	1
17	7,680,419	7,680,525	L1M5	⋆	⋆	⋆	1
17	7,681,086	7,681,158	L1M5	⋆	⋆	⋆	1
17	7,681,158	7,681,338	AluSq2	⋆	⋆	⋆	1
17	7,681,338	7,681,635	AluSq2	⋆	⋆	⋆	2
17	7,681,635	7,681,777	AluSq2	⋆	⋆	⋆	1
17	7,681,785	7,682,092	AluYm1	⋆	⋆	⋆	1
17	41,517,629	41,517,733	MIR3	⋆	⋆	⋆	1
17	41,517,886	41,518,058	FRAM	–	–	⋆	1
17	41,519,347	41,519,392	(GTGA)n	⋆	⋆	⋆	1
17	41,519,548	41,519,577	(CTCCC)n	⋆	–	⋆	1
17	41,520,124	41,520,292	MIRb	–	–	⋆	1
17	41,520,124	41,520,292	MIRb	⋆	⋆	⋆	1
17	41,520,569	41,520,785	MIRc	⋆	–	⋆	1
17	41,521,337	41,521,374	(GCCCCA)n	–	–	⋆	1
17	41,521,484	41,521,571	MIRb	–	–	⋆	1

Continued on next page

Chr.	Start	End	RE	MML	NME	CMD	#
17	41,521,484	41,521,571	MIRb	⋆	–	⋆	1
17	41,521,635	41,521,806	MIRb	⋆	–	⋆	1
17	41,523,023	41,523,192	MIRb	⋆	–	⋆	1
17	41,523,023	41,523,192	MIRb	–	⋆	⋆	1
17	41,523,175	41,523,208	L2a	⋆	–	⋆	1
17	41,523,175	41,523,208	L2a	–	⋆	⋆	1
17	41,523,236	41,523,437	MIRc	–	⋆	⋆	1
17	41,525,654	41,525,806	MIRb	–	–	⋆	1
17	41,525,959	41,526,273	AluY	⋆	–	⋆	1
17	41,525,959	41,526,273	AluY	⋆	⋆	⋆	1
17	41,526,449	41,526,532	AluJb	⋆	–	⋆	1
17	41,526,449	41,526,532	AluJb	⋆	⋆	⋆	1
17	41,526,562	41,526,872	AluSz	⋆	–	⋆	2
17	41,528,710	41,529,006	AluSz	⋆	–	⋆	1
17	41,528,710	41,529,006	AluSz	⋆	⋆	⋆	1
17	41,529,478	41,529,589	MIRc	⋆	–	⋆	1
17	41,529,478	41,529,589	MIRc	–	⋆	⋆	1
17	41,531,411	41,531,574	MIRb	–	⋆	–	1
17	41,531,636	41,531,714	MER103C	–	⋆	–	1
17	41,531,741	41,531,866	MIR3	–	⋆	–	1
